# Data-driven differentiation analysis of urban high-tech industries: Research on bibliometrics and large language models

**DOI:** 10.1371/journal.pone.0348590

**Published:** 2026-05-14

**Authors:** Hua Song, Jun Zeng, Yang Zheng, Han Huang, Hongyu Wang

**Affiliations:** 1 School of Management, Wuhan University of Technology, Wuhan, China; 2 School of Information Management, Wuhan University, Wuhan, China; University of South Carolina, UNITED STATES OF AMERICA

## Abstract

This study examines inter-city heterogeneity in China’s high-tech industries from a regional innovation systems (RIS) perspective, with a particular focus on how variations in knowledge production, technological application, and actor configurations are associated with divergent urban innovation trajectories. We compile more than 39,000 publications from the Web of Science (WOS) and nearly 10,000 patent records from the national patent database for the period 2016–2025, covering four representative cities—Wuhan, Chengdu, Hangzhou, and Tianjin—and four technological domains: artificial intelligence (AI), fiber-optic communication (FOC), intelligent connected vehicles (ICV), and storage chips (SC). The study develops an integrated analytical framework combining bibliometric analysis, co-word network modeling, collaboration network mapping, and large language model (LLM)–assisted semantic interpretation. LLMs are employed primarily in keyword cleaning, terminology standardization, and topic identification, improving the consistency and interpretability of textual metadata. Visualizations generated using VOSviewer highlight pronounced inter-city differences in technological portfolios, research priorities, and collaboration structures. The results suggest distinct urban innovation configurations across the four cities. Wuhan exhibits strong positioning in FOC and SC, reflecting a combined industry–academy orientation. Hangzhou shows high frontier intensity in AI and ICV, consistent with an industry-led and digitally driven innovation profile. Chengdu demonstrates substantial academic output but comparatively weaker evidence of technological translation, while Tianjin, despite a smaller overall scale, displays notable specialization in applied domains such as brain–computer interfaces and smart port technologies. Rather than replacing quantitative analysis, LLM-assisted interpretation supports the identification and contextualization of these patterns by enhancing semantic coherence and reducing noise in large-scale textual data. Overall, the proposed framework provides a reproducible and scalable approach for examining regional technological differentiation and is applicable to comparative studies of urban innovation systems across different regions and industrial contexts.

## Introduction

As globalization and technological change accelerate, urban high-tech industries exhibit increasingly pronounced heterogeneity [1]. Differences in resource endowments, economic foundations, and policy environments jointly shape city-specific technological structures [2], contributing to the localization and spatial concentration of innovation activities within particular urban contexts. Within this background, RIS theory emphasizes that inter-city technological differentiation is closely associated with three interrelated dimensions: dominant research and development (R&D) actors (e.g., universities versus firms), industrial orientation (hardware-oriented manufacturing versus software- and platform-based industries), and the mechanisms through which scientific knowledge is translated into technological applications [3, 4].

Based on these dimensions, Wuhan, Hangzhou, Chengdu, and Tianjin are selected as representative cases of contrasting innovation system configurations. The case selection follows a most-different systems design, aiming to maximize structural variation in urban innovation systems while holding the national institutional context constant. Rather than pursuing statistical representativeness, this study focuses on cities that display pronounced contrasts in dominant innovation drivers, industrial structures, and knowledge production pathways.

From a common baseline, all four cities are megacities with permanent populations exceeding ten million and rank among the top ten cities nationwide in terms of GDP, indicating strong industrial foundations and substantial agglomerations of innovation resources. At the same time, in terms of regional representation and innovation characteristics, Wuhan, Chengdu, Hangzhou, and Tianjin respectively reflect the economic structures and policy orientations of central, western, eastern, and northern China, thereby exhibiting pronounced diversity in regional innovation systems. Wuhan is characterized by a university- and national-laboratory-led innovation structure, hosting 83 universities and 41 national key laboratories, ranking eighth globally in terms of higher education concentration and second nationally in the number of academicians. Its high-tech industries are predominantly hardware-oriented, with optoelectronic information and automobile manufacturing as core sectors, where research institutions drive targeted industrial transformation through breakthroughs in key technologies. Hangzhou, by contrast, is led by technology firms and platform-based enterprises, with a highly active digital economy and software-oriented industries. Its digital security industry accounts for more than 50% of the global market, and its cross-border e-commerce exports rank first nationwide. Market-driven mechanisms strongly pull research activities toward commercialization, resulting in markedly higher transformation efficiency. Chengdu possesses solid foundations in basic research and a number of strong academic disciplines and research institutions; however, the linkage between scientific output and industrial application remains relatively weak, and mechanisms for university–industry collaboration require further strengthening. Electronic information and biomedicine constitute the core pillars of its high-tech industrial structure. Tianjin, in contrast, exhibits a government-led innovation model, with a strategic focus on aerospace and advanced equipment manufacturing aligned with national priorities. Its aerospace equipment import–export value ranks first nationwide, reflecting a policy-oriented innovation mechanism.

The differentiated development trajectories and specialization paths observed across these four cities not only reflect heterogeneity in regional resource endowments and policy support, but also reveal divergent institutional environments and transformation pathways in processes of industrial upgrading and technological innovation. In this context, a systematic examination of inter-city differences in high-tech technological development carries both theoretical and practical significance. On the one hand, it helps to identify path-dependent mechanisms and potential breakthrough opportunities in industrial evolution and technological change, thereby contributing to a deeper understanding of uneven urban economic development. On the other hand, comparative analysis of representative cities provides an empirical basis for central and local governments to formulate differentiated policies, optimize industrial layouts, and promote regional collaborative innovation. At the same time, it offers practical guidance for firms seeking to identify regional innovation advantages and to design R&D strategies and external collaboration pathways.

In this context, high-tech industries have become a key variable that may either amplify or mitigate inter-city technological disparities [5]. Against the backdrop of increasingly convergent industrial agendas across cities, systematically assessing how the same high-tech industries evolve across different urban contexts is essential for identifying differentiated development patterns. Moreover, industries at different stages of the technology life cycle (TLC) differ markedly in innovation intensity, capital requirements, and policy priorities, which in turn shape their roles within urban technological structures and industrial functions. Based on this consideration, and to enable cross–life-cycle comparison and analysis of urban response mechanisms, this study selects four representative high-tech industries—AI [6], FOC [7], ICV [8], and SC [9]—as focal research domains. These industries not only play a central role in reshaping global economic growth and technological competition, but have also been widely incorporated into the strategic development agendas of major Chinese cities. At the same time, they correspond to different stages of the TLC. Specifically, AI represents an introduction-stage technology, still in an early exploratory phase, with research activities concentrated on foundational models, algorithmic frameworks, and computing power optimization. It exhibits high uncertainty and disruptive potential, with development heavily dependent on policy support and talent concentration. ICV is situated in the growth stage, where technological pathways are becoming clearer and industry standards are gradually taking shape. The deep integration of vehicle manufacturing with communication, perception, and transportation systems has accelerated ecosystem formation, making ICV a key lever for high-end manufacturing upgrading and smart transportation initiatives. FOC has entered the maturity stage, characterized by relatively stable underlying technologies and well-developed industrial chains, showing strong scale and agglomeration effects. At this stage, inter-city competition is increasingly manifested in supporting capabilities, high-end talent concentration, and marginal efficiency of technological iteration. By contrast, SC displays a composite pattern in which decline-stage characteristics coexist with renewed relevance. Although innovation in mature process nodes and mid- to low-end markets has slowed amid rising substitution pressure, recent price surges and supply constraints suggest continued demand and strategic importance, justifying its inclusion in the comparative analysis of urban innovation systems. However, because keyword–taxonomy alignment in this domain is too sparse to support robust taxonomy-based temporal comparison, SC is excluded in that later analytical step; the rationale for this stage-specific treatment is explained further in the Methodology section.

Integrating high-tech industries at different life-cycle stages into a unified analytical framework enables a systematic examination of how cities allocate resources, coordinate innovation actors, and respond institutionally to technological change, thereby providing a theoretical and empirical basis for understanding inter-city divergence in industrial evolution.

Despite the growing body of literature on urban and regional technological differentiation—particularly studies employing bibliometric and network-based approaches to examine innovation activity, collaboration networks, and technological hotspots—several limitations remain in terms of methodological design and explanatory power. First, keyword identification and classification often rely on researchers’ experience or simple frequency statistics, lacking unified technical classification standards. As a result, such approaches struggle to capture the hierarchical structure and evolutionary pathways of technology. A particularly salient issue is that multiple lexical forms frequently refer to the same concept (e.g., “convolutional neural network”, “convolutional neural-networks” and “CNN”), and without systematic normalization, technological hotspots are easily fragmented, leading to biased or partial conclusions. Second, existing methods generally lack mechanisms to assess the relative importance or breakthrough potential of technologies, making it difficult to distinguish auxiliary generic tools from key drivers of industrial upgrading and regional leadership. Finally, result interpretation often depends heavily on researchers’ subjective judgment, which can lead to partial explanations and limits the integration of empirical findings with local industrial contexts and policy environments.

To address these limitations, this study introduces two methodological refinements. First, an authoritative technical taxonomy is adopted to classify keywords into three hierarchical categories—foundational technologies, core technologies, and frontier technologies—providing a standardized basis for systematic hotspot identification and layered analysis. Second, LLMs are incorporated into both data processing and interpretive analysis. At the data level, LLMs enhance consistency in keyword cleaning, abbreviation disambiguation, and cross-lingual normalization. At the interpretive level, LLMs support multi-angle, cross-level analysis by integrating large-scale textual information with urban industrial and policy contexts, thereby improving interpretive depth while reducing reliance on purely experience-based reasoning.

Building on these refinements, this study develops an integrated analytical framework consisting of four components—bibliometric statistics, co-word analysis, network analysis, and LLM-assisted interpretation. Using scholarly publications and patent data from 2016 to 2025 for Wuhan, Hangzhou, Chengdu, and Tianjin across AI, FOC, ICV, and SC, the framework combines bibliometric methods [10], visualization tools such as VOSviewer [11] and LLM-based semantic support, with DeepSeek and ChatGPT used to assist keyword normalization and interpretive synthesis, thereby revealing differentiated patterns in urban high-tech industrial development.

## Literature review

### RIS theory

The RIS theory seeks to explain how different innovation actors within a region interact and collaborate to enable knowledge sharing and collective innovation, thereby promoting regional economic growth and industrial upgrading and providing a theoretical foundation for regional innovation policy. Cooke [12] pioneered the typological framework of RIS by classifying regional innovation systems into three categories—grassroots, networked, and dirigiste (or interventionist)—based on governance structures and knowledge interaction mechanisms. These types correspond respectively to innovation models driven by informal knowledge exchange among local small and medium-sized enterprises, coordinated knowledge networks led by multiple actors from academia, industry, and research institutions, and top-down resource allocation and innovation guidance promoted by governments or large enterprises. Building on this framework, Asheim and Isaksen [13] proposed a threefold classification of territorially embedded systems, regional network systems, and regionalized national innovation systems, thereby enhancing the empirical applicability of RIS typologies. Simmie and Martin [14] introduced the concept of path dependence into RIS research, emphasizing that the evolution of urban and regional innovation systems is strongly shaped by historical structures and existing industrial foundations. Cooke [12] likewise highlighted the dynamic coupling between regional economic structures and innovation behavior, which jointly drives processes of path lock-in and evolutionary transformation. From this perspective, evolutionary economics has become a key theoretical foundation for understanding institutional learning, organizational agglomeration, and knowledge trust mechanisms within RIS. Furthermore, Audretsch and Belitski [15] extended RIS theory from an innovation ecosystem perspective, conceptualizing it as a self-organizing system composed of diverse actors such as firms, universities, and financial institutions, within which regional institutional settings, cultural contexts, and infrastructure jointly form the ecological background supporting value co-creation. In the context of the global knowledge economy, Asheim and Coenen [16] further developed the concept of the “learning region”, arguing that a region’s capacity to absorb, integrate, and transform both codified and tacit knowledge constitutes the core driving force behind the evolution and upgrading of regional innovation systems.

### Differentiated development of urban high-tech industries

Amid the accelerated concentration of top-tier scientific and technological resources in core regions, spatial heterogeneity in regional technological development has become a focal topic in technology management, regional economics, and the geography of innovation. Existing studies typically approach this issue through four lenses: output statistics, textual co-occurrence, collaboration networks, and spatial visualization. In terms of quantitative output, for example, Yaozong Zhu [17] shows via patentometric analysis that the concentration of high-value patents along the eastern seaboard significantly exceeds that of central and western regions, vividly reflecting uneven regional innovation. Yet output gaps alone cannot reveal technological structure and knowledge linkages. To further resolve regional technological features, collaboration-network analysis offers another perspective. Chen [18] uses VOSviewer to construct cross-regional keyword co-occurrence networks, with knowledge maps that display each region’s advantage topics and the strength of their linkages, demonstrating that co-word analysis effectively identifies structural differences in technology. Abbasiharofteh et al. [19] build co-inventor patent networks and find that regions with dense cross-community connections are more likely to generate breakthrough combinations of technologies, indicating that network topology substantially shapes innovation outcomes. After introducing geographic information systems to spatially project collaboration networks, Weinan Gu [20] maps the relationships among regional innovation actors onto geographic coordinates, uncovering a pattern in which innovation activities are highly concentrated within major metropolitan areas while peripheral regions exhibit relatively sparse ties.

### Studies of industrial technology development based on scientometric and patent-analysis methods

With the accelerating pace of knowledge production and technological diffusion, scientometric data have become a key information source for tracing the evolution of industrial technologies and innovation trends [21, 22]. Methodologically, scholars have employed co-citation, co-word, and main-path network analysis to track the accumulation and transformation of technological knowledge. Chi-Yo Huang et al. [23], building on a co-citation network, used main-path analysis to delineate the core chains of knowledge inheritance within a field, thereby revealing the dominant routes of technological evolution. Patent data likewise underscore the value of quantitative analysis: Altuntas et al. [22] devised a technology life-cycle–diffusion model demonstrating that patent indicators can gauge technological maturity and forecast the probability of successful commercialization. Beyond elucidating evolutionary trajectories, scientific publications provide objective yardsticks for evaluating innovation capacity. Zhijing Zhu [24] argues that the volume of Science Citation Index (SCI) papers and patents, the frequency of highly cited works, and grant ratios collectively reflect the originality and dynamism of national or regional innovation. Such quantitative results are widely embedded in macro-policy and strategic planning: Gibson’s review [25] shows that scientometric analysis is increasingly integral to public policy, technology foresight, and research planning; Yaozong Zhu [26], using the geographic distribution of patents and networks of innovation actors, maps the competitive landscape among China, the United States, and Japan in key technologies and proposes recommendations for international collaboration and technological deployment. In sum, scientometrics can serve as a bridge across the “knowledge–technology–policy” chain: it not only traces the main lines of technological change but also supplies evidence for assessing innovation capacity and formulating industrial strategy. In terms of concrete techniques, scientometrics and patentometrics have developed a mature analytical paradigm. Zupic and Čater [27], via tools such as VOSviewer and CiteSpace, construct knowledge maps to reveal thematic evolution and the interaction patterns of innovation actors, thereby enabling a multidimensional, reproducible research workflow.

### Synthesis

Research on differentiated trajectories of urban high-tech development and industrial technological evolution provides critical micro-level support and quantitative instruments for advancing RIS theory. From one perspective, cities exhibit relatively stable differences in key innovation dimensions, including dominant innovation actors (e.g., universities versus firms), industrial orientation (hardware-oriented manufacturing versus software- and platform-based activities), and knowledge transformation pathways (publication-driven versus patent-driven innovation). These dimensions align closely with the core logic underlying classical RIS typologies—namely grassroots, networked, and interventionist systems. By operationalizing and quantifying these variables, cities can be systematically positioned within specific RIS configurations, thereby enabling more structured and reproducible empirical testing and longitudinal tracing of RIS evolution. From another perspective, bibliometric and patent-based approaches offer effective means to uncover inter-regional knowledge flow structures and technological evolution pathways, providing high-dimensional empirical evidence for examining internal mechanisms, heterogeneous development patterns, and path-dependent dynamics within RIS.

Despite notable progress in theoretical development and methodological exploration, important limitations persist in existing research paradigms and analytical depth. On the one hand, traditional approaches—such as surveys, interviews, case studies, and aggregate statistical analysis—are constrained by sample coverage, processing efficiency, and reliance on subjective judgment, limiting their ability to capture the complexity and multi-scale nature of contemporary technological systems. These challenges are further compounded by data-related issues. Bibliometric analyses commonly depend on author-provided keywords and textual metadata; in the absence of standardized technical vocabularies, heterogeneous expressions fragment semantically identical concepts and distort frequency distributions [28]. Likewise, policy-oriented analyses often rely heavily on expert interpretation, making them susceptible to cognitive bias, information asymmetry, and experience-driven reasoning, which may introduce subjectivity into policy conclusions.

On the other hand, much of the literature on regional technological differentiation remains focused on macro-level output comparisons, with insufficient attention to specific technological categories, hierarchical knowledge structures, and collaborative relationships. The connection between quantitative measurement results and policy interpretation is frequently weak, lacking mechanisms that meaningfully integrate regional contexts, industrial structures, and technological trajectories. Consequently, the dynamic interactions among regions, industries, and technologies remain underexplored, limiting the ability of existing studies to inform finely targeted policy interventions and to support evidence-based strategies for regional collaborative innovation.

## Methodology

### Research framework

**Fig 1:**
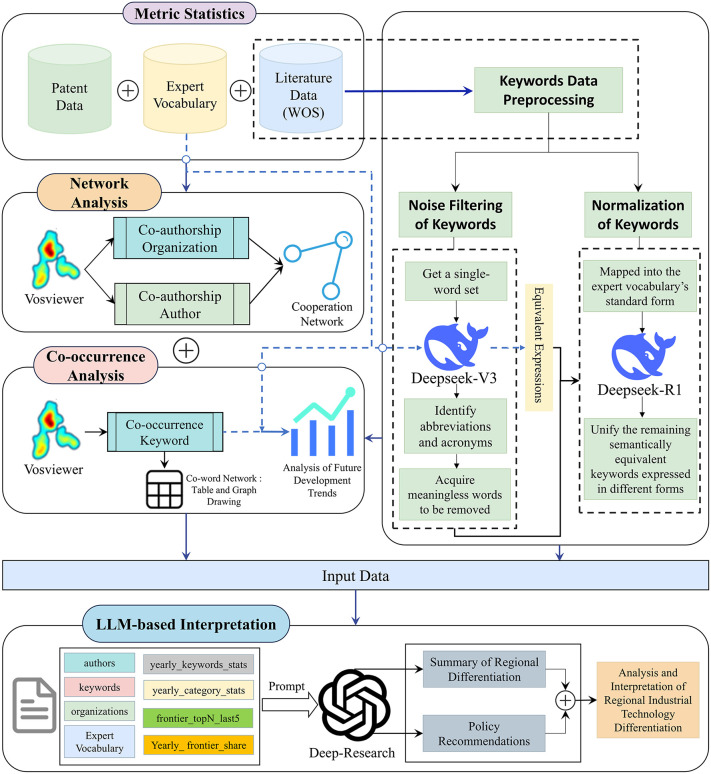
The overall research framework. The system takes bibliometric and patent data as input and outputs visualized networks, statistical indicators, and LLM-based semantic interpretations.

This study adopts a co-occurrence–based analytical approach and leverages VOSviewer to construct and visualize scientific knowledge maps. These maps are combined with an expert-curated technical taxonomy to support semantic interpretation, while LLMs are introduced to enhance the precision of data processing and the interpretive coherence of results. Based on this design, we propose an integrated four-part framework—bibliometric statistics, co-word analysis, network analysis, and LLM-assisted interpretation—to systematically examine differentiated patterns and evolutionary features of urban high-tech development (the overall workflow is summarized in Fig 1).

First, guided by an expert-compiled technical taxonomy encompassing foundational, core, and frontier technologies, we collected scientific publications and patent records from 2016 to 2025 using the WOS and the national patent information platform. These data were cross-tabulated along the two dimensions of region and industry to construct a multi-source dataset suitable for comparative analysis. A central objective at this stage was to delineate technological objects and category boundaries using an authoritative lexicon, thereby ensuring that all subsequent quantitative analyses were conducted within a consistent and comparable analytical frame.

Second, data preprocessing focused on keyword cleansing and standardization to address noise and orthographic inconsistency in the raw data. Individual English tokens were first extracted from the keyword sets, and terms already covered by the expert taxonomy—including plural variants—were retained to avoid the inadvertent removal of core technical concepts. LLM-based semantic recognition was then used to identify and exclude abbreviations or model labels lacking substantive technological meaning (e.g., SVM, ResNet-50), compensating for the limitations of purely rule-based filtering. Remaining tokens were treated as non-informative and removed from the relevant metadata fields. For normalization, equivalence sets were generated for taxonomy entries so that heterogeneous expressions referring to the same concept could be merged into standardized forms. An increasing frequency threshold (starting at two occurrences) was applied to retain keywords until the candidate set was reduced to no more than, and as close as possible to, 200 terms. Equivalence merging was performed only when a concept appeared in at least two distinct lexical forms.

Third, in the modeling and measurement stage, cleansed keywords and author–affiliation information were used to construct a co-word network, an institutional collaboration network, and an author collaboration network in VOSviewer. The co-word network was used to identify topical hotspots and cluster structures, while the collaboration networks illustrated each city’s research partnerships and the organizational structure of core research groups. Building on the three-tier technical taxonomy, multidimensional bibliometric statistics were then conducted. These included an annual analysis of keyword frequencies from 2016 to 2025 to trace the structural evolution of foundational, core, and frontier technologies; an identification of the five most frequent frontier keywords over the most recent five-year period to capture short-term momentum; and an assessment of the annual proportion of frontier technologies relative to all three tiers to measure regional “frontier intensity”. Frontier intensity refers to a quantitative indicator measuring the share of frontier-level technologies among all identified technologies (foundational, core, and frontier) in a given region and time period. However, the taxonomy-based temporal analysis conducted in this stage requires a sufficient density of author keywords that can be reliably mapped onto the three-tier technical classification. Empirically, in the SC domain, keyword–taxonomy alignment was extremely sparse, resulting in limited informational content for robust time-series comparison across regions. For instance, even in Wuhan—the city with the largest publication volume in this domain—only a single frontier-level keyword (*non-volatile memory*) was identified over the past decade. To avoid noise amplification and over-interpretation, SC was therefore not included in this specific analytical step. Subsequent taxonomy-driven temporal analyses focus on AI, ICV, and FOC, where knowledge articulation is richer and alignment with the taxonomy ensures analytical stability and interpretability. Importantly, this stage-specific exclusion reflects data constraints rather than a reassessment of SC’s analytical relevance.

It should be noted that SC was intentionally included in the initial data collection and descriptive analysis as a technologically mature and capital-intensive industry occupying a distinct position in the technology life cycle. Its limited representation in taxonomy-based trend analysis reflects the domain’s structural characteristics—namely, a weaker reliance on diversified scientific keywords and a greater emphasis on process- and firm-specific knowledge—rather than analytical inconsistency.

Finally, LLM-based semantic support via ChatGPT-DeepResearch was used to interpret and contextualize the quantitative results. It linked observed hotspots, evolutionary trajectories, and collaboration patterns to regional industrial and policy contexts, and helped identify representative authors and research contributions. Rather than replacing quantitative evidence, LLM-assisted interpretation synthesized results across analytical layers to inform policy-relevant insights.

Overall, the framework remains firmly grounded in structured data analysis and conventional statistical procedures, while enhanced semantic coherence supports more systematic interpretation and cross-contextual reasoning. This design improves interpretive depth and explanatory clarity without intervening in the underlying statistical logic, measurement process, or reproducibility of the quantitative results.

### Data Source

The dataset comprised three components: an expert-defined technical taxonomy, scientific publications, and patents. The taxonomy was developed with intellectual support from the Wuhan Science and Technology Strategy Research Institute, jointly established by the Wuhan Municipal Science and Technology Bureau and Wuhan University of Technology. The institute has established extensive collaborative relationships with eleven high-level laboratories across Hubei Province, including the National Key Laboratory of Optical Communication Technology and Networks, the Hubei Optics Valley Laboratory, and the Wuhan Institute of AI. Drawing upon domain reports produced through these collaborations, industry experts distilled the taxonomy from sectoral analyses and policy documents and organized it into a three-tier scheme—foundational, core, and frontier technologies—serving as the baseline knowledge base throughout the analytical workflow. The technical taxonomy used in this study is presented in Table 1, and the complete expert-defined taxonomy is provided in S1 File. The taxonomy functioned in two principal ways. First, it served as a standardized keyword set against which keywords extracted from the WOS were systematically matched and classified, thereby reducing subjectivity and harmonizing definitions. Second, it provided an operable hierarchical labeling mechanism that supported evolutionary analysis and cross-regional comparison. Under this mechanism, annual frequencies of the three technology tiers were compared to trace overall technological trajectories; rolling counts of frontier-level terms were conducted to identify recent hotspots; and the annual share of frontier technologies within the total of all three tiers was calculated to gauge each region’s degree of frontier intensity. In this way, the data foundation was both authoritative and reproducible, supplying quantifiable support for revealing regional differences and the cadence of technological evolution.

**Table 1:** Distribution of the Authoritative Technical Taxonomy Across Four Industries.

Industry	Foundational Technology	Core Technology	Frontier Technology
AI	34	25	15
SC	11	7	10
FOC	59	32	25
ICV	20	23	26

Publication data were obtained from the WOS Core Collection and restricted to SCI and SSCI journals for the period 2016–2025 to ensure scholarly authority and international comparability. The retrieval strategy prioritized WOS subject categories (WC); where WC coverage was insufficient, topic searches (TS) were constructed around four domains—AI, FOC, ICV, and SC—with review literature and the expert taxonomy used to filter keywords with unambiguous technological referents. For geographic filtering, the “author affiliation address” field was limited to Wuhan, Chengdu, Hangzhou, and Tianjin, yielding a total of 39,205 publication records across the four cities and four domains, including bibliographic data, author keywords, and author and affiliation metadata.

Patent data were drawn from the National Key Industries Patent Information Service Platform and retrieved using a two-stage strategy. In the first stage, domain-specific technical keyword queries were constructed and applied within the corresponding first-level industry categories of the patent retrieval system. In the second stage, the search results were constrained by the “address” field to restrict patent applicants to the target regions. After screening, a total of 9,945 patent records meeting the criteria were retained for the four industries across the four cities.

### LLM selection and roles

During data processing, LLMs were introduced as auxiliary tools to support keyword cleaning, normalization, and interpretive analysis. It should be emphasized that this study does not treat different LLMs as independent analytical instruments for comparison. Instead, they are employed as language-processing interfaces assigned to specific tasks at different stages of the workflow. Their use does not affect subsequent bibliometric analysis or statistical modeling, nor does it introduce differences attributable to model choice. Because the substantive conclusions are derived from the bibliometric patterns, network structures, and the cleaned keyword system under fixed analytical procedures, rather than from any comparison between LLMs, this task-specific division of labor does not alter the study’s core findings.

For reasons of general applicability and accessibility, DeepSeek—a widely used AI model in China—was selected for keyword denoising and normalization. In the keyword-processing stage, DeepSeek (deepseek-chat) was accessed via an API to perform tasks with relatively high structural constraints and explicit rule definitions. These tasks included identifying and removing abbreviations or shorthand expressions lacking clear technological content, as well as assisting in the generation of equivalent expressions for terms in the expert-defined technical taxonomy. This API-based approach is well suited for batch processing and repetitive single-item judgments, ensuring consistency and reproducibility in keyword cleaning and normalization. By contrast, the construction of equivalence-mapping tables for bibliographic keywords was conducted through an interactive web-based interface. This task requires integrated semantic judgment across multiple alternative expressions referring to the same concept and the generation of mappings that remain interpretable to human readers, making it unsuitable for simple batch API calls.

In addition, the ChatGPT-DeepResearch function was employed to support semantic interpretation and analytical writing. Deep Research is a multi-step analytical agent integrated into ChatGPT that performs staged information retrieval and synthesis by combining model-internal knowledge with cross-document and online information sources, rather than relying solely on fixed training data. When additional context or clarification was required, Deep Research retrieved relevant information from diverse sources, including news reports, academic literature, and statistical data, and synthesized insights through planned multi-step reasoning processes. Public benchmark evaluations indicate that this function exhibits strong performance in complex reasoning tasks. For example, in the Humanity’s Last Exam benchmark released in February 2025, Deep Research achieved an accuracy of 26.6%, outperforming the second-ranked OpenAI o3-mini (High) model, which scored 13.0%. Compared with conventional LLM interactions that yield brief responses, Deep Research typically produces structured long-form outputs organized by sections and supplemented with traceable source references. Accordingly, it is well suited for extending bibliometric findings with semantic interpretation and for providing multi-perspective analytical support in discussions of urban high-tech industrial differentiation and policy implications.

### Data processing

The keywords used in this study were batch-exported by VOSviewer from WOS records, including both author-provided keywords and indexed keywords. To reduce contingency and noise, unless otherwise specified, the analysis was restricted to keywords with an occurrence frequency of at least two, thereby ensuring a minimum level of scholarly recognition and representativeness. On this basis, keyword denoising and normalization were performed.

For denoising, close inspection of the raw data showed that non-informative or non-technical terms were predominantly single English tokens (e.g., model). We first protected terms already included in the expert taxonomy (and their plural forms), then used the semantic recognition capability of LLMs (prompt available in S2 File) to identify and remove abbreviations or model labels lacking substantive technical content (e.g., *SVM*, *ResNet-50*). Remaining single-word tokens were uniformly deleted (synchronously in the ID and DE fields), yielding a cleaner candidate set.

For normalization, to address frequency fragmentation caused by synonymous variants, we first had the LLMs (prompt available in S3 File) pre-generate equivalence sets for taxonomy entries, covering symbol splitting, hyphen/space alternation, singular–plural conversion, US/UK spelling variants, and acronym/full-form parenthetical combinations. We then applied an increasing threshold *T* (starting at 2) to filter high-frequency candidates, capping the set at no more than—and as close as possible to—200 entries. Next, we aggregated synonyms using a “first-occurrence priority” rule to fix the canonical label and produce a JSON-structured equivalence map (prompt available in S4 File). This “rules + LLMs” hybrid strategy balanced standardization and variant coverage, providing a reproducible, low-noise keyword basis for subsequent analyses.

Table 2 compares changes in high-frequency keyword rankings before and after denoising and normalization. The results show that, prior to processing, the most frequent keywords were dominated by generalized methodological terms such as model, algorithm, and system. Although these terms appeared frequently, they carry weak technological specificity and are of limited value for characterizing concrete technological pathways. If such raw keyword sets were directly used for VOSviewer-based co-occurrence analysis, the resulting network structure would be dominated by methodological noise, reducing interpretability and potentially obscuring keywords with genuine technological significance.

**Table 2:** Comparison between traditional keyword statistics and LLM-assisted normalization.

Keyword(*Origin*)	occurrences	TLS	Keyword(*Now*)	occurrences	TLS
model	551	3707	deep learning	488	1464
optimization	522	3860	neural-networks	433	1787
algorithm	499	3070	feature extraction	293	1554
deep learning	486	2371	cnn	284	912
classification	344	2111	genetic algorithm	229	923
network	312	1647	differential evolution	222	891
design	277	2076	task analysis	222	1282
feature extraction	265	2381	particle swarm optimization	190	819
genetic algorithm	229	1467	attention mechanism	157	434
systems	224	1879	multi-objective optimization	157	635

**Note:** *TLS* (Total Link Strength) refers to the summed strength of an item’s connections with all other items in the network. *Keyword (Origin)* refers to the original keyword form retrieved from WOS. *Keyword (Now)* refers to the cleaned and standardized keyword form after LLM-assisted denoising, normalization, and synonym merging.

In addition, the coexistence of synonymous variants and formal alternatives substantially fragmented keyword frequencies, leading to an underestimation of important technical concepts in the original rankings. For example, in the unnormalized list, neural-networks, neural networks, and neural-network appeared 152, 111, and 110 times, respectively, each ranking outside the top ten when counted separately. After equivalence merging, however, the aggregated frequency of this concept increased markedly, elevating its rank to second overall. This comparison demonstrates that LLM-assisted denoising and normalization not only reduce interference from irrelevant high-frequency terms but also effectively restore the structural importance of key technological concepts in co-occurrence analysis.

By contrast, after automated processing using the “rules + LLMs” hybrid strategy, the overall keyword ranking structure changed substantially. On the one hand, generalized terms lacking clear technological content were significantly down-ranked or removed. On the other hand, concrete technical units (e.g., CNN, genetic algorithm) and functional technological concepts (e.g., feature extraction, attention mechanism) occupied more central positions in the high-frequency list. These results indicate that the introduction of LLMs does not replace statistical analysis; rather, it enhances the semantic discriminability and structural consistency of keywords, thereby providing a more reliable technological-semantic foundation for subsequent co-word modeling and network analysis.

### Analytical methods

#### City-level comparison with VOSviewer.

We first used VOSviewer to model and visualize co-occurrence among keywords, institutions, and authors, thereby revealing research themes, collaboration patterns, and technological trajectories.

In the keyword layer, we selected the Co-occurrence analysis type and enabled All keywords (author keywords plus Keywords Plus) to ensure comprehensive semantic coverage. To avoid excessive network density while preserving clear salience of principal hotspots, we capped the number of keywords at 200 and sorted them by occurrences, highlighting high-frequency and more representative topics. The resulting co-word network effectively disclosed cluster structures across technological directions and their interlinkages, providing a thematic basis for trend analysis.

In the collaboration layer, we selected the Co-authorship analysis type and constructed separate networks for Authors and Organizations. By tuning the Minimum number of documents of an author parameter, we kept visible nodes to approximately 200 and sorted them by document count to foreground more active scholars and institutions. Comparing the author and institutional networks respectively characterized the cooperation patterns of core researchers as well as the organizational-level coordination modes and cross-regional ties among research actors.

#### Differentiated Analysis and Interpretation.

**Fig 2:**
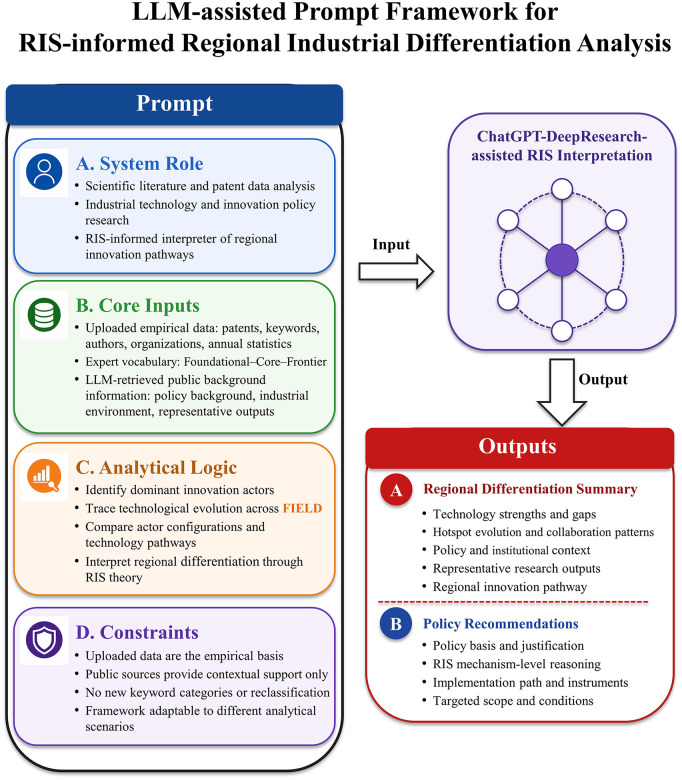
ChatGPT Prompt Engineering.

Building on the VOSviewer-based network modeling of keywords, authors, and institutions, as well as the quantitative statistics of the three categories of technological keywords derived from the technical taxonomy, this study further employs the Deep Research function within ChatGPT to conduct differentiated analysis and integrative interpretation. Fig 2 illustrates the prompt-engineering workflow used for this stage, and the complete prompt is provided in S5 File. This stage is grounded in the bibliometric results and focuses on identifying and explaining structural differences across cities in terms of technological configuration, research hotspots, and collaboration structures through systematic synthesis and comparison.

Specifically, the analysis examines the performance of Wuhan, Hangzhou, Chengdu, and Tianjin across AI, FOC, ICV, and SC, and organizes the findings in a city-specific and industry-specific structure. The resulting conclusions are developed along four dimensions: first, summarizing the relative strengths and weaknesses of each city in technological layout, hotspot evolution, and collaboration patterns; second, identifying logical linkages and structural differences through cross-city comparison; third, discussing how regional industrial policies and support environments influence technological development trajectories and the growth paths of research actors; and fourth, selecting representative authors or institutional research outputs as illustrative cases to substantiate the academic impact and practical relevance of regional scientific production.

On this basis, the study derived policy implications for each of the four cities through a structured, city-by-city interpretive process. Policy discussions were organized in a “city-specific subheading plus narrative” format and were explicitly grounded in the Regional Differentiation Summary for each city. Rather than presenting standalone recommendations, each policy discussion was developed as a reasoned extension of the empirically observed patterns and RIS-based interpretations, linking identified structural constraints and opportunities—such as imbalances in innovation actor configurations, uneven technological evolution, or weak collaboration mechanisms—to corresponding policy leverage points. By maintaining this explicit linkage between empirical analysis, theoretical interpretation, and policy reasoning, the study established a coherent analytical-to-policy logic that ensures internal consistency between findings and policy implications.

## Results and analysis

### Temporal configuration of scientific publications and patents

To systematically characterize the relationship between scientific knowledge production and technological application across cities in high-tech industries, this study integrates publication and patent data over the period 2016–2025 from a temporal perspective. Publication output reflects the accumulation of scientific knowledge in specific technological fields, whereas patent activity represents the advancement of technological application and industrialization. Examining the two within a unified temporal framework facilitates the identification of how different cities configure the timing and pace of research expansion and technological application within the same industry, as well as the heterogeneity of these configurations.

**Fig 3:**
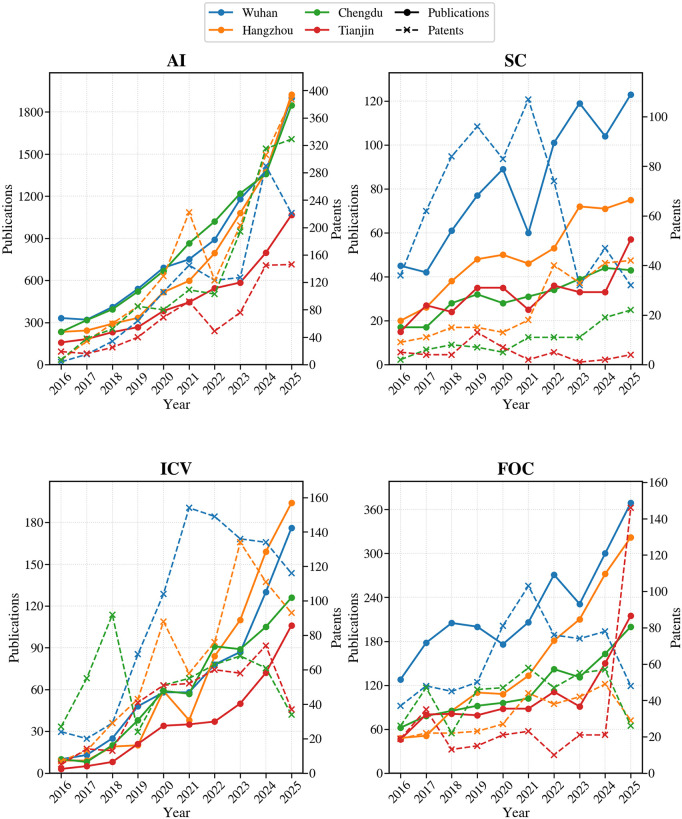
Temporal evolution of publication and patent outputs across cities and industries. Literature data were obtained from the WOS Core Collection and Patent data were drawn from the National Key Industries Patent Information Service Platform.

As shown in Fig 3, most industries exhibit a sustained increase in publication output during the study period. In particular, after 2019, research output in most cities entered a phase of accelerated growth. In contrast, patent activity shows markedly different temporal response patterns across cities. Rather than following publication growth in a synchronized manner, the relationship between research expansion and technological application displays diverse temporal configurations, indicating that the two processes are not mechanically aligned.

In the AI domain, all four cities experienced substantial growth in scientific publications, but the temporal relationship between publications and patents differed markedly across cities. In Hangzhou, patent activity remained relatively synchronized with the expansion of scientific output, with patent growth broadly keeping pace with increasing publication volume. By contrast, in Wuhan and Chengdu, patent growth generally lagged behind publication expansion, suggesting a temporal pattern in which scientific production preceded technological application. Tianjin, while smaller in overall scale, showed more stage-specific fluctuations. Taken together, these patterns suggest that cities differ considerably in how research outputs and technological application are temporally aligned in frontier technologies.

A different pattern appeared in the SC domain, where the publication–patent relationship showed characteristics distinct from those of the other industries. Publication output remained relatively limited and grew at a moderate pace, whereas patent activity in some cities was more concentrated in specific periods. Notably, Wuhan repeatedly recorded patent counts exceeding publication output during the study period, suggesting a stronger orientation toward technological application than academic publication. Other cities, by contrast, showed more limited patent growth and weaker temporal coupling between publications and patents. This pattern suggests that, in a technologically mature and capital-intensive industry such as storage chips, inter-city differences in the balance between research input and technological application may be more pronounced.

The ICV domain showed broadly similar trajectories in publication growth across cities, but patent activity diverged more clearly in both timing and intensity. Wuhan and Chengdu displayed relatively strong patent activity in the earlier stages of the study period, followed by gradual stabilization, whereas Hangzhou showed more pronounced acceleration in the middle and later stages. Tianjin, despite its smaller overall scale, also concentrated its patent growth peaks in the later period. These differences point to varying rhythms of technological exploration and industrialization across cities and suggest that the development paths of growth-stage industries are not temporally synchronized.

As a more mature industry, FOC showed a relatively stronger coupling between publications and patents over time, although structural differences across cities remained evident. Wuhan consistently maintained high levels of both scientific output and patent activity throughout the study period, suggesting a relatively stable relationship between research and application. Other cities showed varying degrees of divergence in the scale and volatility of patent growth, indicating heterogeneous temporal configurations.

**Fig 4:**
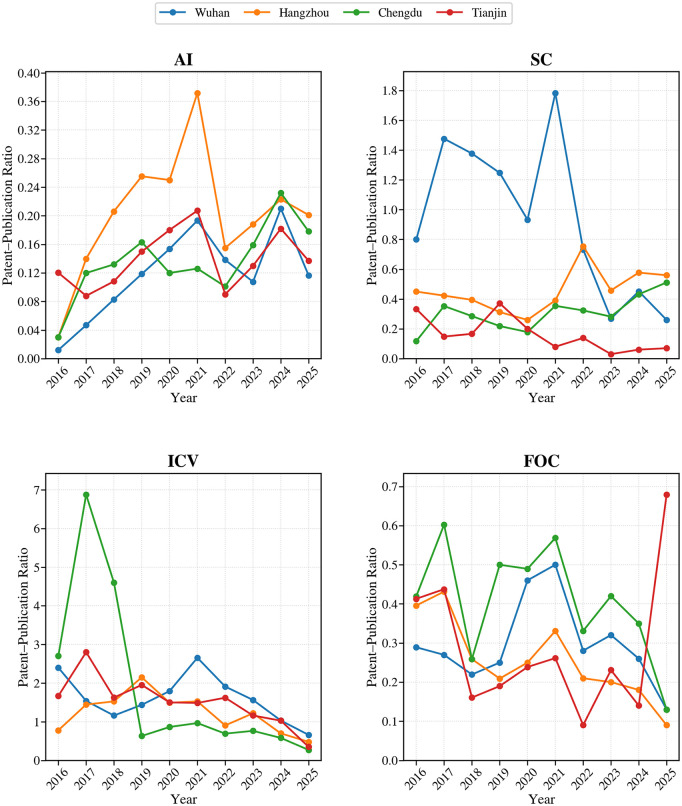
Temporal patterns of patent–publication ratios across cities and industries.

Further evidence comes from the annual evolution of the patent-to-publication configuration ratio. As shown in Fig 4, emerging and growth-stage industries such as AI and ICV displayed greater temporal volatility in this ratio, with cities differing markedly in the timing between research expansion and technological application. By contrast, in more mature industries such as FOC and SC, the ratio remained relatively stable overall, although structural differences across cities persisted. Notably, although publication output in most industries rose simultaneously across cities in 2025, the timing and magnitude of patent responses still varied substantially, suggesting a continuing non-synchronous relationship between research expansion and technological application.

Overall, inter-city differences in high-tech development are reflected not only in the scale of scientific or patent outputs, but more importantly in how these outputs are temporally configured and evolve over time. Such temporal configuration differences provide a critical perspective for understanding differentiated trajectories of urban high-tech development.

### City-level research hotspots in high-tech industries

**Fig 5:**
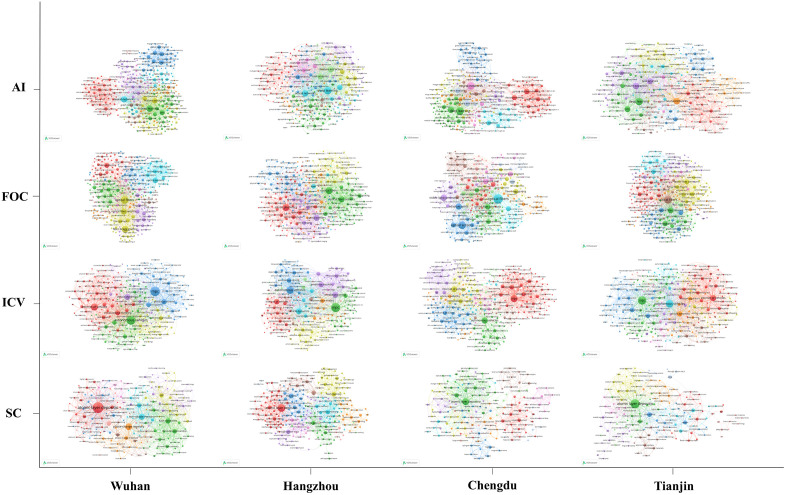
Co-occurrence network of author keywords. The figure covers four cities (Wuhan, Hangzhou, Chengdu, and Tianjin) and four technological domains (AI, FOC, ICV, and SC), illustrating their respective research hotspots and interconnections.

By analyzing co-occurring author keywords, we identified city- and industry-specific research hotspots, yielding a clearer view of each region’s research orientations and technological trends. The ten most frequently occurring keywords are listed in Table 3, and the co-occurrence networks of author keywords across the four cities and four technological domains are illustrated in Fig 5.

**Table 3:** Author keywords.

address	industry	author keywords
Wuhan	AI	deep learning, neural-networks, feature extraction, cnn, genetic algorithm, differential evolution, task analysis, particle swarm optimization, attention mechanism, multi-objective optimization
	SC	atomic layer deposition, phase-change memory, non-volatile memory, flash memories, thin-films, random access memory, three-dimensional displays, ssd, rram, phase change materials
	ICV	mpc, autonomous vehicles, internet of vehicles, vehicle dynamics, predictive control, computational modeling, predictive models, feature extraction, mathematical models, autonomous driving
	FOC	optical fibers, fiber bragg grating, optical fiber sensors, optical sensors, silicon photonics, fiber gratings, optical fiber communication, temperature sensors, refractive-index, optical imaging
Hangzhou	AI	deep learning, neural networks, feature extraction, task analysis, cnn, graph neural networks, transformer, data models, machine learning, computational modeling
	SC	atomic layer deposition, thin-films, flash memory, nonvolatile memory, phase change memory, through-silicon via (tsv), logic gates, lithium-ion batteries, electrical-properties, carbon nanotubes
	ICV	mpc, autonomous vehicles, predictive control, vehicle dynamics, internet of vehicles, predictive models, autonomous driving, voltage control, mathematical models, finite control-set model predictive control (fcs-mpc)
	FOC	optical fibers, silicon photonics, optical fiber sensors, optical sensors, laser, fiber bragg grating, optical switches, mach-zehnder interferometer, optical waveguides, optical imaging
Chengdu	AI	deep learning, neural-networks, cnn, feature extraction, group decision-making, task analysis, decision-making, feature selection, aggregation operator, attention mechanism
	SC	atomic layer deposition, thin-films, object detection, electrochemical performance, neural networks, feature extraction, logic gates, resistive switching, deep learning, lithium-ion batteries
	ICV	mpc, autonomous vehicles, internet of vehicles, predictive control, voltage control, computational modeling, resource allocation, predictive models, internet of things, mathematical model
	FOC	optical fibers, optical sensors, visible light communication, adaptive optics, free-space optical communication, optical fiber communication, optical imaging, fiber bragg grating, orbital angular-momentum, optical fiber sensors
Tianjin	AI	deep learning, neural-networks, feature extraction, task analysis, cnn, object detection, transformer, feature selection, particle swarm optimization, attention mechanism
	SC	atomic layer deposition, thin-films, reram, al_2_o_3_, cmp, nonvolatile memory, flash memory, atomic layer epitaxy, nand flash memory, lithium-ion batteries
	ICV	mpc, autonomous vehicles, internet of vehicles, vehicle dynamics, predictive control, predictive models, heuristic algorithms, deep reinforcement learning, real-time systems, task analysis
	FOC	optical fibers, optical fiber sensors, fiber bragg grating, optical fiber communication, refractive-index, photonic crystal fiber, orbital angular momentum, optical sensors, mach-zehnder interferometer, silicon photonics

**Note:** Keyword rankings are sorted in descending order.

Across AI, all four cities converged on core algorithmic topics such as “deep learning” and “neural network”, indicating sustained attention to foundational methods. Their emphases nonetheless differed: Wuhan gave greater prominence to swarm-intelligence approaches such as “particle swarm optimization” and “differential evolution”; Hangzhou focused more on emerging architectures, especially “graph neural network” and “Transformer”; Chengdu highlighted “group decision-making” and “aggregation operator”, suggesting a distinctive concentration in multi-agent decision-making; and Tianjin emphasized “reinforcement learning” and “deep reinforcement learning”, pointing to an orientation toward dynamic decision-making and adaptive learning.

In SC, “atomic layer deposition” appeared as a shared core across cities, underscoring its importance in semiconductor manufacturing. Wuhan placed more emphasis on “phase-change memory” and “non-volatile memory”; Hangzhou on “through-silicon via” and “electrical characteristics”; Chengdu on “resistive switching” and “logic gate”; and Tianjin on “ReRAM” and “NAND flash”, indicating differentiated attention to storage, packaging, device-level fundamentals, and application-oriented memory technologies.

A similar pattern appeared in ICV, where “model predictive control” and “autonomous driving” were common hotspots. Wuhan emphasized “autonomous vehicles” and “internet of vehicles (IoV)”; Hangzhou highlighted “predictive model” and “voltage control”; Chengdu focused on “resource allocation” and “computational modeling”; and Tianjin gave more prominence to “real-time system” and “deep reinforcement learning”, reflecting different orientations in connectivity, system modeling, optimization, and responsive control.

In FOC, all four cities shared attention to “fiber-optic sensor” and “optical sensor”, highlighting sensing as a key technological focus. Wuhan emphasized “fiber Bragg grating” and “refractive index”; Hangzhou concentrated on the “Mach–Zehnder interferometer” and “optical switch”; Chengdu highlighted “free-space optical communication” and “visible light communication”; and Tianjin focused on “photonic crystal fiber” and “fiber polarization”.

### Future Development Trends in Urban High-Tech Industries

**Fig 6:**
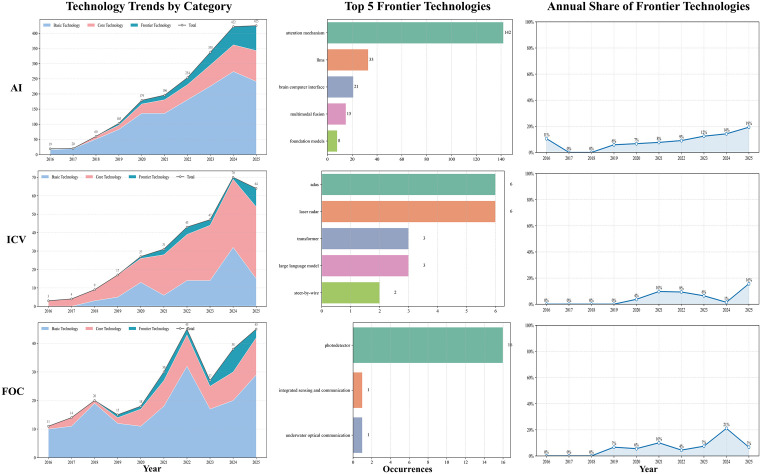
Technological trend chart of the three technology categories in Wuhan.

**Fig 7:**
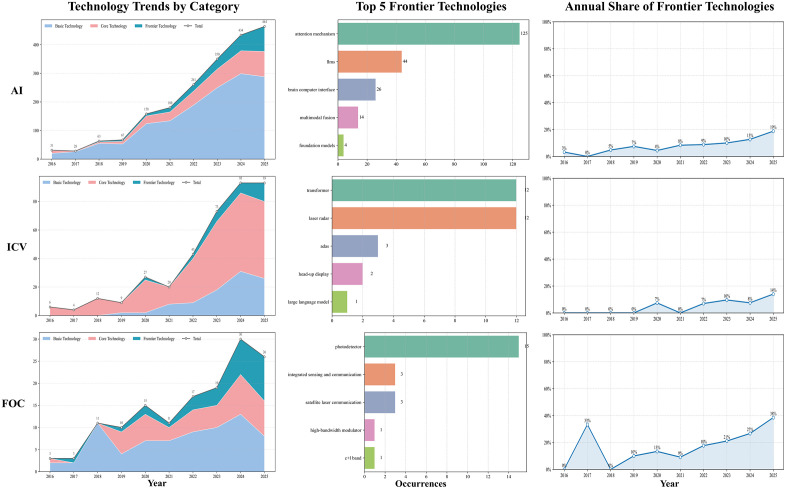
Technological trend chart of the three technology categories in Hangzhou.

**Fig 8:**
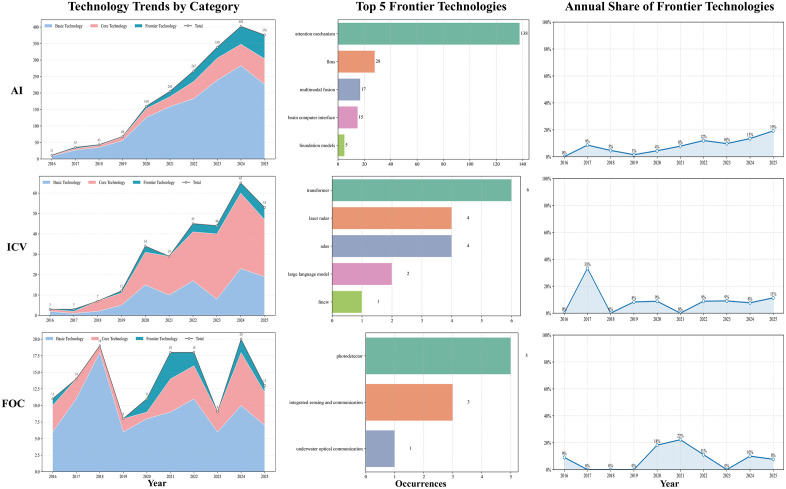
Technological trend chart of the three technology categories in Chengdu.

**Fig 9:**
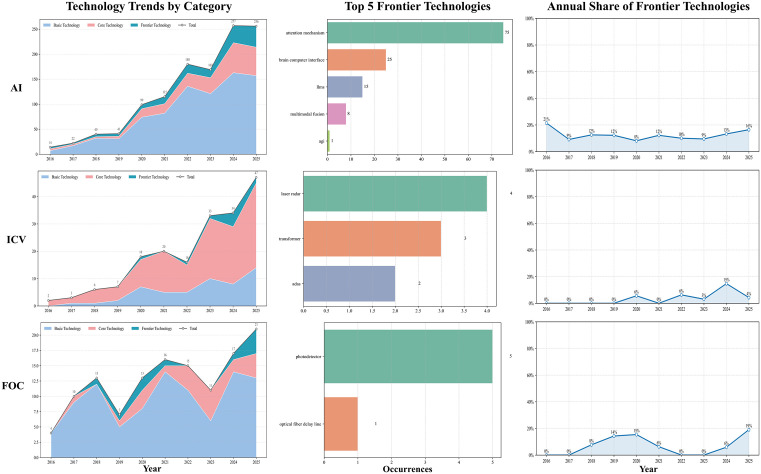
Technological trend chart of the three technology categories in Tianjin.

By tallying author keywords mapped to the three-tier technical taxonomy and analyzing their temporal trajectories, we delineated regional technological pathways and potential inflection points across industries, thereby providing objective, quantitative support for trend assessment. The temporal evolution of AI, ICV, and FOC across the four regions is illustrated in Figs 6–9.

For AI, all four cities had broadly shifted from a convolution-centered paradigm toward attention mechanisms and LLMs, with frontier shares rising to around 19%, except Tianjin, which remained slightly lower (16%). Differences were mainly visible in frontier composition and application orientation. Hangzhou moved earlier and more deeply into LLMs and multimodality, with greater emphasis on Transformer engineering and scenario-based deployment, and ranked highest in both the number and share of frontier keywords. Wuhan followed a dual trajectory of foundational and frontier development, with fewer LLM-related terms and relatively greater emphasis on theoretical modeling and visual perception. Chengdu showed frequent attention-mechanism terms and, although it lagged behind Hangzhou in large-model and cross-modal engineering, displayed a more concentrated frontier profile in decision-making and representation optimization. Tianjin remained smaller in scale but explored differentiated directions such as brain–computer interfaces and artificial general intelligence (AGI).

In the ICV domain, all four cities advanced within an integrated perception–decision framework, although the overall frontier level remained relatively low (all ≤ 14% in 2025). Their pathways differed in pace and emphasis. Hangzhou integrated frontier algorithms such as Transformer more extensively into autonomous-driving perception and decision modules and combined this with open-road testing; frontier terms such as “LiDAR” and “Transformer” appeared more frequently than in peer cities. Wuhan focused on advanced driver-assistance systems, IoV security, and LiDAR, with frontier development emerging later and showing a stronger emphasis on safety and verifiability. Chengdu emphasized communication–control integration, including on-board communications and edge-computing scheduling, and showed activity in verifiable modules such as steer-by-wire, while maintaining active international collaboration. Tianjin progressed mainly through standards and testing systems, with more limited incorporation of frontier algorithms, suggesting a steadier engineering-oriented pathway.

In FOC, all four cities shared a common trend toward advances in photodetector technologies, but their development routes differed. Wuhan maintained the strongest advantages in both scale and breadth, with a wider frontier portfolio that also extended into inter-satellite communication. Hangzhou followed a more focused strategy, concentrating on satellite laser communication and quantum random numbers, and recorded the fastest rise in frontier share. Chengdu remained comparatively strong in core-technology areas such as transmission and network architecture but showed less activity in frontier fields, leaving its frontier level below Wuhan and Hangzhou. Tianjin appeared more application-oriented, focusing on fiber-optic sensing and optical-fiber delay-line scenarios, with a limited frontier keyword count and a more gradual engineering-led trajectory.

### Analysis of Collaboration Patterns in Urban High-Tech Industries

In the evolution of regional industrial technology, research collaboration serves as a key mechanism for catalyzing knowledge diffusion and concentrating innovation, exerting far-reaching effects on capability trajectories and regional competitive advantage. To systematically uncover the collaboration structures and network properties of cities within high-tech industries, we conducted analysis at two levels: the organizational level—using organizational co-occurrence to examine inter-institutional coordination and regional collaboration intensity—and the individual level—using author co-occurrence to depict cooperation structures and the characteristics of core research groups.

### Organizational Co-Occurrence Analysis

From a RIS perspective, organizational collaboration structures reflect the configuration of dominant R&D actors and the institutional logic through which knowledge is produced and mobilized. By examining organizational co-occurrence networks, this analysis identifies which actors—universities, research institutes, enterprises, or state-affiliated organizations—occupy central positions across industries and cities, thereby revealing distinct RIS configurations in practice.

**Fig 10:**
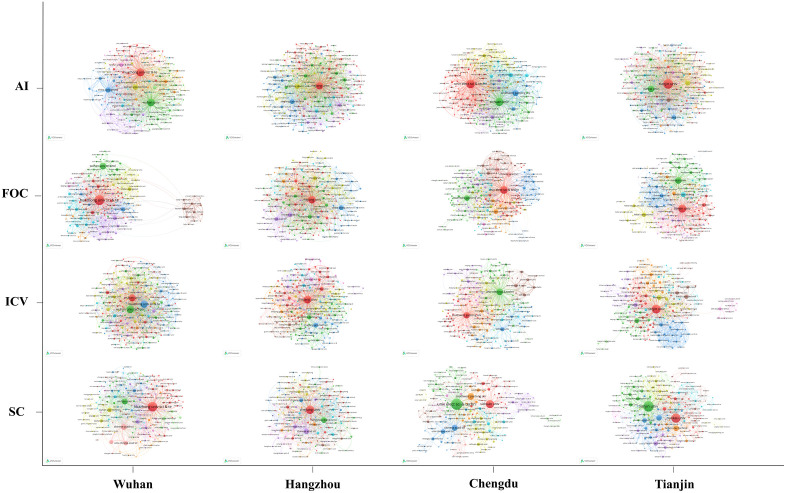
Co-occurrence networks of industrial collaboration organizations across four cities and four technological domains.

Analyzing the co-occurrence networks of collaborating organizations thus clarifies each city’s principal partners, tie strengths, and overall collaboration structure. The top five organizations by document count are reported in Table 4, and Fig 10 visualizes the organizational co-occurrence networks across the four cities and technological domains.

**Table 4:** Collaborating organizations.

address	industry	organizations
Wuhan	AI	huazhong univ sci & technol, wuhan univ, wuhan univ technol, china univ geosci, wuhan univ sci & technol
	SC	huazhong univ sci & technol, wuhan univ, chinese acad sci, univ chinese acad sci, wuhan univ technol
	ICV	wuhan univ technol, huazhong univ sci & technol, wuhan univ, hubei univ technol, china univ geosci
	FOC	huazhong univ sci & technol, wuhan univ technol, wuhan univ, chinese acad sci, china univ geosci
Hangzhou	AI	zhejiang univ, hangzhou dianzi univ, zhejiang univ technol, zhejiang lab, alibaba grp
	SC	zhejiang univ, hangzhou dianzi univ, chinese acad sci, zhejiang univ technol, univ chinese acad sci
	ICV	zhejiang univ, zhejiang univ technol, hangzhou dianzi univ, beihang univ, tsinghua univ
	FOC	zhejiang univ, china jiliang univ, chinese acad sci, hangzhou dianzi univ, univ chinese acad sci
Chengdu	AI	univ elect sci & technol china, sichuan univ, southwest jiaotong univ, southwestern univ finance & econ, chengdu univ informat technol
	SC	univ elect sci & technol china, sichuan univ, southwest jiaotong univ, chinese acad sci, univ chinese acad sci
	ICV	univ elect sci & technol china, southwest jiaotong univ, sichuan univ, univ elect sci & technol china uestc, xihua univ
	FOC	univ elect sci & technol china, southwest jiaotong univ, chinese acad sci, sichuan univ, univ chinese acad sci
Tianjin	AI	tianjin univ, nankai univ, hebei univ technol, tianjin univ technol, tiangong univ
	SC	tianjin univ, nankai univ, chinese acad sci, hebei univ technol, tianjin univ technol
	ICV	tianjin univ, hebei univ technol, nankai univ, tianjin univ technol, tiangong univ
	FOC	tianjin univ, nankai univ, tianjin univ technol, hebei univ technol, chinese acad sci

**Note:** Organization rankings are sorted in descending order.

By analyzing the co-occurrence network of collaborating organizations, Wuhan relied heavily on leading local universities across the four industries. Wuhan University and Huazhong University of Science and Technology occupied core positions in AI and SC, while Wuhan University of Technology and China University of Geosciences sustained influence in ICV and FOC. This pattern indicated a collaboration model centered on local flagships, in which “inter-university alliances + research-institute cooperation” drove industrial progress. As shown in Wuhan’s organizational co-occurrence map (Fig 10), Huazhong University of Science and Technology and the Chinese Academy of Sciences (UCAS) maintained close ties with Hong Kong Polytechnic University, Nanyang Technological University, the University of Sydney, and other international universities, and the overall network exhibited pronounced clustering. Different colors denoted relatively stable collaboration communities: in AI, a green cluster was centered on Wuhan University, linking the central-China region and selected international universities; a red cluster centered on Huazhong University of Science and Technology, connecting key national universities and research institutes; and the blue and orange clusters included UCAS and China University of Geosciences, engaging in cross-disciplinary and cross-domain collaboration. This clustered structure reflected both division of labor and complementarity among institutions, as well as diversity and differentiation of collaboration themes. Overall, Wuhan exhibits a university- and research-institute-led RIS, in which local flagship universities function as stable knowledge anchors, while national institutes and international partners extend the system’s scientific depth and global connectivity.

In Hangzhou, collaboration was led by Zhejiang University and spanned AI, SC, ICV, and FOC, forming a cross-industry, broad-participation pattern. Hangzhou Dianzi University, Zhejiang University of Technology, and China Jiliang University provided strong support in FOC and ICV and maintained stable ties with top domestic research bodies. Some teams actively expanded international exchanges, facilitating the wide circulation and translation of research results. As Fig 10 further shows, in AI, Zhejiang University and Hangzhou Dianzi University not only occupied core positions but also maintained close cooperation with enterprises such as Alibaba Group and Ant Group; collaborations with Nanyang Technological University and the University of Sydney enhanced openness and frontier orientation. In FOC, Zhejiang University and Zhejiang Lab led research while linking Westlake University, the Chinese Academy of Sciences, and Hong Kong Polytechnic University, forming a dual track of “established research bodies + emerging research forces.” In ICV, local universities were tightly connected and maintained strong ties with Tsinghua University, Beihang University, and other key national institutions, highlighting an engineering orientation and industrialization potential. Collaboration in SC was relatively localized while maintaining exchanges with select international partners, reflecting steadiness alongside outward extension. Hangzhou’s collaboration structure reflects an industry-integrated RIS, where leading universities remain central knowledge producers, while major platform firms are deeply embedded in research networks, enabling strong market feedback and frontier-oriented knowledge translation.

In Chengdu, the organizational co-occurrence network was anchored by the University of Electronic Science and Technology of China and Sichuan University, which wielded strong influence in AI, SC, and FOC; Southwest Jiaotong University (SWJTU)—the “cradle of China’s railway engineers”—played a central role in ICV. Participation by Southwestern University of Finance and Economics, Chengdu University of Information Technology, and other institutions gave Chengdu’s network a multi-layered, composite character. This model strengthened the city’s research base in information, communications, and intelligent technologies while enabling cross-disciplinary innovation. Across the four networks, the AI graph showed three primary nodes bridged by economics/management and information-science universities, indicating interdisciplinary synergy. In FOC, UCAS was deeply embedded and formed clusters with Swinburne University of Technology, ITMO University, and other international partners—an open pattern of “national capacity + international channels.” In ICV, SWJTU occupied the transportation core and, together with local strengths, formed joint linkages of “mechanism-based control × data-driven methods.” In SC, UCAS and enterprise nodes such as Huawei Technologies were directly embedded, underscoring “university–institute–enterprise” collaboration. Overall, Chengdu’s pattern could be summarized as “aggregation around local flagships—reinforcement by national institutes—extension to international partners—enterprise nodes embedded”, ensuring both scale and depth while providing a robust, open basis for cross-disciplinary collaboration and technology transfer. Chengdu represents an academically strong but weakly coupled RIS, characterized by solid university-led research capacity, partial integration of national institutes, and a growing yet still fragmented linkage to industrial actors.

In Tianjin, research collaboration was primarily led by Tianjin University and Nankai University, which together covered core directions across the four industries. The co-occurrence maps showed stable links with national-level bodies such as UCAS and Beihang University, alongside partnerships with Hong Kong Polytechnic University, the University of Oxford, the University of Tokyo, and other international universities—evidence of cross-regional and international orientation. In ICV, Tianjin University maintained close ties with industry institutions such as the China Automotive Technology and Research Center, reflecting a strong application focus. In FOC and SC, Hebei University of Technology and Tianjin University of Technology further strengthened intersections with engineering and materials research. Overall, Tianjin’s collaboration model combined the advantages of local university clusters with cross-regional connectivity, balancing academic inquiry with industry demand in an application-oriented approach. Tianjin’s organizational network aligns with a policy-oriented RIS, in which government-supported universities and national research bodies coordinate innovation activities in strategically prioritized and application-driven domains.

### Author Co-Occurrence Analysis

**Fig 11:**
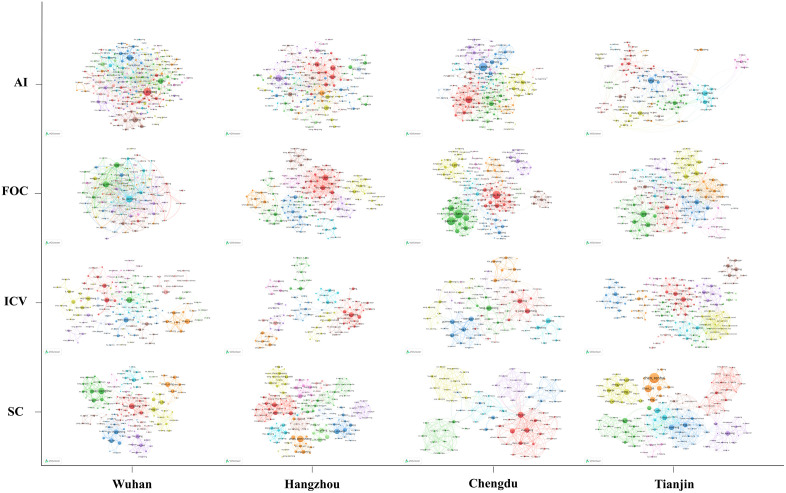
Co-occurrence networks of industrial collaboration authors across four cities and four technological domains.

By examining the co-occurrence networks of authors, we further identified clear inter-city differences in the micro-structure of regional innovation networks and gained deeper insight into each city’s research layout and comparative strengths across the four domains. Table 5 presents the top five authors ranked by document count, and Fig 11 illustrates the corresponding author co-occurrence networks across the four cities and four technological domains.

**Table 5:** Collaborating authors.

Address	industry	author	documents	citations	TLS
Wuhan	AI	zeng, zhigang	246	8916	634
		du, bo	163	5352	683
		gao, liang	146	5904	503
		ma, jiayi	139	15835	499
		wu, min	100	3784	400
	SC	miao, xiangshui	53	754	358
		huo, zongliang	44	383	316
		chen, rong	38	846	248
		wu, fei	35	346	198
		xie, changsheng	35	356	187
	ICV	cao, yue	20	183	97
		wan, shaohua	17	907	71
		chen, wei	16	130	74
		he, debiao	16	310	70
		xu, wei	15	454	78
	FOC	liu, deming	210	3324	1400
		tang, ming	175	2620	1146
		fu, songnian	145	2453	946
		zhang, xinliang	89	1982	501
		wang, jian	81	2110	386
Hangzhou	AI	wu, zheng-guang	99	5784	317
		li, xi	77	2138	373
		yu, jun	75	3033	331
		liu, yong	72	877	338
		yang, yi	71	2073	290
	SC	zhao, wen-sheng	17	185	83
		hu, yifeng	13	121	52
		song, zhitang	13	248	77
		liu, yan	11	67	105
		lai, tianshu	11	99	41
	ICV	fang, youtong	40	674	208
		liu, xing	39	637	203
		qiu, lin	38	636	199
		rodriguez, jose	25	468	128
		ma, jien	24	366	135
	FOC	dai, daoxin	109	3199	657
		shi, yaocheng	77	2629	463
		wang, d. n.	48	763	141
		liu, liu	48	936	347
		chen, daru	44	373	330
Chengdu	AI	xu, zeshui	362	11840	883
		li, tianrui	278	7291	1213
		yi, zhang	156	4725	596
		liao, huchang	133	6228	361
		shen, heng tao	126	8067	581
	SC	sun, bai	8	219	56
		jiang, dandan	6	63	32
		jin, lei	6	63	32
		zhao, yong	6	117	42
		huo, zongliang	6	63	32
	ICV	song, wensheng	21	607	80
		liu, xiaobo	20	511	69
		jiang, yangsheng	19	491	74
		yao, zhihong	19	487	72
		rodriguez, jose	18	442	97
	FOC	yan, lianshan	107	1522	658
		pan, wei	97	1457	616
		qiu, kun	94	1585	530
		luo, bin	60	949	392
		zou, xihua	60	870	397
Tianjin	AI	hu, qinghua	123	6311	492
		pang, yanwei	101	2916	397
		chen, shengyong	73	1134	340
		cheng, ming-ming	72	10026	333
		ji, zhong	70	1058	277
	SC	sun, jiaming	20	253	66
		yang, yang	19	303	68
		chen, renhai	17	218	74
		shao, zili	12	206	55
		wang, ru	10	42	62
	ICV	yang, hongjiu	19	179	64
		zuo, zhiqiang	15	329	60
		qiu, tie	13	855	57
		zhang, ting	13	317	59
		zhang, jie	11	234	50
	FOC	liu, tiegen	100	1788	660
		jiang, junfeng	64	1095	456
		wang, zhi	62	461	402
		zhang, hao	58	315	395
		liu, bo	53	305	363

In Wuhan, the AI and FOC networks appeared relatively dense and hierarchical, with a small number of highly central authors surrounded by many smaller contributors. In AI, Zeng, Zhigang ranked first with 246 papers and 8,916 citations, while Ma, Jiayi recorded 15,835 citations from 139 papers, suggesting high impact among leading scholars. In FOC, although the total output was much lower than in AI, the network remained closely connected, with Liu, Deming standing out at 210 publications. By contrast, ICV and SC showed a more multi-community structure, with stronger intra-group ties and weaker cross-group integration. Several authors in these two fields combined relatively modest output with comparatively high citation impact, suggesting the importance of original or application-oriented contributions.

A different pattern appeared in Hangzhou, where collaboration was less concentrated around a single core and more shaped by multiple medium-sized centers and bridging authors. In AI, Zheng, Guang occupied a central position with 99 papers and 5,784 citations, but no single-author dominance was evident. Authors such as Yang, Yi, Li, Xi, and Wu, Fei formed a relatively stable collaborative group, each with around 75 papers and more than 2,100 citations, indicating a comparatively balanced leadership structure. In FOC, Dai, Daoxin and Shi, Yaocheng anchored the main core with 109 and 77 papers, respectively, while cross-cluster ties linked materials, devices, and systems. The ICV and SC networks were also relatively weakly centralized, with multiple specialized communities and a visible degree of international collaboration. Overall, Hangzhou’s author collaboration structure was characterized by multi-core coordination and inter-cluster bridging.

In Chengdu, all four domains showed a multi-community and weak-centralization pattern, with several clusters of comparable size connected by a limited number of bridging authors. In AI, Xu, Zeshui stood out with 362 papers and 11,840 citations, the highest publication count among the four cities, while authors such as Shen, Heng Tao played linking roles across clusters. In FOC, a core–periphery belt emerged: Qiu, Kun sat at the center, connecting device-oriented teams (e.g., Yan, Lianshan; Pan, Wei) with application-focused micro-clusters through long ties. In ICV, the international scholar Rodriguez, Jose appeared again with differentiated collaboration paths: on the Chengdu side, the focus was on method-level innovation—leveraging FCS-MPC, RL, data-driven approaches, ESO, and event-triggered control to enhance robustness and performance—whereas on the Hangzhou side, emphasis lay on system-level deployment (e.g., VSG in islanded AC microgrids), using MPC and fuzzy-MPC to improve frequency and voltage dynamics and validate engineering feasibility through HIL. Both operated within an MPC framework yet complemented each other in control layer, technical levers, and validation settings. Notably, his Chengdu-side paper “Model-Predictive Control of Multilevel Inverters: Challenges, Recent Advances, and Trends” had 105 citations—substantially higher than the Hangzhou-side “Predictive Control of Voltage Source Inverter: An Online RL Solution” (39)—indicating deeper impact within Chengdu collaborations. In SC, multiple stable circles coalesced around Sun, Bai, with engineering-oriented teams such as Zhao, Yong forming tightly coupled, stable structures. Taken together, Chengdu’s author network can be described as a parallel multi-core structure supported by a small number of bridging nodes.

Tianjin showed a somewhat different profile. In AI, Cheng, Ming-Ming published 72 papers that received 10,026 citations, averaging about 140 citations per paper, the highest such figure among the four cities and fields, suggesting strong scholarly visibility despite a smaller output scale. By comparison, Chen, Shengyong published slightly more (73 papers) but with only 1,134 citations, further underscoring Cheng’s quality edge and the need for regional-level support. However, SC and ICV remained more limited in scale, with relatively balanced author distributions and multiple small communities rather than a dominant core. This produced a looser collaboration structure with weaker overall cohesion than in the other cities. Overall, Tianjin’s author collaboration pattern appeared more fragmented and weakly integrated, especially outside AI.

### Differentiated analysis and interpretation of regional industrial innovation pathways

Given the breadth and depth of the analysis generated by ChatGPT-DeepResearch, this section presents only the principal conclusions and core viewpoints in summary form, distilling the main findings on regional differentiation, innovation pathway characteristics, and policy-relevant implications; full details are provided in the Appendix. The complete LLM-based regional industrial and technological differentiation analysis can be found in S6 File. All supplementary information is supported by traceable references (peer-reviewed literature, industry reports, policy documents, or database records) and is documented in the Appendix with citation markers and retrieval protocols to ensure transparency and replicability. This summary distills only the most representative conclusions along the established evidence chain; each statement maps back to corresponding bibliometric and network-analytic evidence in the main text/Appendix, avoiding interpretations beyond the data’s scope. Readers may use the Appendix source list and citation markers to locate specific city–industry evidence items (keywords, representative studies, and network-structure features) for verification.

Before turning to the city-level analysis, we briefly outline the differentiated regional innovation pathways observed across the four cities. Wuhan is characterized by a research- and state-coordinated system; Hangzhou by a platform- and firm-led, market-oriented system; Chengdu by a science-based, policy-supported system; and Tianjin by a transitional, application-oriented system.

#### Wuhan.

(1) Differentiated Analysis

Wuhan’s regional innovation pathway is best understood as a research- and state-coordinated system that is increasingly oriented toward bridging “lab-to-market” gaps, rather than a fully market-driven entrepreneurial ecosystem. The city’s dominant innovation actors remain top universities and major state-backed industrial platforms, which give Wuhan a strong knowledge-production base and the capacity to mobilize resources at scale. This structure favors mission-oriented advances and large, strategically significant projects, but it also creates a recurring system tension: commercialization and firm formation tend to lag behind scientific capability. The result is a pathway characterized by high research intensity, strong public coordination, and selective industrial anchoring, with innovation outcomes concentrated where long-term investment and infrastructure matter most.

Across AI, FOC, ICV, and SC, Wuhan’s technological evolution shows path dependence reinforced by institutional thickness: long-standing strengths in engineering-oriented research and industrial organization make the city effective at building and sustaining “hard-tech” trajectories. At the same time, the system often relies on large incumbents or external application partners to translate capability into large-scale deployment, which can dilute local entrepreneurial dynamism even when deployment appears impressive. Structurally, Wuhan is less platform- and venture-driven than Hangzhou and Chengdu, and differs from Tianjin by having a clearer knowledge-production core and stronger endogenous specialization potential.

In comparative terms, Wuhan’s pathway is best described as “anchored specialization with emerging convergence”: it consolidates globally competitive deep-tech pillars and increasingly seeks new growth through cross-domain integration, but it must strengthen intermediary mechanisms that convert research excellence into a broader base of local firms and innovation diffusion. This is why the city’s evolution is both robust and constrained: powerful in generating frontier capability, yet still working to institutionalize the connective tissue that makes innovation self-reinforcing at the ecosystem level.

(2) Policy Recommendations
  - **Leverage Optics–Semiconductor Synergies to Build a Photonics Innovation Hub**

This policy responds to Wuhan’s core opportunity revealed in the Differentiated Analysis: Wuhan’s pathway is anchored in deep, engineering-oriented capability, but its next-stage differentiation depends on creating new growth through structured convergence, not just parallel excellence. The issue is a coordination and connectivity gap: optics/photonics and semiconductors co-exist yet do not fully interact as an integrated innovation trajectory, limiting Wuhan’s ability to create a distinctive frontier niche that compounds its existing strengths.

The innovation-system mechanism is “related variety” and knowledge-bridge formation: by intentionally coupling actor networks that historically evolved separately, Wuhan can strengthen spillovers, raise absorptive capacity on both sides, and reduce fragmentation that keeps promising ideas from finding local industrial uptake. This is especially aligned with Wuhan’s research-and-state-coordinated structure, which is well-suited to building shared infrastructure and long-gestation deep-tech programs.

In practice, the policy operates by institutionalizing joint platforms (consortia, shared labs, joint R&D programs) that make cross-domain collaboration the default rather than an exception. Government can steer resources toward cross-partner projects, align incentives for universities and major enterprises to co-develop prototypes and standards, and use shared facilities to create daily interaction among engineers and researchers. The feasibility advantage is that Wuhan already has the concentrated institutional base needed to run a durable hub model; the key is designing mechanisms that make cross-sector collaboration routine, with clear IP rules and staged milestones so the system produces cumulative outcomes rather than one-off projects.

- **Expand University–Industry Translational Platforms to Commercialize AI and ICV Technologies**

This policy directly addresses the main weakness in Wuhan’s Differentiated Analysis: Wuhan’s pathway is strong in knowledge production and coordinated capability-building, yet it remains constrained by an insufficient “middle layer” that converts research into scalable firms, products, and local market leadership—particularly in AI and ICV where visible deployment can still rely heavily on non-local application actors.

The innovation-system logic is to bridge a value-chain discontinuity by building translational institutions that connect knowledge producers with users and entrepreneurs. Dedicated platforms increase network density across universities, firms, and end-users, reducing fragmentation and enabling interactive learning under real deployment constraints. This strengthens Wuhan’s ecosystem self-reinforcement: instead of research outputs staying isolated, they flow into prototypes, pilots, spin-offs, and procurement pathways that generate local growth loops.

Operationally, the policy works through sector-specific incubators and living labs, joint R&D centers with commercialization mandates, and professionalized, industry-embedded tech-transfer structures. The city can pair these platforms with targeted financing, evaluation criteria that reward commercialization outcomes, and procurement mechanisms that bring local solutions into real-world systems earlier. In Wuhan’s context, this is feasible because strong research cores and substantial industrial/public-sector demand already exist; the missing step is institutionalizing intermediaries that repeatedly convert demand and research into local firms and scalable AI/ICV products, making commercialization a system feature rather than a case-by-case outcome.

#### Hangzhou.

(1) Differentiated Analysis

Hangzhou’s regional innovation pathway is a hybrid, market-deepened system where large private firms and platform organizations play the central coordinating role, and government acts in an entrepreneurial, enabling mode rather than as the primary director of innovation. The dominant innovation actors—digital giants, systems integrators, and well-connected universities and labs—create a structure that excels at rapid translation of frontier knowledge into scalable systems, particularly where software, data, and deployment environments matter. This produces a pathway that is less about single-technology breakthroughs in isolation and more about system-level integration, where products and industrial solutions pull R&D forward through iterative deployment cycles.

Across AI, FOC, ICV, and SC, Hangzhou’s technological evolution is characterized by application-led upgrading and platform-mediated coordination. Innovation is frequently organized around ecosystem needs (e.g., digital infrastructure, intelligent systems, real-world trials), so hardware-facing domains tend to develop as enabling layers within broader solution stacks rather than as stand-alone manufacturing-centered trajectories. This is the core structural difference from Wuhan: Hangzhou’s innovation strength lies not primarily in state-led deep-tech concentration, but in tight feedback loops between market demand, platform capabilities, and research, reinforced by abundant finance and high talent attraction.

Comparatively, Hangzhou also differs from Chengdu by having a more mature private-sector leadership structure and stronger endogenous commercialization channels, and from Tianjin by possessing a more balanced and self-reinforcing actor configuration rather than a transitional, capacity-heavy system. The city’s pathway can be summarized as “platform-driven systemic innovation”: it combines strong firms, responsive governance, and capable research institutions to move quickly from knowledge to use, while its key structural risk is ensuring sufficient depth in foundational hardware domains to sustain autonomy and resilience as competition shifts toward integrated artificial Intelligence of Things (AIoT) and advanced components.

(2) Policy Recommendations
  - **Deepen Integration of Digital Giants with Local Manufacturing for AIoT Leadership**

This policy targets the key system opportunity identified in Hangzhou’s Differentiated Analysis: Hangzhou excels at platform-driven, system-level innovation, but it must ensure that its digital strengths translate into broad industrial upgrading and hardware resilience, not only into software-centric growth. The issue is a structural imbalance: digital giants create powerful feedback loops, yet parts of the manufacturing base and foundational hardware capabilities can remain less deeply integrated into the city’s innovation engine, risking vulnerability as competition shifts toward AIoT and cyber-physical systems.

The underlying innovation-system mechanism is to expand “related variety” by tightening cross-sector networks between platform firms and manufacturers. In Hangzhou’s pathway, large firms act as orchestrators: if they become systematic catalysts for manufacturing upgrading, the region strengthens knowledge spillovers, accelerates diffusion, and forms new hybrid trajectories where data, AI models, devices, and production systems co-evolve. This reinforces Hangzhou’s distinctive advantage—rapid translation from frontier knowledge to real-world systems—while distributing benefits across a wider industrial base.

In practice, the policy operates through formal partnership structures (alliances, co-investment, shared platforms) that align incentives for digital firms to provide AIoT solutions and for manufacturers to adopt and co-develop them. Government’s role is to coordinate, reduce transaction costs (standards, data-sharing governance, IP rules), and de-risk adoption via pilots that scale. Because Hangzhou already has strong firms, research institutions, and supportive governance capacity, feasibility hinges on making integration systemic: turning isolated pilots into repeatable pathways where manufacturing demand shapes platform innovation, and platform capabilities upgrade manufacturing productivity and product complexity, thereby anchoring Hangzhou’s AIoT leadership in an ecosystem that is both scalable and resilient.

- **Foster Frontier Research and Startup Ecosystems in Semiconductors and Photonics**

This policy responds to a vulnerability implicit in Hangzhou’s Differentiated Analysis: Hangzhou’s system excels at application-led integration, but sustained leadership in AIoT increasingly requires deeper capability in foundational hardware domains and a thicker frontier research/startup layer that can supply critical components and next-generation technologies. The issue is not simply “insufficient output”, but a pathway-level risk: if core hardware innovation remains thin or externally dependent, Hangzhou’s platform-driven ecosystem may face constraints in autonomy, resilience, and long-term competitiveness.

The innovation-system mechanism is to strengthen the knowledge-production frontier while building commercialization channels appropriate to hardware: frontier labs, shared infrastructure, and specialized startup support reduce entry barriers and increase the probability that research translates into locally embedded firms. This complements Hangzhou’s existing strengths—capital availability, talent attraction, and strong system integrators—by adding a missing layer of specialized producers and startups that can plug into platform ecosystems.

Operationally, the policy works by concentrating resources into frontier research platforms and specialized incubators, using targeted funding and infrastructure to build critical mass in semiconductors and photonics, and creating interfaces where systems firms can act as early adopters and demand anchors for local hardware startups. Government can coordinate long-horizon investment and reduce coordination failures, while firms provide application pull and market access. In Hangzhou’s context, feasibility depends on aligning this effort with its pathway logic: making frontier hardware ecosystems not a parallel ambition, but a reinforcing layer that strengthens the city’s integrated AIoT trajectory by ensuring that enabling components and frontier knowledge increasingly originate—and scale—within the region.

#### Chengdu.

(1) Differentiated Analysis

Chengdu’s regional innovation pathway is a broad-based, policy-supported system with strong science and engineering capability but a still-maturing structure for endogenous enterprise formation and outward commercialization. The dominant innovation actors are multi-polar academic institutions and large strategic organizations, increasingly complemented by branches and projects of national tech firms. This configuration creates a pathway where knowledge generation is comparatively strong and diversified, while the conversion of that knowledge into locally rooted high-tech firms and scalable industrial leadership remains a central system bottleneck.

Across AI, FOC, ICV, and SC, Chengdu’s evolution shows a pattern of capability accumulation followed by selective acceleration through strategic partnerships and policy focus. The system is oriented toward building regional strength and upgrading western China’s technological profile through coordinated initiatives, but it also reflects a structural dependence on external anchors for certain high-visibility advances. Unlike Hangzhou’s platform-centric market pull, Chengdu’s pathway is more often institutionally and strategically guided, and unlike Wuhan’s concentrated deep-tech anchoring, Chengdu’s innovation system is more distributed and collaborative, with a notable emphasis on regional integration and cross-boundary learning.

The city’s distinguishing pathway characteristic is “networked capability-building”: it develops a wide innovation base, leverages defense-civil and strategic-policy channels to extend into new applications, and increasingly positions itself as a western hub that connects local strengths to national and global flows. Its comparative challenge is that a system can be rich in research and projects yet still lack the density of local firms that makes innovation self-reinforcing. Hence, Chengdu’s trajectory is differentiated by the need to thicken the entrepreneurial and commercialization layer while simultaneously deepening inter-regional linkages that prevent lock-in and expand market reach.

(2) Policy Recommendations
  - **Cultivate a Homegrown High-Tech Enterprise Ecosystem to Complement Research Strengths**

This policy directly targets the central bottleneck in Chengdu’s Differentiated Analysis: Chengdu has broad science and engineering capability and a diversified institutional base, yet it lacks a proportionate density of locally rooted high-tech enterprises that convert knowledge into sustained industrial leadership. The issue is pathway-level: if innovation remains concentrated in academia or imported through external firm branches, the system’s growth loops remain weaker, and the region becomes more dependent on shifting external strategies.

The innovation-system mechanism is ecosystem thickening through new firm formation: a stronger entrepreneurial layer fills structural holes between research and markets by adding intermediaries (incubation, mentorship, finance, regulatory sandboxes) and by normalizing mobility from labs into ventures. This builds absorptive capacity in a specifically entrepreneurial sense—turning Chengdu’s talent base into local experimentation, specialization, and cluster deepening—so innovation becomes self-reinforcing rather than episodic.

In practice, the policy operates by creating domain-focused incubators/accelerators and structured partnerships that turn large organizations and industrial actors into testbeds and early customers for local startups, while government reduces friction through streamlined procedures and targeted procurement channels. The key feasibility lever in Chengdu is cultural-institutional alignment: because the system has historically been academia- and SOE-influenced, the policy must create repeated, credible incentives for entrepreneurship, not just one-time funding. If executed as a sustained ecosystem program, the city’s innovation pathway shifts from “capability-rich, enterprise-thin” to “capability-plus-firm-density”, enabling Chengdu to retain talent, embed IP locally, and develop indigenous champions that complement (rather than depend on) external anchors.

- **Strengthen Inter-Regional Innovation Linkages to Leverage National and Global Knowledge Flows**

This policy addresses a second pathway requirement in Chengdu’s Differentiated Analysis: Chengdu’s strength lies in networked capability-building, and its continued acceleration depends on institutionalizing openness to systematically absorb external knowledge and scale local innovations beyond the local market. The issue is that an internally strong system can still face limits in frontier breadth, commercialization reach, and capital access if linkages remain ad hoc rather than structured.

The innovation-system logic is to expand the effective boundary of the regional innovation system by increasing cross-regional connectivity and preventing lock-in. Mechanistically, formal linkages improve interactive learning, diversify inputs, and raise resilience by connecting Chengdu’s actors to complementary capabilities elsewhere (advanced markets, specialized suppliers, global standards arenas). This is especially compatible with Chengdu’s distributed actor configuration: stronger linkages amplify its role as a western hub and reduce dependence on any single internal trajectory.

Operationally, the policy works through structured joint programs, talent mobility channels, and cross-regional platforms that lower transaction costs for collaboration—plus mechanisms that connect firms to broader financing and market networks. In Chengdu’s context, feasibility is strengthened by existing national strategies encouraging west–east cooperation and by Chengdu’s emerging global ties; the implementation challenge is to move from symbolic collaboration to durable institutional arrangements that repeatedly generate joint R&D, commercialization partnerships, and scaling pathways. Executed well, this policy makes Chengdu’s innovation pathway more outward-facing and scalable, turning network openness into a stable source of comparative advantage rather than a temporary boost.

#### Tianjin.

(1) Differentiated Analysis

Tianjin’s regional innovation pathway is best characterized as transitional and supportive, anchored in substantial engineering and industrial capacity but still working to convert that capacity into sustained, distinctive frontier innovation output. Its dominant innovation actors include strong universities and numerous state-oriented industrial and research organizations, with a comparatively weaker presence of dynamic local private-sector champions. This yields an innovation system that is capacity-rich but output-constrained, where top-down projects and institutional routines can sustain large-scale activity, yet entrepreneurial variety and market-facing experimentation remain thinner than in the other cities.

Across AI, FOC, ICV, and SC, Tianjin’s evolution reflects a pattern of applied, manufacturing-adjacent innovation rather than ecosystem-led frontier scaling. The city’s core advantage is that it can operationalize and absorb advanced technologies into industrial environments, but its structural challenge is differentiating beyond a role as a “production and application base.” What makes Tianjin distinct in this four-city comparison is its deep embedding in the Beijing–Tianjin–Hebei mega-region, which creates a unique combination of opportunity and constraint: proximity enables spillovers of talent, capital, and high-level research influence, while also intensifying the risk of being overshadowed and positioned as secondary.

Compared with Wuhan’s deep-tech anchoring, Hangzhou’s platform-driven feedback loops, and Chengdu’s networked capability-building, Tianjin’s differentiator is “borrowed strength with industrial grounding”: it can leverage regional integration to offset gaps in frontier origination, while using its manufacturing and engineering base to build applied innovation leadership—if institutional and actor-mix constraints are actively addressed.

(2) Policy Recommendations
  - **Accelerate Industrial Modernization through AI and Smart Technologies in Tianjin’s Manufacturing Heartland**

This policy responds to the core issue identified in Tianjin’s Differentiated Analysis: Tianjin’s innovation system is capacity-rich and industrially grounded, yet it risks remaining supportive and transitional if advanced technologies do not systematically penetrate its manufacturing and SOE-heavy base. The opportunity is to turn Tianjin’s greatest structural asset—its industrial infrastructure and engineering depth—into an innovation flywheel by making firms active users and co-developers of AI and smart technologies rather than passive adopters.

The innovation-system mechanism is user–producer interaction: when industrial firms become sophisticated users, they generate demand signals, test environments, and iterative learning loops that accelerate local innovation and diffusion. In Tianjin’s context, this matters because the private entrepreneurial layer is relatively thin; upgrading incumbent industrial actors is the most realistic near-term route to ecosystem dynamism. It also shifts the system from top-down, project-only outputs toward repeatable productivity and innovation gains embedded in firms.

In practice, the policy operates through targeted Industry 4.0 transformation programs: factory-level AI deployment, industrial data infrastructure, automation upgrades, and joint applied R&D between universities and manufacturing enterprises, with government acting as coordinator and de-risking investor. The feasibility advantage is that Tianjin can implement at scale given its concentration of manufacturing sites; the key is governance design that rewards adoption and co-innovation inside firms (including SOEs), so modernization produces cumulative capability rather than isolated pilots. If successful, Tianjin’s pathway differentiates through “applied innovation leadership”: it becomes a place where advanced AI and smart systems are reliably engineered into production, raising local innovation output while strengthening its role in national high-tech supply chains.

- **Foster a Collaborative Beijing-Tianjin-Hebei Innovation Cluster to Leverage Complementary Strengths**

This policy targets Tianjin’s most distinctive structural condition highlighted in the Differentiated Analysis: Tianjin’s deep embedding in the Jing-Jin-Ji region creates both the risk of being overshadowed and the opportunity to achieve “borrowed strength” by integrating into a mega-regional innovation system. The issue is not simply cooperation in principle, but the lack of sufficiently institutionalized arrangements that let Tianjin access Beijing’s frontier research, talent, and capital while anchoring activities that build Tianjin’s own specialization and output.

The innovation-system mechanism is mega-regional alignment: by harmonizing institutions, enabling mobility of people/capital, and co-investing in shared platforms and testbeds, the cluster increases network density and scale effects, reducing fragmentation across administrative boundaries. For Tianjin, the key mechanism is role specialization with integration—positioning Tianjin as the applied R&D, prototyping, and pilot-production node that complements Beijing’s research/design strengths, rather than duplicating them.

Operationally, the policy works through joint alliances, flagship cross-city labs/projects, unified talent and capital policies, cross-regional incubation, and harmonized regulatory sandboxes that let innovations scale along the Beijing–Tianjin corridor. Feasibility is strong because the region is already a national strategic initiative; the practical challenge is governance: collaboration must be designed so Tianjin gains durable capability and identity (anchored projects, local firm growth, visible specialization), not only spillover work. If executed with clear division of labor and shared incentives, Tianjin’s pathway can shift from “supportive adjacency” to “integrated specialization”, raising both output and differentiation within China’s innovation landscape.

## Summary and discussion

### Main contributions

Grounded in the RIS perspective, this study contributes to the literature by advancing a unified, data-driven framework for examining inter-city heterogeneity in high-tech development. The framework operationalizes key RIS components—dominant R&D actors, knowledge production and transformation mechanisms, and institutional collaboration structures—through the integrated use of bibliometric statistics, co-word analysis, collaboration network modeling, and LLM-assisted semantic interpretation. By embedding these elements within a single analytical workflow, the study provides a reproducible approach for empirically investigating how different urban innovation systems organize scientific research, technological application, and cross-actor coordination across industries, thereby moving beyond purely descriptive comparisons.

Methodologically, the study introduces a scalable pipeline for processing large-scale textual innovation data in RIS-oriented research. Rule-based procedures are combined with the capabilities of large language models to construct a standardized and noise-reduced keyword system, improving the reliability of hotspot identification, temporal trend analysis, and cross-regional comparison. The incorporation of a three-tier technical taxonomy—foundational, core, and frontier technologies—enables the differentiation of technological roles within innovation systems, allowing structural change and frontier orientation to be quantitatively assessed rather than inferred qualitatively.

Empirically, the study provides comparative evidence on how distinct RIS configurations shape differentiated development trajectories across Wuhan, Hangzhou, Chengdu, and Tianjin in AI, FOC, ICV, and SC. Joint analysis of publications, patents, and organizational collaboration networks reveals how cities rely on different combinations of universities, research institutes, firms, and policy instruments to drive innovation. The LLM-assisted interpretive layer situates these quantitative patterns within regional industrial and policy contexts, facilitating the translation of empirical findings into policy-relevant insights while preserving the integrity of statistical inference.

Overall, these contributions strengthen the link between RIS theory and empirical measurement, enhance the interpretive depth of regional innovation analysis, and offer a transferable framework for studying urban technological differentiation across regions and sectors.

### Scholarly and Practical Significance

This study yields both theoretical and practical significance for research on regional industrial and technological development. From a scholarly perspective, the integrated framework—combining bibliometric statistics, co-word analysis, collaboration network modeling, and LLM-assisted interpretation—extends existing approaches in scientometrics and knowledge mapping by enabling a more structured and interpretable analysis of regional innovation systems. The introduction of LLMs into data preprocessing improves the reliability of keyword-based analysis through systematic removal of non-informative terms, abbreviation recognition, and synonym normalization, addressing long-standing challenges in bibliometric studies related to semantic fragmentation and interpretive ambiguity. Rather than replacing conventional quantitative methods, this human–AI collaborative workflow enhances analytical consistency and semantic coherence, illustrating a feasible pathway for integrating LLMs into empirical innovation research.

At the same time, the three-tier technical classification of foundational, core, and frontier technologies provides a replicable semantic instrument for examining technological evolution within and across regions. By distinguishing different functional roles of technologies, this classification enables a more precise depiction of hierarchical structures and dynamic transitions in innovation systems, thereby strengthening the analytical linkage between technological change and RIS theory.

From a practical standpoint, the cross-regional and cross-industry analyses reveal differentiated configurations and evolutionary trajectories of high-tech development, offering evidence-based insights for regional policy design. By identifying frontier technologies and tracking their temporal dynamics, the framework supports forward-looking industrial planning and helps policymakers mitigate homogeneous competition and inefficient resource allocation. Moreover, the LLM-assisted interpretive layer translates complex patterns embedded in research hotspots and collaboration networks into policy-relevant insights, enhancing the accessibility and applicability of empirical findings for science-and-technology governance and strategic decision-making.

### Limitations

Despite the systematic exploration undertaken in this study, several limitations should be acknowledged. First, the data sources are primarily drawn from the Web of Science and the national patent information platform. While these datasets provide authoritative and internationally comparable coverage of scholarly publications and formal technological outputs, they do not fully capture gray literature, conference proceedings, firm-level R&D reports, or unpublished patents. As a result, certain innovation activities—particularly those embedded in enterprises or informal knowledge channels—may be underrepresented, potentially introducing partial coverage bias.

Second, the construction of retrieval strategies inevitably influences the scope of the dataset. In domains where fine-grained and universally accepted classification schemes are lacking, keyword-based queries may either omit relevant records or introduce cross-domain noise. Although expert taxonomies and iterative refinement were applied to mitigate this issue, some degree of incompleteness or contamination in the retrieved corpus cannot be entirely ruled out.

Third, bibliometric and network-based approaches primarily capture output volume, co-occurrence patterns, and structural relationships. While these methods are effective for identifying technological distributions, collaboration structures, and evolutionary trends, they are less capable of directly assessing research quality, technological novelty, or real-world economic impact. Consequently, the indicators used in this study should be interpreted as proxies for innovation activity rather than comprehensive measures of technological performance.

Fourth, although large language models were introduced to support keyword standardization and semantic interpretation, their outputs may still be influenced by training data, prompt design, and inherent semantic ambiguity. LLM-assisted processing may therefore introduce residual noise or bias, underscoring the importance of treating such tools as complementary aids rather than autonomous analytical agents.

Finally, the proposed framework places greater emphasis on structural patterns and temporal trends than on explicitly modeling dynamic interactions among policy environments, industrial value chains, and market demand. This limits the framework’s capacity to capture short-term shocks or feedback mechanisms, and future research could benefit from integrating policy dynamics, firm-level data, and market indicators to enhance explanatory power and predictive relevance.

## Supporting information

S1 File Expert-defined technical taxonomy. This file covers nearly all technology-related industries. It provides a comprehensive classification framework that spans traditional sectors as well as emerging domains.
(ZIP)

S2 File Identify abbreviations prompt. This file contains the prompt used to accurately determine whether an input term is an abbreviation or acronym. It guides the model to identify shortened forms of words or phrases and distinguish them from general terms.
(TXT)

S3 File Generate equivalence of taxonomy. The prompt instructs LLM pre-generate equivalence sets for taxonomy entries.
(TXT)

S4 File Equivalence mapping of semantically identical keywords. This file identifies and normalizes semantically identical keywords that appear in different written forms, generating an equivalence mapping table. Normalization is applied only when the same concept occurs in at least two distinct expressions within the file.
(TXT)

S5 File LLM analysis of regional technological differentiation. This file contains the prompt designed for ChatGPT-DeepResearch feature.
(TXT)

S6 File Differentiated Analysis and Interpretation of Regional Industrial Technologies (LLM). This file provides the full LLM-generated Section 4.5, which includes an analytical summary of regional technological differentiation and corresponding policy recommendations.
(DOCX)
