# Supplementary material for: Data-driven differentiation analysis of urban high-tech industries: Research on bibliometrics and large language models
Source: PLoS One. 2026 May 14;21(5):e0348590. doi: 10.1371/journal.pone.0348590 (PMC13175361; doi:10.1371/journal.pone.0348590)
Supplement: S6 File — This file provides the full LLM-generated Section 4.5, which includes an analytical summary of regional technological differentiation and corresponding policy recommendations. (DOCX) [file pone.0348590.s006.docx]

# Regional Innovation Pathways in Wuhan, Hangzhou, Chengdu, and Tianjin (AI, FOC, ICV, SC)

## Wuhan

### Regional Differentiation Summary

Wuhan’s innovation pathway is anchored by a robust **university-driven research base** and long-standing industrial clusters, giving the city distinct strengths in **fiber-optic communications (FOC)** and emerging **semiconductor (storage chip) manufacturing**, alongside a rapidly growing **AI sector**. The city’s top contributors to knowledge outputs are its major universities—Huazhong University of Science & Technology (HUST) and Wuhan University—whose prolific publication records in **AI** and related fields far outstrip any local corporate R&D output. This academic dominance is evident in Wuhan’s AI domain: HUST alone produced over 2,500 publications (2016–2025) with ~79,000 citations, forming the core of an extensive local co-author network. **Collaboration patterns** in Wuhan thus center on inter-university linkages and partnerships with national research institutes (e.g. Chinese Academy of Sciences’ local branches) rather than on home-grown tech giants. A comparative absence of large indigenous tech firms in AI (versus, say, Hangzhou’s Alibaba) hints at a **structural weakness**: the translation of academic research into industry has been slower, potentially limiting Wuhan’s agility in fast-moving AI frontiers despite its high publication volume. Nevertheless, Wuhan’s universities have carved out niche specialties—**remote sensing AI** and **pattern recognition** at Wuhan University, and **neural networks and optimization** at HUST—yielding influential research (e.g. widely cited deep learning methods for hyperspectral image analysis and feature matching). Such outputs underscore Wuhan’s capacity in *foundational and core AI research*, even as the city now pivots to increase its share of **frontier AI topics** like large language models (LLMs) and multimodal AI. Notably, Wuhan’s AI publications saw frontier-technology keywords (e.g. “attention mechanism”, “LLMs”) rise to ~19% of total tech keywords by 2025, catching up with Hangzhou and Chengdu. This indicates a late but accelerating shift toward **future-oriented AI research**, likely spurred by national initiatives in recent years.

In **fiber-optic communications (FOC)**, Wuhan enjoys a historical **first-mover advantage** rooted in its “Optics Valley of China” industrial zone. The city hosts leading FOC enterprises such as *Yangtze Optical Fibre and Cable Co.* (YOFC) and *FiberHome Technologies*, alongside specialized labs (e.g. the Optics Valley Laboratory). These actors, together with HUST’s renowned optics school, form a tightly knit innovation system that blends academic R&D with manufacturing. Empirically, Wuhan’s FOC output from 2016–2025 is both high-volume and high-impact: HUST contributed over 1,000 FOC-related publications (with 16,500+ citations), often co-authored with industry researchers (YOFC appears among top contributors). The **technology layout** in Wuhan-FOC shows an emphasis on cutting-edge photonics devices—“photodetector” is the top frontier keyword—indicating strong activity in advanced optical components. Indeed, Wuhan’s FOC trajectory evolved from basic fiber manufacturing in earlier years to **frontier photonic integration** more recently, as reflected by the growing occurrence of frontier terms like integrated sensing and underwater laser communication in local research. This progression suggests a **path-dependent evolution**: Wuhan leveraged its legacy in optical fiber production to move upstream into photonic chips and next-gen optical networks. By comparison, other cities lack Wuhan’s deep optical supply chain; for instance, Hangzhou’s FOC research is mostly academic (Zhejiang University) with fewer industrial players, and Tianjin’s output is a fraction of Wuhan’s. Underlying Wuhan’s differentiation is a **strong institutional support system**: the East Lake High-Tech Zone (Optics Valley) not only houses companies and national key labs but also offers aggressive incentives to attract global fiber and semiconductor experts[[1]](https://www.yicaiglobal.com/news/wuhans-high-tech-industry-hub-offers-incentives-of-up-to-usd14-million-per-project-to-lure-global-talent#:~:text=The%20fund%20will%20focus%20on,tech%20industry%20hub)[[2]](https://www.yicaiglobal.com/news/wuhans-high-tech-industry-hub-offers-incentives-of-up-to-usd14-million-per-project-to-lure-global-talent#:~:text=The%20EDZ%20is%20a%20high,percent%20from%20a%20year%20earlier). As a result, Wuhan accounts for roughly 10% of the **global fiber-optic cable market** through YOFC[[3]](https://thenetworkinstallers.com/blog/best-fiber-optic-manufacturers/#:~:text=Prysmian%20Group%20%289,of%20the%20world%E2%80%99s%20largest%20fiber), a remarkable statistic illustrating how *regional specialization* in FOC translated into worldwide industrial leadership.

Wuhan’s nascent strength in **storage chips (SC)** further exemplifies its pattern of linking research with industrial policy. Traditionally not a semiconductor hub, Wuhan surged ahead around 2016 by establishing *Yangtze Memory Technologies Co.* (YMTC) – a national flagship project in 3D NAND flash memory – in the Optics Valley zone. The impact has been twofold: First, local research and patenting in SC climbed significantly (Wuhan’s SC-related publications grew to 124 in 2025 from virtually negligible a decade prior), and second, the nature of Wuhan’s SC innovation is distinctly **frontier-oriented**. Almost **100% of Wuhan’s SC technology keywords** since 2018 are classified as *“Frontier”*, with “non-volatile memory” dominating the discourse. This indicates that Wuhan skipped incremental steps and jumped directly into **cutting-edge memory chip R&D**, aligning with YMTC’s mission to compete with global leaders. Indeed, by 2023 YMTC managed a “surprise technology leap” by producing one of the world’s most advanced 3D NAND chips despite U.S. sanctions[[4]](https://www.scmp.com/business/article/3239310/chinas-ymtc-makes-worlds-most-advanced-memory-chip-surprise-technology-leap-techinsights-report#:~:text=Yangtze%20Memory%20Technologies%20Co%20,to%20a%20report%20by%20TechInights)[[5]](https://www.scmp.com/business/article/3239310/chinas-ymtc-makes-worlds-most-advanced-memory-chip-surprise-technology-leap-techinsights-report#:~:text=3D%20NAND%20memory%20is%20at,artificial%20intelligence%20and%20machine%20learning). Such achievements underscore Wuhan’s capability to integrate large-scale industrial investment with high-end innovation. The presence of YMTC also diversifies Wuhan’s **innovation actor mix**: unlike AI and FOC, where academia prevails, in SC a corporate actor (albeit state-backed) is at the forefront, driving application-oriented R&D (e.g. Xtacking® chip architecture). However, a **latent challenge** is the heavy reliance on a single corporate anchor; Wuhan’s SC ecosystem is not as broad-based (in terms of number of firms or startups) as, say, Shanghai’s. The city’s pathway here is an example of **institutionally steered path creation** – central and local governments deliberately seeded a new sector, which now needs nurturing of supportive SMEs, supplier networks, and talent pools beyond the flagship firm.

In the **intelligent and connected vehicles (ICV)** arena, Wuhan’s trajectory again reflects **government-enabled experimentation** and partnerships rather than organic private-sector leadership. Wuhan historically is an automotive manufacturing base (headquarters of state-owned Dongfeng Motor Corporation), but until recently it lagged coastal cities in automotive R&D. The past five years have seen Wuhan rapidly embrace autonomous driving trials and V2X infrastructure, making it a national testbed. By 2024, Wuhan had approved a fleet of nearly 500 *robotaxis* operating on 35% of the city’s roads – an unprecedented scale in China, outpacing even Beijing and Shenzhen in deployment aggressiveness[[6]](https://www.scmp.com/tech/tech-trends/article/3271143/wuhan-driverless-taxis-offer-peek-future-intracity-transport-china#:~:text=While%20cities%20such%20as%20Beijing,a%20pioneer%20of%20the%20technology). This bold approach, supported by city regulators, attracted **technology giants** like Baidu (which operates Apollo Go robotaxis in Wuhan) and mobilized local manufacturers like Dongfeng to trial self-driving shuttles[[7]](https://www.scmp.com/tech/tech-trends/article/3271143/wuhan-driverless-taxis-offer-peek-future-intracity-transport-china#:~:text=Operating%20under%20the%20brand%20name,and%20shuttles%20in%20the%20city). The co-occurrence data confirms Wuhan’s ICV focus on frontier technologies such as **LiDAR** and advanced driver-assistance (ADAS): “laser radar” and “ADAS” rank among top frontier keywords in Wuhan’s ICV publications. These emphases suggest Wuhan’s researchers and engineers are working on sensor suites and autonomous driving algorithms – knowledge areas likely bolstered by collaboration between universities (for AI algorithms) and firms like Dongfeng (for vehicular engineering). The ICV development in Wuhan has been undergirded by local policy initiatives like the Sino-French Wuhan Ecological Demonstration City, which in 2021 hosted China’s first dedicated autonomous vehicle test zone[[8]](https://english.wuhan.gov.cn/H_1/NWP/202003/t20200316_954175.shtml#:~:text=China%27s%20first%20test%20zone%20for,Demonstration%20City%20in%20Wuhan%2C). Such initiatives indicate a **supportive governance environment** that reduces regulatory barriers for innovation. Wuhan’s differentiation, compared to peer cities, lies in leveraging its strong **public-sector orchestration** to integrate new technology (AI) into a traditional industry (automotive). While Hangzhou and others also pursue ICV, Wuhan’s large-scale pilot deployment and involvement of multiple actor types (municipal authorities, SOEs, and tech firms) highlight a **systemic approach**: the city effectively functions as a living lab for ICV, which accelerates learning and public acceptance.

Overall, Wuhan’s regional innovation pathway is characterized by **high-impact academic research cores, state-facilitated industrial upgrading, and an increasing orientation toward frontier technologies** across all four domains. The interplay of actor configurations and technological change in Wuhan can be summarized as follows: **strong research universities and national labs provide foundational knowledge**, which, when coupled with **strategic government backing and the presence of key state-owned enterprises**, has enabled Wuhan to pioneer new technological directions (FOC in earlier decades, now AI and chips) within a relatively short time. This has given rise to a *hybrid innovation system* – one that excels in scientific output and is beginning to translate that into industrial leadership in select areas. The **latent mechanisms** behind Wuhan’s differentiation include path dependence from its optics legacy (leading to related diversification in photonics and semiconductors), and a **co-evolution of institutions and technology** – for example, Wuhan’s selection as the testbed for China’s “embodied AI” AGI initiative. In fact, in 2023 the Chinese Academy of Sciences’ Institute of Automation, Huawei, and a Peking University-led consortium launched an ambitious *AGI pilot project in Wuhan* to embed AI in real-life environments[[9]](https://cset.georgetown.edu/publication/wuhans-ai-development/#:~:text=approaches%20to%20AGI%20that%20involve,surroundings%2C%20learning%20as%20it%20proceeds)[[10]](https://cset.georgetown.edu/publication/wuhans-ai-development/#:~:text=The%20test%20bed%20for%20this,all%20aspects%20of%20daily%20life). This project, involving a “social simulator” spread across Wuhan’s industries and public services, exemplifies how Wuhan’s **policy environment proactively shapes innovation** by inviting cutting-edge experiments that align with national strategic goals. Through such efforts, Wuhan is increasingly coupling its academic strength with application arenas – forging a regional innovation pathway that is academically rich, **government-coordinated, and geared toward bridging the gap between lab and market**.

### Policy Recommendations

#### Leverage Optics–Semiconductor Synergies to Build a Photonics Innovation Hub

**Policy Basis and Justification:** Wuhan’s demonstrated strengths in fiber-optic communications and its recent advances in storage chips highlight a unique opportunity at the intersection of photonics and semiconductors. The Regional Differentiation Summary showed that Wuhan’s FOC sector is anchored by world-leading firms (YOFC, FiberHome) and top-tier photonics research at HUST, while the city’s semiconductor push via YMTC has vaulted it into the frontier of memory technology[[4]](https://www.scmp.com/business/article/3239310/chinas-ymtc-makes-worlds-most-advanced-memory-chip-surprise-technology-leap-techinsights-report#:~:text=Yangtze%20Memory%20Technologies%20Co%20,to%20a%20report%20by%20TechInights)[[5]](https://www.scmp.com/business/article/3239310/chinas-ymtc-makes-worlds-most-advanced-memory-chip-surprise-technology-leap-techinsights-report#:~:text=3D%20NAND%20memory%20is%20at,artificial%20intelligence%20and%20machine%20learning). However, these domains currently operate in parallel with limited interaction. Wuhan’s FOC success is rooted in optical hardware and network systems, whereas YMTC’s work in 3D NAND flash is in electronics. Bridging these two could spawn a *new growth avenue – integrated photonic chips and optoelectronic devices*. Such convergence aligns with global technology trends (e.g. silicon photonics, optical interconnects for AI computing) and leverages Wuhan’s path-dependent advantages. The policy basis lies in addressing a **coordination failure**: despite co-location, Wuhan’s optics and semiconductor actors are not yet fully collaborating on photonic integrated circuits or next-gen optical computing. This represents an unrealized innovation potential in the regional system. By intentionally leveraging the synergy between optics (high-speed data transmission, lasers, photodetectors) and electronics (chip fabrication, memory), Wuhan can create a differentiated niche that few other Chinese cities are positioned to fill.

**Mechanism-Level Reasoning:** In Regional Innovation Systems (RIS) theory, innovation often flourishes when diverse knowledge bases are combined, and when **related variety** within a region is harnessed to create new trajectories. Wuhan’s case fits a related-variety strategy: its historically strong “Optics Valley” capabilities can be combined with its emerging semiconductor know-how to stimulate innovation in photonic semiconductors. The mechanism at play is developing a regional **knowledge bridge** to overcome current segmentation. By encouraging joint R&D and cross-sector partnerships, this policy would enhance **knowledge spillovers** between two previously distinct actor networks (photonics researchers and chip engineers). It addresses *absorptive capacity* on both sides: optical firms would gain semiconductor process knowledge, while YMTC and other electronics players could absorb photonics expertise for optical memory or on-chip optical interconnects. This fusion is expected to reduce *systemic failures* where promising research outputs fail to find local industrial uptake. Furthermore, creating a photonics hub aligns with **path creation**: rather than relying solely on the established FOC path or the nascent memory chip path, Wuhan can carve out a new path that multiplies the value of both. This could cement Wuhan’s position in **frontier technologies** beyond the immediate gains in either domain, reinforcing its trajectory toward high-value innovation.

**Implementation Path and Policy Instruments:** A practical implementation would start with establishing a **“Wuhan Photonic Chip Collaborative Innovation Center”** in the Optics Valley zone. This center should be a consortium comprising HUST’s Wuhan National Laboratory for Optoelectronics, Wuhan University’s microelectronics department, YMTC’s R&D division, and local FOC companies (YOFC, FiberHome). The government can provide initial funding and incentives through its special talent and tech funds (as it did with 6G and semiconductor projects[[1]](https://www.yicaiglobal.com/news/wuhans-high-tech-industry-hub-offers-incentives-of-up-to-usd14-million-per-project-to-lure-global-talent#:~:text=The%20fund%20will%20focus%20on,tech%20industry%20hub)) to kick-start joint projects. Key policy instruments include: - **Matching Grants for Joint R&D:** Allocate grants that require participation from at least one optics-focused entity and one semiconductor-focused entity. For example, fund projects on silicon-photonic memory or optical neural network accelerators, with deliverables like prototypes or patents that both sectors can utilize. - **Innovation Voucher System:** Allow smaller firms or research teams in optics to access YMTC’s chip fabrication facilities (and vice versa) via government-subsidized vouchers. This lowers the entry barrier for cross-domain experimentation, especially for startups or university spin-offs wanting to prototype photonic chips. - **Shared Infrastructure:** Invest in a pilot production line or lab for photonic integrated circuits within Optics Valley. Equip it with both photolithography tools and optical testing equipment, making it a shared facility managed by the consortium. Having a common space encourages daily interaction among engineers and researchers from both fields, fostering an interdisciplinary community. - **Policy-driven Networking:** Organize an annual **Optics–Semicon Forum** in Wuhan, inviting national stakeholders in photonic chips. Use this as a platform to showcase Wuhan’s capabilities and attract external collaborations (e.g. CAS Institute of Physics or leading companies like Huawei’s optoelectronic division). The forum can also help identify regulatory or standardization needs that Wuhan can champion, positioning the city as a leader in setting technical standards for integrated photonics.

The implementation should proceed in stages: a two-year pilot phase focusing on a few demonstrator projects (for instance, an optical computing module for data centers), followed by a scale-up phase where successful prototypes get support for commercialization (through Optics Valley incubators or by attracting venture capital). *Institutionally*, the Hubei provincial government and Wuhan city can align this initiative with national programs (e.g. the “Strategic Emerging Industries” plan) to secure additional resources. The presence of a robust industrial base in optics and a major national project in semiconductors means ministries (MIIT, MOST) would likely endorse Wuhan as a photonics hub if local commitment is evident.

**Targeted Scope and Conditions:** This policy targets the **FOC and SC domains** specifically, with spillover benefits to AI and ICV (since both increasingly rely on high-speed chips and sensors). It chiefly involves actors in Wuhan’s Optics Valley (universities, research institutes, large enterprises like YMTC and YOFC, plus SMEs in photonics). The success conditions include: the ability of partners to share intellectual property and work past any institutional silos (requiring clear IP frameworks for joint inventions), and the retention of top talent. The recently announced Optics Valley talent fund (which offers up to CNY100 million to top-tier projects[[11]](https://www.yicaiglobal.com/news/wuhans-high-tech-industry-hub-offers-incentives-of-up-to-usd14-million-per-project-to-lure-global-talent#:~:text=the%20%27Optics%20Valley%20of%20China%2C%27,for%20a%20single%20project)) is a complementary measure that could attract specialists in photonic semiconductors to Wuhan, bolstering human capital. A potential risk is that photonic chip development is capital-intensive and long-gestation. To mitigate this, the policy should integrate **milestone-based reviews** to re-calibrate support. Another condition is national support: since Wuhan’s YMTC is under U.S. sanctions, focusing on photonic chips (less dependent on advanced fabs) could be strategic for self-reliance. If successful, this initiative will yield a cluster of photonic chip startups, new patents (e.g. Wuhan-designed silicon photonics devices), and possibly the first domestically produced photonic co-processor, all of which would solidify Wuhan’s innovation pathway by about 2030. In sum, by institutionalizing optics–semiconductor collaboration, Wuhan can overcome current fragmentation and amplify its regional advantage into a new frontier industry.

#### Expand University–Industry Translational Platforms to Commercialize AI and ICV Technologies

**Policy Basis and Justification:** The analysis of Wuhan revealed a **gap between strong research output and its commercialization**, particularly in **AI and ICV** domains. Wuhan’s universities rank among China’s top in AI publications, yet the city is not nationally recognized as a leading AI industry hub in the way that Beijing or Hangzhou are. Likewise, Wuhan’s bold deployment of autonomous vehicles (500 robotaxis) owes much to external firms (Baidu, DeepBlue) and state-owned enterprises, rather than a homegrown ecosystem of automotive tech startups[[7]](https://www.scmp.com/tech/tech-trends/article/3271143/wuhan-driverless-taxis-offer-peek-future-intracity-transport-china#:~:text=Operating%20under%20the%20brand%20name,and%20shuttles%20in%20the%20city). This points to a missing layer in Wuhan’s innovation system: agile **translational platforms** that can convert academic discoveries into marketable products and viable start-up companies. The policy basis for intervention is the observed **weakness in the innovation value chain**: Wuhan has abundant knowledge creation (academic papers, patents) and large end-users (big industries, public infrastructure), but relatively few intermediaries like incubators with domain focus, technology transfer offices with industry expertise, or venture-backed spin-offs in AI/ICV. Strengthening this middle layer addresses the regional system’s **institutional thinness** in entrepreneurship and mitigates the risk of Wuhan’s research excellence not yielding proportional economic impact. The goal is to prevent promising AI algorithms or intelligent vehicle prototypes developed in Wuhan’s labs from “sitting on the shelf,” and instead facilitate their progression to deployment (perhaps even beyond demonstration fleets into mass adoption).

**Mechanism-Level Reasoning:** In RIS terms, this policy is about improving **network connectivity and reducing fragmentation** between knowledge producers (universities, research institutes) and users (firms, government services) in Wuhan’s AI and ICV sectors. By establishing dedicated translational platforms, the city would tackle what innovation theory calls the *“European Paradox”*-like situation (strong science, weak conversion to innovation). Mechanistically, translational platforms such as joint R&D centers, technology business incubators, or *living labs* create structured environments for **interactive learning** between scientists and engineers on one side and entrepreneurs and end-users on the other. This addresses **systemic failures**: for instance, a coordination failure where universities may not fully understand industry’s problem statements, or where firms are unaware of applicable research emerging locally. Bridging this gap enhances **absorptive capacity** for firms (they get early exposure to new techniques, e.g. HUST’s machine learning advances) and provides researchers with feedback on practical constraints, thus steering research toward market needs. It also fosters a culture of innovation that goes beyond publishing – encouraging faculty and students to take on applied projects or start companies. Another mechanism is mitigating **path dependency**: Wuhan’s legacy industries (optics manufacturing, conventional automotive production) might not naturally diversify into AI or ICV without deliberate interface structures. Translational platforms can inject new knowledge into these traditional sectors, preventing stagnation. By aligning with RIS concepts like the **Triple Helix** (government–industry–academia collaboration), these platforms ensure policy, industry, and science work in tandem on innovation goals such as smart mobility or AI governance tools. Essentially, the policy would create feedback loops that currently might be weak or absent in Wuhan, moving its innovation system from a linear model (lab-to-patent-to-licensing) to a **networked model** where iteration and co-creation are routine.

**Implementation Path and Policy Instruments:** This recommendation can be operationalized through several instruments: - **Joint Innovation Labs:** Establish AI and ICV innovation labs co-funded by city government, local universities (e.g. HUST, Wuhan University of Technology), and leading enterprises (Dongfeng Motor, Hikvision’s Wuhan branch if present, Huawei, etc.). For instance, a *“Wuhan Autonomous Systems Innovation Lab”* could unite an academic AI team with Dongfeng’s engineers to develop and test vehicle perception algorithms. The government can provide matching funds or tax incentives for companies that invest in these labs. Each lab should have clear 2–3 year technology targets (e.g. a vision-based driver assistance module), with IP shared under agreed terms to encourage both academic publication and potential commercialization. - **AI Translational Incubator:** Leverage the Optics Valley’s existing startup incubators to create a specialized **AI Accelerator Program** focused on spinning off research in areas like computer vision, natural language processing, or industrial AI (areas where Wuhan has publication strength). Provide seed funding, office space, and mentorship. Importantly, involve Wuhan’s large state-owned enterprises (SOEs) and municipal agencies as “anchor clients” or beta-testing partners for these start-ups. For example, a start-up emerging from Wuhan University’s AI lab working on smart traffic control could pilot its system in Wuhan’s City Brain platform or with the traffic police department, thereby getting real-world validation and a first customer. This instrument uses **public procurement of innovation** to drive demand for local tech solutions. - **Policy Incentives for Academic Entrepreneurship:** Adjust university performance metrics and city talent programs to reward commercialization efforts. For instance, allow academic entrepreneurs in Wuhan to take **sabbatical leave** to start companies without jeopardizing their positions, and count successful patents/licensing or startup creation as positive criteria in promotions and talent evaluations. Wuhan’s talent policies (often focusing on attracting Nobel laureates or top scholars[[12]](https://www.yicaiglobal.com/news/wuhans-high-tech-industry-hub-offers-incentives-of-up-to-usd14-million-per-project-to-lure-global-talent#:~:text=According%20to%20the%20document%2C%20top,recognized%20as%20future%20tech%20leaders)) can be broadened to include **“entrepreneurial talent”** – those who demonstrate ability to bring research to market. Offer such individuals startup grants or access to low-interest loans. - **Living Labs and Testbeds:** Building on Wuhan’s openness to real-world pilots (as shown by its robotaxi rollout), create formal “living lab” programs in the city. For example, designate a district or a large university campus as a **Smart Mobility Living Lab** where start-ups and research groups can deploy new ICV solutions (shuttles, V2X devices, autonomous delivery robots) under a streamlined approval process and with city logistical support. This could involve installing roadside units, 5G infrastructure, and allowing preferential access or exemptions to speed up trials. The data and feedback from these living labs should loop back to the innovation labs and incubators, creating an iterative environment for product refinement. Essentially, Wuhan can capitalise on its willingness to experiment (e.g. the 35% road coverage for AVs[[6]](https://www.scmp.com/tech/tech-trends/article/3271143/wuhan-driverless-taxis-offer-peek-future-intracity-transport-china#:~:text=While%20cities%20such%20as%20Beijing,a%20pioneer%20of%20the%20technology)) by institutionalizing these experiments as learning platforms open to local innovators.

**Targeted Scope and Conditions:** These measures target **AI and ICV sectors in Wuhan**, focusing on both startups and the innovation activities of established actors. Key participants would be universities (the source of much IP and talent), large enterprises (like Dongfeng for ICV, or even telecom firms for AI applications), and new ventures. Government facilitation is critical, especially Wuhan’s municipal agencies which can provide test environments and initial contracts. For success, one condition is the cultural shift in universities toward pro-entrepreneurship, which may require sustained advocacy from city leaders and early success stories to legitimize the approach. Another condition is engagement of industry: Wuhan’s SOEs must be willing to adopt locally developed AI solutions (over perhaps importing established ones from elsewhere). This can be encouraged by policy (e.g. local content preferences, co-development mandates in procurement). The **risks** include possible failure of start-ups (inevitable in innovation, which calls for a tolerance for failure in policy circles) and the duplication of efforts if coordination is poor (hence, an oversight committee comprising academia, industry, and government should map efforts and avoid redundancy).

To monitor progress, metrics like the number of university spin-off companies per year, the amount of venture capital attracted to Wuhan’s AI/ICV startups, and the adoption rate of local AI solutions in Wuhan’s public services can be used. Over, say, a five-year horizon, Wuhan should aim to incubate dozens of startups (versus only a handful today) and see at least a few scale up to national prominence, akin to how Hangzhou saw AI firms (e.g. SenseTime has a presence there) flourish via strong ecosystems. By implementing these translational platforms, Wuhan will not only strengthen the **actor interactions** in its RIS but also ensure that its heavy investments in frontier research (like the AGI initiative[[9]](https://cset.georgetown.edu/publication/wuhans-ai-development/#:~:text=approaches%20to%20AGI%20that%20involve,surroundings%2C%20learning%20as%20it%20proceeds) or numerous AI papers) yield tangible socio-economic benefits locally. Ultimately, this policy will help Wuhan transition from an innovation system driven “by research and policy” to one balanced “by research, policy, *and entrepreneurship*,” thereby solidifying its comprehensive regional innovation capacity.

## Hangzhou

### Regional Differentiation Summary

Hangzhou’s regional innovation pathway is distinguished by a **dynamic interplay between leading technology firms and academic institutions**, set against a proactive policy backdrop that emphasizes the digital economy. This configuration has made Hangzhou a powerhouse in **artificial intelligence (AI)** and **intelligent & connected vehicles (ICV)**, while also fostering notable (if less dominant) activity in **fiber-optic communications (FOC)** and laying groundwork in **storage chips (SC)**. One of Hangzhou’s defining strengths is the presence of **major private-sector innovation actors** – exemplified by Alibaba Group, which, alongside Zhejiang University (ZJU), forms a twin engine for the city’s AI advancements. Bibliometric data show ZJU produced the largest share of AI publications in Hangzhou (2,800+ papers, 2016–2025), but uniquely, a corporate R&D entity (Alibaba DAMO Academy) also ranks among top contributors with hundreds of high-impact publications. This **academia-industry synergy** is a hallmark of Hangzhou: Alibaba’s research labs collaborate closely with ZJU and other local universities (e.g. on machine learning and data science), blending theoretical and applied AI. The result is that Hangzhou’s AI research profile tilts toward *practical, scalable innovations* – for instance, “attention mechanism” and “LLMs” feature prominently as frontier keywords, mirroring cutting-edge trends that Alibaba and ZJU are jointly exploring (e.g. Alibaba’s development of large language models for its cloud services). Such collaboration has given Hangzhou a nimble capacity to turn frontier research into deployed systems quickly. A case in point is the **City Brain project** launched in Hangzhou in 2016, where Alibaba’s AI was integrated into city governance to optimize traffic flow. This system, initially rolled out to control traffic lights in Hangzhou’s Xiaoshan district, increased vehicle speeds by 15% and soon expanded citywide[[13]](https://en.wikipedia.org/wiki/City_Brain#:~:text=The%20first%20City%20Brain%20system,In%20the%20following). The City Brain exemplifies Hangzhou’s strength in **AI application deployment** – the city leveraged a homegrown corporate technology (Alibaba Cloud’s AI platform) in partnership with government data to achieve a smart city breakthrough that has since been replicated elsewhere[[13]](https://en.wikipedia.org/wiki/City_Brain#:~:text=The%20first%20City%20Brain%20system,In%20the%20following)[[14]](https://en.wikipedia.org/wiki/City_Brain#:~:text=years%2C%20many%20other%20local%20governments,1). The ability to co-create solutions between local government and tech firms (a form of quadruple helix involving the public) underscores Hangzhou’s collaborative innovation culture.

In **ICV**, Hangzhou benefits from a similar private sector drive, anchored by the presence of *Geely Automobile*. Geely, headquartered in Hangzhou, has evolved from a traditional automaker into an innovation-oriented player investing in electric, smart vehicles and autonomous driving. This corporate heft complements academic research from institutions like Hangzhou Dianzi University and ZJU in automotive electronics and control systems. Hangzhou’s ICV publication output, while smaller in volume than its AI output, has grown steadily (from single digits in 2016 to ~194 in 2025) and in 2025 slightly exceeded Wuhan’s. The **technological focus** in Hangzhou’s ICV research leans toward intelligent vehicle software and human-machine interface: frontier terms such as “transformer” (indicating AI models for driving) and “head-up display” appear in Hangzhou’s top ICV keywords. These interests likely reflect cross-pollination from Alibaba’s AI expertise and Geely’s push for smart cockpit features. A standout differentiator for Hangzhou is the city’s policy support for integrating ICV testing with smart city infrastructure. By late 2025, Hangzhou granted Geely an unprecedented **citywide Level 3 autonomous driving test license**, covering all 9,224 km² of the city and 1,500+ km of roads[[15]](https://autonews.gasgoo.com/articles/news/another-automaker-obtains-an-l3-autonomous-driving-road-test-license-2009306621597822977#:~:text=On%20January%207%2C%20Hangzhou%27s%20Bureau,test%20license). This regulatory move – the broadest L3 test permission in China – demonstrates local government’s willingness to remove bottlenecks for innovation and trust in its local champion’s technology[[16]](https://autonews.gasgoo.com/articles/news/another-automaker-obtains-an-l3-autonomous-driving-road-test-license-2009306621597822977#:~:text=The%20approved%20scope%20covers%20all,validation%20of%20autonomous%20driving%20scenarios). It also indicates Hangzhou’s foresight in using its comprehensive urban data (7,000+ intersection traffic feeds were made accessible to Geely’s test vehicles[[17]](https://autonews.gasgoo.com/articles/news/another-automaker-obtains-an-l3-autonomous-driving-road-test-license-2009306621597822977#:~:text=The%20approved%20scope%20covers%20all,validation%20of%20autonomous%20driving%20scenarios)) to facilitate autonomous driving algorithms. In essence, Hangzhou’s ICV pathway is characterized by **strong firm-led innovation (Geely)** supported by open governance, enabling rapid real-world validation of new vehicle technologies. Comparatively, cities like Tianjin or Chengdu have been more restrictive or slower in citywide tests, underlining Hangzhou’s edge in regulatory innovation and public-private coordination.

Hangzhou’s role in **fiber-optic communications** is less historically entrenched than Wuhan’s, yet the city has built capacity through academic excellence and new institutional actors. Zhejiang University leads in FOC research output (over 680 papers) with notable work in optical networking and materials. The city also established the *Zhejiang Lab*, a state-sponsored R&D institute in Hangzhou focusing on network communications and AI. Within a short time, Zhejiang Lab has become a top-5 contributor in Hangzhou’s FOC publications, showing how a purpose-built institute can bolster foundational research in an emerging regional priority. Hangzhou’s FOC innovation shows a pattern of *technological catch-up and diversification*. It may lack native fiber manufacturing giants, but it explores niches like **satellite laser communications and quantum encryption**, as indicated by frontier keywords (e.g. “satellite laser communication”, “quantum random number generator”) appearing in its recent FOC literature. This suggests Hangzhou is carving out specialties at the frontier of communications tech, likely facilitated by the presence of **tech companies like Alibaba and Hikvision** whose businesses rely on advanced communication networks (cloud data centers, IoT security systems). Moreover, Hangzhou’s FOC sector benefits from *regional spillovers*: it’s geographically close to the Yangtze Delta photonics industry, allowing collaboration with nearby Shanghai or Nanjing labs (indeed, we see institutions like Shanghai Jiao Tong University co-authoring papers in Hangzhou’s FOC output). Hangzhou’s differentiation in FOC lies not in volume but in agility – it leverages **collaborative networks** and new research entities to stay relevant in communications technology, aligning with its broader digital economy ecosystem (for instance, robust fiber networks underlie Alibaba’s cloud services and fintech platforms in Hangzhou).

In **storage chips (SC)**, Hangzhou historically was not a major semiconductor center, and the data reflects modest publication counts (peaking at 76 in 2025). However, in recent years, Hangzhou has taken steps to bolster its semiconductor landscape, consistent with Zhejiang province’s initiatives to grow the IC design sector. The city hosts companies like *Silan Microelectronics* (a pioneer in analog ICs) and has seen Alibaba itself foray into chip design (the **Pingtouge** semiconductor division under Alibaba developed AI chips). These corporate moves are complemented by platforms such as the *Zhejiang Integrated Circuits Innovation Platform* led by ZJU[[18]](https://hic.zju.edu.cn/hicenglish/82817/list.htm#:~:text=Zhejiang%20Integrated%20Circuits%20Innovation%20Platform,elite%20industrial%20talent%2C%20it), aiming to provide advanced R&D infrastructure (e.g. 12-inch pilot fab) in Hangzhou. Despite these efforts, Hangzhou’s SC research in the dataset appears to have been classified as almost entirely “frontier” or not captured by the lexicon (no core/basic counts, implying either low volume or focus on advanced topics). This could indicate that Hangzhou’s approach to chips has been to target **cutting-edge niches** (like AI accelerators, IoT chips) rather than mature technologies. Hangzhou’s relative weakness in SC compared to Wuhan (with YMTC) can be partly attributed to path dependence: Hangzhou’s tech ecosystem grew from internet and software, not hardware, so it lacks a deep-rooted semiconductor supply chain. Nonetheless, the city’s innovation strategy is to integrate semiconductors as an enabling layer for its thriving AIoT (AI + IoT) industry. For example, Hikvision (based in Hangzhou) designs its own video processing chips to power its surveillance cameras. Hikvision’s presence and success – it is **one of the world’s largest video surveillance firms**[[19]](https://www.theguardian.com/technology/2023/nov/11/west-bank-palestinians-surveillance-cameras-hikvision#:~:text=Among%20the%20vendors%20behind%20these,of%20the%20Uyghur%20ethnic%20minority) – underscores how Hangzhou’s industry finds ways to innovate in hardware when needed to support its software-driven products. This dynamic, where system integrators drive component innovation, shapes Hangzhou’s SC pathway: instead of standalone chip companies, it’s systems companies (Alibaba, Hikvision, Geely) that commission or develop chips for their products, which in turn spurs local chip design talent and some startups.

Hangzhou’s policy and institutional environment strongly reinforces its innovation trajectory. The city (and Zhejiang province) has a reputation for business-friendly policies, rapid implementation of pilot projects, and *innovation-oriented governance*. One can trace how the local government embraced the concept of a digital economy early on, branding Hangzhou as China’s “E-commerce capital,” and more recently pushing AI solutions in governance (City Brain) and greenlighting expansive autonomous driving trials. The structural characteristic here is **entrepreneurial governance**: local officials often act like venture supporters, willing to take calculated risks on new tech deployments. This stems partly from Zhejiang’s private-sector-led economy, which historically necessitated responsive governance. In comparative perspective, Hangzhou shows what a **mature RIS with balanced actors** looks like: strong firms (Alibaba, Geely, Hikvision), strong universities (ZJU, the growing Westlake University), specialized research institutes (Zhejiang Lab), active local government, and even financial actors (venture capital is abundant due to Alibaba’s spinoffs and wealth creation). This balance yields latent mechanisms of regional differentiation: **reinforced feedback loops** where success begets more investment (e.g. Alibaba’s global success fuels local reinvestment in R&D and philanthropy like Westlake University’s endowment), and **attraction of talent and firms** (Hangzhou draws AI startups and researchers due to the presence of tech giants and quality of life, strengthening clusters). If Wuhan’s pathway is research-and-state driven, and Shenzhen’s (for instance) is market-and-firm driven, Hangzhou stands out for its *hybrid model*, excelling in turning frontier knowledge into real-world systems quickly through tight-knit academia-industry collaboration and progressive policy support.

### Policy Recommendations

#### Deepen Integration of Digital Giants with Local Manufacturing for AIoT Leadership

**Policy Basis and Justification:** Hangzhou’s success in AI and software is evident, driven largely by digital giants like Alibaba and Hikvision, but the Regional Differentiation Summary highlighted a relative shortfall in indigenous semiconductor hardware capabilities and a non-dominant position in traditional manufacturing-based innovation. As global competition in **AIoT (Artificial Intelligence + Internet of Things)** intensifies, Hangzhou must leverage its digital strengths to rejuvenate and upgrade its manufacturing sectors (e.g. smart appliances, connected vehicles, precision equipment) and simultaneously build resilience in hardware technologies. The basis for this policy comes from two observations: (1) Hangzhou’s **storage chip and core hardware contributions are modest** (publications and patents in SC lag behind Wuhan’s, indicating a gap in foundational tech); and (2) Hangzhou’s traditional manufacturing (outside of Geely’s automotive domain) hasn’t been a focal point of its innovation ecosystem, potentially leaving a large part of the regional economy (like machinery, textiles, etc., historically strong in Zhejiang) less touched by AI and IoT advances. Meanwhile, leading digital firms are venturing into hardware (Alibaba’s developing chips, Hikvision building specialized devices), but there is room for a more systemic approach to ensure knowledge transfer and collaboration across the digital-manufacturing divide. In essence, the policy aims to **couple Hangzhou’s prowess in AI/software with its broader industrial base**, to maintain long-term competitiveness and reduce over-reliance on external chip supply chains or single industries. This is justified by RIS theory’s call for stimulating *related variety*: connecting the ICT sector with manufacturing can spawn new activity in **cyber-physical systems, smart manufacturing platforms, and AI-enabled hardware**, aligning with both national strategic industries and boosting local economic complexity.

**Mechanism-Level Reasoning:** The recommended policy works through **strengthening actor networks and technological spillovers**. We propose to formalize partnerships between Hangzhou’s **digital champions (Alibaba, Ant Group, Hikvision, etc.) and mid-sized manufacturers** in the region, using these large firms as catalysts to upgrade traditional industries. Mechanistically, this taps into **external economies of scope**: big tech firms possess advanced capabilities in cloud computing, AI algorithms, and system integration, which, if shared with or embedded in manufacturing processes, can yield leaps in productivity and innovation (e.g. smart factories, predictive maintenance, AI-designed products). The policy also addresses **coordination failures**: SMEs in manufacturing might lack access to cutting-edge AI, while tech firms might not fully understand manufacturing pain points. A city-facilitated integration program would coordinate these sides, effectively acting as an **innovation broker** to overcome market frictions. From a RIS perspective, this fosters a *Triple Helix dynamic*: government policy nudges industry-academia cooperation, in this case adding a twist by pairing different industry domains (ICT and manufacturing). It also enhances **system synergy** – connecting currently siloed sub-systems (the digitally-oriented cluster and the manufacturing cluster) so that the region’s innovation is more holistic. Over time, this integration can lead to **new path development**, such as Hangzhou becoming a leader in AIoT solutions, where it designs and produces both the software and specialized hardware for smart devices. The reasoning aligns with the concept of **absorptive capacity** at the firm level: manufacturing firms will develop greater capacity to absorb digital tech, while digital firms will learn to absorb domain knowledge of physical industries, thus broadening the knowledge base of the region.

**Implementation Path and Policy Instruments:** A multi-pronged approach should be used: - **AIoT Industry Alliance:** Form a Hangzhou *AIoT Collaborative Alliance* under the auspices of the municipal government or Zhejiang province. This alliance would include major tech firms (Alibaba Cloud, Hikvision, Dahua, Geely’s tech arm), manufacturing companies from key sectors (textile machinery, electronics, automotive parts, etc.), universities (ZJU, Westlake for research input on sensor tech, robotics, etc.), and service providers (telecom operators for 5G). The alliance’s mandate is to identify integration projects – for example, applying computer vision and IoT sensors in textile mills to automate quality control, or using Alibaba’s cloud and AI on a factory floor to optimize energy use. - **Demonstration Smart Factory Program:** The government can fund **10–20 pilot “smart factory” upgrades** in traditional industries across Hangzhou and neighboring cities. Each pilot pairs a tech company with a factory. For instance, Alibaba Cloud could work with a household appliance manufacturer in Hangzhou to deploy an **Industrial Internet platform** that connects machines and applies AI to production scheduling. Performance improvements (quality yield, cost reduction) can be measured and publicized. These pilots act as proof of concept, and the city can offer tax breaks or subsidies (e.g. covering 30-50% of the project cost) to incentivize participation. The focus must be on replicable solutions that can then be rolled out to hundreds of SMEs, turning Hangzhou into a model for digital transformation of industry. - **Embedded Engineers and Exchange Programs:** Encourage a two-way exchange of talent: allow engineers from big tech to do residencies in manufacturing firms and vice versa. The city could sponsor an “**AI Talent in Industry**” program where, say, a data scientist from Ant Group spends 6 months at a manufacturing company to implement an AI solution, funded partly by a government stipend. Conversely, domain experts from manufacturing could spend time at tech companies or ZJU’s engineering labs to learn about latest digital tech. This human-capital instrument builds trust and understanding across sectors, which is crucial for sustained integration. - **Local Supply Chain Development for Hardware:** Given Hangzhou’s noted weakness in core hardware (chips, specialized sensors), as part of integration the policy should bolster **joint ventures or supplier development** in these areas. For instance, if Hikvision needs advanced imaging chips, encourage it to partner with or incubate a fabless semiconductor startup locally (with supportive financing from provincial funds). Hangzhou’s government can use tools like **co-investment funds** to attract needed hardware firms or to help local ones scale. The idea is to ensure that as Hangzhou companies deploy AIoT, they have a local or at least domestic source for critical components, enhancing resilience and keeping value creation within the region. - **Standards and Platforms:** Promote Hangzhou-led standards in AIoT interoperability. Through the alliance, push for technical standards or open platforms (for smart home devices, V2X communications, etc.) that favor solutions developed in Hangzhou. This would lock-in customers to local ecosystems (for example, many IoT devices running on Alibaba’s AliOS or using Hikvision’s protocols) and establish Hangzhou as a **reference city** for AIoT. It could also involve creating a **city-level data exchange** where anonymized industrial data can be shared for AI model training – something Hangzhou’s government can coordinate given its digital governance experience.

**Targeted Scope and Conditions:** This policy chiefly targets **the intersection of AI/software sector and manufacturing sector** in Hangzhou and the broader Zhejiang region. It involves large firms (which have the resources and tech), SMEs (which need tech upgrading), academia (for R&D support), and government (as facilitator and co-funder). One condition for success is **willingness of big tech firms to participate earnestly**, not just for PR. The incentives must be structured so that they see value – for example, new market opportunities in selling industrial AI solutions. Given Alibaba’s push into cloud and IoT, and Hikvision’s interest in factory automation cameras, alignment is likely if approached well. Another condition is ensuring intellectual property agreements that protect both sides, so collaboration is not seen as risking trade secrets (this can be managed through NDAs and alliance guidelines).

**Potential Risks/Limitations:** A risk is that tech solutions might be applied as one-off pilots but not scaled due to organizational inertia in traditional firms or insufficient ROI. Mitigation: choose pilot sites that have committed leadership and are mid-to-large scale (so they can implement and sustain changes), and involve them in solution design. Another risk is overlap with national programs (like MIIT’s intelligent manufacturing pilots) – this can be turned into a positive if Hangzhou secures national “smart manufacturing demonstration city” status, bringing in additional resources.

In terms of outcomes, over a 3-5 year period we’d expect to see measurable increases in productivity or innovation metrics in participating manufacturing firms (e.g. patent filings by these firms, reduction in defect rates, etc.), as well as the creation of new products (e.g. Hangzhou-branded smart machinery) that give these firms a competitive edge. Hangzhou could also see growth in its hardware sector – if integration projects succeed, they will generate demand for new types of sensors, controllers, and chips, which local entrepreneurs or incoming firms can fulfill, gradually balancing the software-hardware mix. Ultimately, this policy secures Hangzhou’s position as a **leader in AIoT** by ensuring that its famed digital economy is interwoven with a cutting-edge manufacturing base, thus future-proofing the region’s innovation ecosystem against shifts in technology trends or supply chain disruptions.

#### Foster Frontier Research and Startup Ecosystems in Semiconductors and Photonics

**Policy Basis and Justification:** While Hangzhou has excelled in software-driven innovation, the Summary identified its relative weakness in foundational high-tech hardware domains, particularly semiconductors (storage chips) and, to a lesser extent, photonics (FOC). Hangzhou’s SC outputs are minimal and not well-represented in the dataset (indicating a lack of critical mass or lexicon match), and its FOC research, though active academically, lacks a strong industrial counterpart (no major fiber/cable manufacturing like Wuhan’s YOFC). Given the rising strategic importance of **technological self-reliance** in semiconductors and the convergence of photonics with AI (e.g. optical computing, LIDAR in vehicles), Hangzhou risks falling behind in next-generation hardware innovation if it relies solely on imported or externally developed tech. The policy basis is thus to **strengthen Hangzhou’s capacity in core enabling technologies** by building a pipeline from frontier research to commercialization specifically in semiconductors and photonic technologies. This also leverages recent investments like the Zhejiang IC Innovation Platform and Westlake University’s advanced science programs. A specific impetus is Alibaba’s and others’ growing need for custom chips (AI chips, security chips) – rather than having those designed or fabricated elsewhere (often abroad), a local ecosystem could serve those needs, creating a symbiotic relation between Hangzhou’s hardware and software sectors. Moreover, focusing on photonics aligns with Hangzhou’s ICV and surveillance strengths (since both involve LIDAR, cameras, optical sensors). There is an opportunity to differentiate Hangzhou through a **“silicon + light” innovation agenda**: combining semiconductor microelectronics and optical technologies to produce cutting-edge components (like AI accelerators or optical network devices). This aligns with Hangzhou’s track record of targeting frontier niches (e.g. quantum communications research at ZJU). Importantly, a policy push here addresses an **imbalance in the RIS**: currently actor composition is skewed towards software/internet; adding robust hardware players (startups, fabs, labs) will make the system more balanced and resilient.

**Mechanism-Level Reasoning:** The policy would employ mechanisms of **strategic niche management and entrepreneurial support within an RIS context**. Essentially, Hangzhou needs to create *protected spaces* or dedicated resources for semiconductor and photonics innovation, allowing them to incubate despite currently being weaker links in the region. This involves cultivating specialized human capital (chip designers, photonics engineers) and networks for them to interact with investors and customers. A key mechanism is to stimulate **spin-offs and startups** in these domains, because Hangzhou’s corporate structure currently lacks established giants in chips/photonics (unlike Nanjing’s Huada or Wuhan’s YOFC). By focusing on startups, Hangzhou can inject dynamism and eventually grow its own champions. This is consistent with **RIS evolution theory**, where new niches often arise from university research or from diversification of existing firms. Another mechanism is the establishment of **institutional thickness** around these domains: e.g. dedicated innovation centers, incubators, and events that create a community and collective vision for local semiconductor and photonics development. This addresses **fragmentation failures** by bringing together actors who might otherwise not find each other (e.g. a PhD in optoelectronics at ZJU and an angel investor interested in deep tech). Additionally, by linking semiconductor and photonics research to Hangzhou’s existing industries (AI, surveillance, ICV), the policy will ensure **market pull** for these new ventures, improving their chances of survival (a form of enhancing systemic demand-side readiness). The policy implicitly uses **cluster theory**: building a cluster around semiconductors/photonics in a region that already has complementary industries yields positive externalities (like easier hiring, shared suppliers, specialized legal and consulting services for tech). Essentially, this recommendation aims to embed new high-tech cluster elements into Hangzhou’s RIS, thereby expanding its knowledge base breadth (currently strong in software, it would become strong in hardware too).

**Implementation Path and Policy Instruments:** Key steps and instruments include: - **Frontier Technology Research Center:** Establish a *Hangzhou Frontier Chip and Photonics Research Center* as a joint initiative of Westlake University, Zhejiang University, and leading industry players (Alibaba DAMO’s chip lab, Geely’s semiconductor unit, etc.). Equip it with state-of-the-art facilities for chip prototyping (like a cleanroom for photonic chip fabrication, a semiconductor characterization lab). The government can fund initial capital expenditures and operate it as an open platform for researchers and startups to use at low cost. Within this center, create special research programs targeting, say, *AI chips for cloud computing*, *edge IoT chips*, *LIDAR and optical sensors*, *quantum communication devices*. The presence of such an institution provides a focal point for talent and projects, akin to how Zhejiang Lab bolstered FOC/AI; it also signals long-term commitment. - **Semiconductor Startup Accelerator:** Launch an accelerator program specifically for semiconductor and photonics startups – which typically require more capital and longer development cycles than internet startups. Offer selected teams seed funding (from a dedicated city or provincial fund), free/discounted access to the above research center’s facilities, and mentorship from industry veterans (who could be recruited from the well-established chip hubs like Shanghai or overseas). Also, given the heavy investment needed for hardware, facilitate connections to national funds (e.g. China’s Big Fund) or corporate venture arms (like Alibaba’s investment vehicles) for follow-on funding. Hangzhou could differentiate by being one of the few places strongly supporting *fabless chip design startups* and *photonics startups* outside the traditional hubs. Over time, aim to incubate dozens of startups; even if a few succeed, they can anchor a growing cluster. - **Talent Attraction and Training:** Modify talent incentive programs to attract chip design experts and optical engineers. For instance, include categories for “Semiconductor Technology Leaders” in Hangzhou’s Peacock or Phoenix talent plans, offering housing subsidies, research grants, etc., to engineers or scientists who relocate. Collaborate with local universities to expand programs in microelectronics and photonic engineering (perhaps a joint college between ZJU and Westlake focusing on photonic integrated circuits). Also, implement a program to send promising local students or young engineers to spend time in leading semiconductor companies (like TSMC in Nanjing, or research orgs in Shanghai/Shenzhen) for training, under the agreement they return to Hangzhou ventures or labs. - **Innovation Procurement and Testbeds:** Encourage Hangzhou’s big companies and government units to be early adopters of local semiconductor/photonics innovations. For example, if a Hangzhou startup develops an AI accelerator chip or a new camera sensor, Alibaba Cloud or Hikvision could pilot it in their products, or the city could use it in public projects (smart city devices, etc.). This “lead customer” approach can be formalized via MOUs in the alliance and possibly supported by government subsidies (to offset any risk/cost penalty of using unproven local tech). Furthermore, set up testbeds where these new chips/devices can be tested in real conditions – e.g. integrate a photonic sensor on traffic infrastructure in Hangzhou to gather performance data. Fast feedback will help startups iterate and demonstrate viability to broader markets. - **Financial and Regulatory Support:** Create conditions to ease the path of hardware startups – these might need longer “runways”. Offer tax breaks or rent-free periods in dedicated innovation parks for semiconductors/photonics. One idea is a **“Chip Harbor”** within Hangzhou’s Xiasha or Yuhang tech zone, clustering small design firms, with shared office and lab facilities. Also, coordinate with national regulatory bodies to ensure any needed certifications (for comms equipment, automotive chips, etc.) can be fast-tracked for Hangzhou pilots, so startups aren’t bogged down waiting for approvals.

**Targeted Scope and Conditions:** The focus is squarely on **semiconductors and photonic technologies** in Hangzhou, aiming to strengthen the *Foundational/Core Technology* layer of its innovation hierarchy. The stakeholders are universities (for research talent), startups and entrepreneurs (for agile innovation), large firms (as mentors, investors, or first customers), and government (as enabler). Key conditions include sustained funding – hardware initiatives can be expensive, so commitment for at least 5-10 years is needed. Hangzhou’s prosperous financial status and provincial backing (Zhejiang’s strong economy) make this feasible if prioritized. Another condition is building a culture that celebrates deep tech entrepreneurship; Hangzhou has a history of e-commerce startup success, which may not directly translate to semiconductor startup mindset. Thus, showcasing success stories (perhaps luring a star entrepreneur to start a chip venture in Hangzhou) early on can help.

One limitation to acknowledge is that Hangzhou is not starting from a position of strength in these fields, so initial progress might be slow. Patience is essential; metrics such as number of patents, prototypes, or startups may be small at first. However, qualitatively, one should see increasing participation of Hangzhou teams in national semiconductor initiatives, more co-publications in top journals like IEEE Electron Device Letters from Hangzhou labs, etc. A risk is competition and overlap with nearby hubs (Shanghai’s big national fabs or Hefei’s quantum tech focus). To mitigate, Hangzhou should specialize in areas that complement its industries: e.g. *edge AI chips* (for IoT, aligning with Alibaba Cloud and Hikvision’s needs) or *automotive semiconductors* (aligning with Geely), and *optical interconnects or sensors* (aligning with Hikvision/ICV). Being strategy-driven in specialization will help attract targeted support and not just replicate what others do.

If executed well, within a decade Hangzhou could see the rise of at least a few notable hardware firms – say a domestically leading fabless chip designer originating in Hangzhou for IoT chips, or a photonics unicorn making novel sensors. These would feed back into the local RIS, hiring local graduates, working with local industry, thus deepening the region’s innovation capacity. This policy, alongside the earlier one, ensures Hangzhou’s **innovation ecosystem remains balanced and cutting-edge**, spanning from software to hardware, and secures its competitiveness in an era where **the fusion of software and hardware (“hard tech”) is increasingly key** to sustained technological leadership.

## Chengdu

### Regional Differentiation Summary

Chengdu’s innovation trajectory is characterized by a **strong academic and research base**, a diverse array of specialized universities and institutes, and a growing, though still maturing, high-tech industry scene. Within the context of the four focal industries—AI, FOC, ICV, and SC—Chengdu exhibits notable strengths in **artificial intelligence (AI)** and **fiber-optic communications (FOC)**, leveraging key national universities, while it shows a more modest profile in **intelligent vehicles (ICV)** and **storage chips (SC)** with recent momentum building through targeted initiatives. A distinctive feature of Chengdu’s RIS is the presence of multiple prominent universities each contributing in different domains: the University of Electronic Science & Technology of China (UESTC) excels in electronics and ICT, Sichuan University brings strengths in computer science and biomedical applications, and Southwest Jiaotong University contributes to transport and rail-related tech. These institutions collectively propelled Chengdu’s **AI publication output** to high levels—by 2025 Chengdu-AI publications (1,852) were nearly on par with Wuhan’s—indicating a vibrant research community. UESTC alone produced over 2,700 AI-related papers (2016–2025) and garnered 74,000+ citations, evidencing **deep expertise in fields like computer vision, signal processing, and machine learning**. This academic heft translates into certain niche strengths: for example, Chengdu’s AI researchers have been active in areas such as **financial AI** (Southwestern University of Finance & Economics appears among top contributors, reflecting fintech AI research) and **educational or social AI** (with normal universities and others involved). The **collaboration pattern** in Chengdu’s AI landscape is notably inter-institutional and regional—CAS institutes and even Beijing-based collaborators (like BUPT in FOC, Tsinghua in AI) appear in co-authorship, suggesting Chengdu’s researchers are well-networked nationally. However, one relative weakness is the scarcity of local private tech giants driving AI; unlike Hangzhou or even Wuhan, Chengdu historically lacked headquarters of large internet companies. This is partly offset in recent years by the entry of firms like Huawei (which built a big data/AICenter in Chengdu Hi-Tech Zone[[20]](https://www.prnewswire.com/news-releases/chengdu-making-strong-push-to-foster-next-gen-ai-technology-301181080.html#:~:text=In%20September%2C%20Chinese%20communication%20technology,9%20billion%20yuan)[[21]](https://www.prnewswire.com/news-releases/chengdu-making-strong-push-to-foster-next-gen-ai-technology-301181080.html#:~:text=The%20center%20will%20bring%20Huawei%27s,construction%2C%20the%20telecoms%20giant%20said)) and partnerships with Baidu (robotaxi deployments in Chengdu[[22]](https://www.prnewswire.com/news-releases/chengdu-making-strong-push-to-foster-next-gen-ai-technology-301181080.html#:~:text=construction%2C%20the%20telecoms%20giant%20said)). These moves have helped translate research strength into application: for instance, Huawei’s AI research center and Chengdu’s new supercomputing center (the first in West China) provide critical infrastructure that bolsters local AI development[[23]](https://www.prnewswire.com/news-releases/chengdu-making-strong-push-to-foster-next-gen-ai-technology-301181080.html#:~:text=Image%3A%20Chengdu%20Supercomputing%20Center%2C%20the,started%20trial%20operations%20in%20September)[[24]](https://www.prnewswire.com/news-releases/chengdu-making-strong-push-to-foster-next-gen-ai-technology-301181080.html#:~:text=Chengdu%20Supercomputing%20Center%2C%20the%20first,industry%20growth%20in%20the%20region). Chengdu’s innovation system thus is evolving from an academic-led model to a more balanced one as big tech involvement increases.

In **fiber-optic communications (FOC)**, Chengdu similarly leans on its academic foundations. UESTC, historically one of China’s premier telecom and optical engineering schools, anchors the city’s FOC endeavors with over 500 publications (2016–2025). These often focus on optical network technologies, photonic devices, and fiber sensing, aligning with UESTC’s traditional strengths. Secondary players like Southwest Jiaotong University contribute in niche areas such as optical communications for rail or rugged environments. Chengdu’s FOC research, while significant, is somewhat less diversified in actor types: top contributors are mostly universities and CAS institutes, with little representation from industry. The city does host some defense-oriented and state electronics enterprises (e.g. CETC has presence in Chengdu), which likely conduct optical communications R&D, but these do not show up in publication data (perhaps due to secrecy or non-academic channels). Instead, Chengdu’s open literature suggests a push toward **frontier optical topics** similar to elsewhere – “photodetector” is the leading frontier keyword, and interest in integrated sensing & comm appears as well. This implies Chengdu’s researchers are active in developing advanced optical components and exploring future network paradigms (like sensing-communication integration relevant for 6G). Relative to Wuhan, Chengdu’s FOC pathway is more academically driven and has not benefited from a large industrial cluster; however, this also means Chengdu’s knowledge production is broad (collaborations with national labs in Beijing, Hong Kong CityU, etc., are seen) and could be readily applied if industrial catalyzers emerge. Recognizing this, Sichuan’s government has aimed to strengthen “electronic information” as a pillar industry, which includes photonics. Indeed, Chengdu’s Hi-Tech Zone has attracted companies in optical modules and lasers, albeit at a smaller scale than Wuhan’s Optics Valley. The regional differentiation here lies in **Chengdu’s potential for convergence of electronics and optics** – UESTC’s electronics background can complement photonics to yield optoelectronic innovations. For example, Chengdu could innovate in **optical interconnects for computing** or special fiber sensors for oil/gas (leveraging Southwest Petroleum University’s involvement). These niche directions are emerging and reflect the region’s strategy to use its broad S&T base to find unique applications of FOC in local contexts (like energy, as indicated by petroleum university participation in AI which could extend to sensing).

Chengdu’s performance in **intelligent and connected vehicles (ICV)** has been moderate but is accelerating due to policy focus and the city’s strength in electronics and AI. In publication terms, Chengdu’s ICV output (141 papers in 2025) is behind Wuhan and Hangzhou, but the city has strong relevant assets: it’s a major automotive manufacturing hub in West China (with plants for Volkswagen, Toyota, Volvo/Polestar via Geely’s ownership) and houses key research institutes like the Automotive Engineering Research Institute of Sichuan. Historically, Chengdu wasn’t a leader in automotive R&D, but it’s now capitalizing on national ICV initiatives. The city was designated a pilot for smart city and vehicle infrastructure cooperation; for instance, Chengdu has tested Baidu Apollo robotaxis since 2020[[22]](https://www.prnewswire.com/news-releases/chengdu-making-strong-push-to-foster-next-gen-ai-technology-301181080.html#:~:text=construction%2C%20the%20telecoms%20giant%20said). The region’s universities contribute ICV-related expertise: UESTC in sensor fusion and automotive electronics, Southwest Jiaotong in traffic systems and rail-vehicle automation (some tech cross-over), and smaller institutes focusing on vehicle engineering (e.g., Xihua University in mechatronics). The keywords data shows Chengdu’s ICV frontier topics align with national trends—“transformer” models for driving AI, “LiDAR (laser radar)” for perception, and even automotive-specific tech like “FMCW” (a radar technology) show up in Chengdu’s list. This suggests Chengdu researchers are investigating advanced driver algorithms and sensors. What differentiates Chengdu here is the **concerted effort to integrate local AI strengths into automotive applications**: the municipal government and Chengdu Hi-Tech Industrial Development Zone have partnered with companies (e.g. Huawei, Baidu) to build infrastructure (like an AI computing center and 5G test roads). Notably, Huawei’s aforementioned AI computing center in Chengdu (300 PFlops)[[25]](https://www.scmp.com/news/china/politics/article/3298826/chinas-tianjin-city-embraces-deepseek-part-rush-embrace-domestic-ai-industry#:~:text=The%20Tianjin%20AI%20Computing%20Centre%2C,DeepSeek%E2%80%99s%20models%2C%20the%20report%20said)[[26]](https://www.scmp.com/news/china/politics/article/3298826/chinas-tianjin-city-embraces-deepseek-part-rush-embrace-domestic-ai-industry#:~:text=second) likely supports autonomous driving R&D by providing powerful model training capabilities. Additionally, Chengdu’s push to be a **“national pilot zone for next-gen AI”** included smart transportation as a key area[[27]](https://www.prnewswire.com/news-releases/chengdu-making-strong-push-to-foster-next-gen-ai-technology-301181080.html#:~:text=CHENGDU%2C%20China%2C%20Nov,generation%20artificial%20intelligence)[[28]](https://www.prnewswire.com/news-releases/chengdu-making-strong-push-to-foster-next-gen-ai-technology-301181080.html#:~:text=According%20to%20the%20plan%2C%20Chengdu,hub%20and%20several%20industrial%20parks). Through these efforts, Chengdu’s ICV pathway appears to be one of **rapid catch-up**: leveraging external tech (Huawei/Baidu) combined with local talent to build an ecosystem. Compared to Tianjin, for example, Chengdu has been more successful in attracting and embedding top-tier tech firms to jumpstart its ICV and AI efforts. Yet, relative to Hangzhou or Wuhan, Chengdu still lacks a flagship indigenous company in autonomous driving; much of its progress relies on collaborations and branch operations of outside firms. The structural characteristic here is an **innovation system in transition**: moving from being academically rich but enterprise-poor, toward spawning local startups or subsidiaries in ICV (for instance, there are reports of Chengdu fostering UAV and vehicle AI startups, often by UESTC alumni). The latent mechanism is that **Chengdu’s comprehensive education system supplies talent** (the city is known to export graduates to coastal tech companies); now policies aim to retain that talent by creating local high-tech opportunities.

For **storage chips (SC)**, Chengdu historically played a manufacturing role (Intel has had a chip packaging & testing plant in Chengdu since 2005[[29]](https://finance.yahoo.com/news/intel-invests-us-300-million-093000185.html#:~:text=Intel%20invests%20US%24300%20million%20in,commitment%20to%20the%20mainland%20market)), but less so a design or innovation leadership role. The data confirms Chengdu’s SC publications are low (hovering in the 30s annually) and largely focused on frontier aspects when present (e.g. one mention of “non-volatile memory” as a frontier keyword). However, behind these figures, Chengdu has been quietly building a semiconductor industry, particularly around **power electronics and third-generation semiconductors**. For example, companies like Chengdu Analog Circuit (APS) focus on power chips[[30]](https://en.lrc.cn/about/group.html#:~:text=Chengdu%20Advanced%20Power%20Semiconductor%20Co,production%20and%20sales%20of), and the city has initiatives in SiC (silicon carbide) devices for electric vehicles[[31]](http://gcsemi.cn/en/#:~:text=Products%20,power%20semiconductor%20applications%20in%20China). So, while Chengdu isn’t producing many academic papers on memory or logic chips, it is developing industrial capacity in chips relevant to its regional strengths (energy, automotive). Another development is that **Chengdu is among the top cities for IC design startups** in China’s west. This can be attributed to lower costs and good talent supply from UESTC’s microelectronics program. The city’s innovation approach in SC might thus be more industry-driven (through manufacturing and startups) than academic. Comparatively, Tianjin has almost no presence in SC innovation, and Hangzhou’s is minimal; Chengdu at least has a foothold via Intel’s presence (which undoubtedly helped train local workforce) and new ventures. A structural factor is Sichuan’s policy to make Chengdu–Chongqing region a semiconductor hub to support western China; for instance, local governments have set up funds to entice chip projects. Chengdu’s budding SC cluster is not at the frontier of memory like Wuhan, but it targets *“basic and core”* technologies like analog chips, which might not reflect in frontier keyword counts but are crucial for underpinning tech industries. This balanced approach could be seen as a differentiation: rather than chasing the exact frontiers, Chengdu focuses on **complementary chip technologies** (power, analog, packaging) that serve its economic environment (lots of manufacturing, growing EV sector). Over time, if these efforts bear fruit, Chengdu could host a robust mid-tech semiconductor cluster, which in turn supports ICV (through automotive chips) and AI (through local supply of certain components).

Finally, Chengdu’s **policy and institutional environment** is strongly oriented toward *inclusive innovation growth* across the region. As the capital of western China’s Sichuan province, Chengdu is often positioned as a counterbalance to the coastal innovation centers. The city’s strategies often emphasize improving the ecosystem: building **high-tech zones** (the Chengdu Hi-Tech Industrial Development Zone and Tianfu New Area), creating better conditions for startups, and leveraging national programs (like the Western China Silicon Valley initiative). The **underlying logic** of Chengdu’s differentiation is that it had to overcome its lack of major corporate HQs by maximizing its human capital and by attracting external collaborators. This it did by offering a relatively lower-cost environment, a strong talent pool, and government support. There’s evidence of **RIS maturation**: the presence of Huawei’s and Baidu’s projects indicates Chengdu can now draw investment by virtue of its growing market and talent magnet effect[[20]](https://www.prnewswire.com/news-releases/chengdu-making-strong-push-to-foster-next-gen-ai-technology-301181080.html#:~:text=In%20September%2C%20Chinese%20communication%20technology,9%20billion%20yuan)[[22]](https://www.prnewswire.com/news-releases/chengdu-making-strong-push-to-foster-next-gen-ai-technology-301181080.html#:~:text=construction%2C%20the%20telecoms%20giant%20said). Another mechanism at play is **path dependence tempered by diversification**: historically, Chengdu’s defense electronics roots (with many military institutes) laid groundwork in electronics and optical tech, which now are being diversified into civilian AI and communications applications. This path extension is facilitated by national defense-civil fusion policies that Chengdu actively implements, given its defense industry base in Mianyang and Chengdu. A concrete example is the embodied AI robot testing ground in Mianyang, Sichuan[[32]](https://en.people.cn/n3/2025/0722/c90000-20343456.html#:~:text=Based%20in%20the%20city%20of,setting%20and%20commercialization)[[33]](https://en.people.cn/n3/2025/0722/c90000-20343456.html#:~:text=The%20project%2C%20officially%20launched%20on,department%20told%20Xinhua%20on%20Tuesday), showing how provincial initiatives are trying to pivot defense tech expertise (robots, AI) into civil use. Chengdu benefits from this as the region’s innovation network hub.

In summary, Chengdu’s regional innovation pathway is one of **solid science and engineering capabilities gradually translating into industrial innovation**, with significant recent accelerations due to strategic partnerships and policy focus. Its actor configuration—multi-polar academic centers, nascent local enterprises, and increasing involvement of national tech companies—differs from Wuhan’s (more centralized around a few key unis and SOEs) and Hangzhou’s (dominated by big private firms). Chengdu’s trajectory is creating a distinctive model: a **regionally collaborative, broad-based innovation system** that is beginning to yield specialized high-tech outcomes (like fintech AI, power chips for EVs, etc.), aligning with both local and national development objectives to uplift western China’s tech profile.

### Policy Recommendations

#### Cultivate a Homegrown High-Tech Enterprise Ecosystem to Complement Research Strengths

**Policy Basis and Justification:** The Regional Differentiation Summary made clear that Chengdu’s innovation system, while strong in knowledge generation, lacks a proportional representation of **locally grown high-tech enterprises**—especially in the AI, ICV, and semiconductor sectors. Chengdu’s top contributors in publications are universities and external firms, reflecting that much innovation activity either resides in academia or comes via branches of non-local companies (e.g. Huawei, Baidu) rather than through indigenous firms. This points to a systemic bottleneck: Chengdu has not yet fully converted its rich talent and research outputs into a robust pipeline of **local start-ups and scale-ups**. Nurturing homegrown enterprises is crucial for sustainable innovation because they are more likely to embed in the local economy, create specialized supply chains, and reinvest in the region. For instance, despite UESTC’s prolific output in electronic and AI research, few nationally renowned tech companies have originated from Chengdu in these domains. The policy basis for intervention, therefore, is to address this gap by fostering an **entrepreneurial ecosystem** that can retain Chengdu’s talent and intellectual property locally. This is justified by noticing phenomena such as brain drain (Chengdu-trained engineers moving to Shenzhen or Beijing for start-ups), and by comparison to peers: Hangzhou and Wuhan both have seen stronger emergence of local champions (Alibaba/Geely in Hangzhou, YOFC/HiSilicon’s subsidiary in Wuhan) which feed back into their RIS. Chengdu’s push to create an enterprise ecosystem would ensure its heavy investments in education and research translate to local economic growth and innovation resilience. Specifically, this means creating more **Chengdu-born companies in AI software, AI hardware, optical tech, and automotive tech** that can join or lead clusters, alongside existing SOEs and MNC R&D centers. Enhancing the enterprise base also mitigates reliance on external actors who might reallocate if priorities shift.

**Mechanism-Level Reasoning:** The policy’s objective aligns with building **system connectivity and entrepreneurship support** in RIS terms. Mechanisms include strengthening **networks between academia and industry**, providing platforms for knowledge spillover from universities into new firms, and offering financial and institutional support for start-ups. A key concept here is improving **regional absorptive capacity** not just for established firms (as earlier recommendations for other cities often target) but for *new firm formation*. By equipping prospective entrepreneurs with resources and reducing barriers to entry, the region can better absorb the intellectual output of its research institutions. This will address what innovation theory calls **structural holes** – currently, there may be insufficient intermediaries like incubators, mentorship networks, and venture capital in Chengdu linking researchers to markets. Filling these holes by orchestrating interactions (pitch days, industry-university forums) is vital. Moreover, encouraging local enterprise formation deals with **path dependency** that has historically funneled talent into government or large SOE jobs; a more vibrant start-up scene can alter career expectations and unleash creative potential previously untapped. The reasoning also draws on **cluster dynamics**: a cluster is more robust if it has numerous SMEs and startups complementing big anchors. At present, Chengdu’s tech cluster leans on a few big entities (often non-local HQ’d). By seeding many local enterprises, you create a self-reinforcing mechanism: some will succeed and become anchors themselves, others will provide specialized services, and all together they form a **thicker institutional milieu** supporting innovation (through shared labor pools, demonstration effects, etc.). This policy thus operationalizes the **entrepreneurial discovery process** within Chengdu’s RIS: enabling entrepreneurs to find new commercial opportunities from the region’s knowledge base (like novel AI applications in local industries, or new components needed by manufacturing in Sichuan).

**Implementation Path and Policy Instruments:** - **Dedicated Tech Incubators and Accelerators:** While Chengdu already has high-tech zones, we propose establishing **thematic incubators** for each key domain—AI, ICV, semiconductors, and photonics—within those zones. For example, set up an “AI & Big Data Incubator” near the Chengdu Supercomputing Center, an “Autonomous Driving and Robotics Accelerator” in Tianfu New Area, and a “Chip Design Startup Incubator” perhaps in cooperation with UESTC’s campus. Each incubator should offer subsidized office/lab space, shared equipment (like cloud computing credits for AI, or EDA software for chip design), and professional services (legal, accounting, marketing) to reduce startup overhead. They should also have on-site mentorship teams drawn from successful entrepreneurs (maybe attract some returnees originally from Sichuan who founded companies on the coast) and retired executives from Huawei, etc. - **University-Linked Entrepreneurship Programs:** Encourage universities in Chengdu to create **innovation and entrepreneurship centers** that actively funnel students and faculty towards startups. For instance, UESTC can have a fund and incubator for student startups in electronics/AI, Sichuan University for biomedical AI or fintech ideas, etc. These centers would host hackathons, business plan competitions, and pre-seed funding rounds. The city can provide matching funds to universities that set up such programs and demonstrate outcomes (like a number of startups created per year). In addition, implement a policy that makes it easier for professors to take leave to start companies or for graduate students to pause studies to pursue a venture, thus reducing institutional rigidity. - **Financial Incentives and Venture Capital Mobilization:** Chengdu’s government could establish a **Chengdu Innovation Venture Fund** that co-invests with private VCs in local startups, reducing risk for investors. This fund can be structured as a public-private partnership, inviting national venture firms to create Chengdu-focused funds by offering to match investments or guarantee certain returns. Also, tax incentives such as *tax holidays for new high-tech enterprises* (say, zero profit tax for first 2 years, then a reduced rate for next 3) could attract entrepreneurs. To draw external capital’s attention, Chengdu can organize annual **Western China Innovation Summit** showcasing local startups to investors (leveraging the city’s appeal as a cultural hub, e.g., combining tourism with business networking). - **Mentorship and Corporate Partnerships:** Forge a program linking **local SOEs and established industries with startups** for pilot projects and mentorship. For example, Chengdu’s large manufacturing firms (in automotive, aerospace, electronics) or public utilities could commit to being beta customers for solutions from Chengdu startups, providing valuable testbeds and feedback. Simultaneously, experienced managers from these firms can mentor startup founders on industry knowledge and scale-up challenges. The municipal government can formalize this through MoUs or incentives for SOEs that support startups (like recognizing such mentorship in their performance metrics or giving them priority in government procurement if they partner with local SMEs). - **Policy Streamlining:** Simplify business procedures: ensure quick business registration (Chengdu has improved this recently), and more importantly, make tech-specific rules friendlier – e.g., fast-track certifications for new products developed by local startups, sandbox regulatory environments for things like autonomous vehicle testing or fintech apps so that startups can trial innovation legally. Chengdu has shown its willingness to experiment (for instance, with AI innovation zones[[34]](https://www.amchamchina.org/ai-innovation-zones-in-china-opportunities-for-foreign-investors-2/#:~:text=AI%20Innovation%20Zones%20in%20China%3A,Industries%20in%20the%20Western)); extending this ethos to broad startup activity by removing undue restrictions will help. One idea is a “**green channel**” for procurement: city government could set aside a small portion of its procurement budget to buy from local innovative SMEs (even if they don’t meet all typical criteria), giving them revenue and validation.

**Targeted Scope and Conditions:** This set of measures targets **potential and nascent local entrepreneurs, university talent, and SMEs** in Chengdu, across AI, ICV, FOC, and SC fields. It also involves existing actors like universities, large companies, and investors as supporters and partners. A condition for success is building a more entrepreneurial mindset in a region traditionally dominated by academia and SOEs. This cultural shift may require consistent signals from government and university leadership that entrepreneurship is valued. Chengdu’s populace is increasingly open to this (the city has a vibrant startup scene in gaming and culture tech already), so leveraging that optimism will help. Another condition is patience and tolerance for failure – not every startup will succeed, and some funding will not yield returns; the government and public need to see beyond immediate results to the long-term ecosystem building.

**Risks and Mitigation:** There’s a risk of *talent churn* – i.e., even if startups form, they might be acquired and moved to Tier-1 cities or lose talent to higher salaries elsewhere. Mitigation involves making Chengdu an attractive living and business environment (which it is, relatively affordable and livable). The city might also negotiate with companies like Huawei to keep certain spinoffs or projects local to maintain cluster coherence. Another risk is overlapping initiatives causing inefficiency – thus, coordination by a central body (say, a Chengdu Innovation and Entrepreneurship Council comprising government, academia, industry reps) could ensure programs complement each other.

Over a horizon of 5-10 years, success indicators would include a significant increase in the number of high-tech SMEs in Chengdu, higher retention of STEM graduates in local jobs, several startups from Chengdu reaching notable size or attracting major funding, and perhaps one or two “unicorn” companies emerging in fields like AI software or semiconductor design. These outcomes will feed back into Chengdu’s RIS, providing role models and eventually becoming mentors or acquirers for the next generation, thus solidifying a **self-sustaining enterprise ecosystem**. In sum, by fostering homegrown enterprises, Chengdu can transform its raw innovation potential into realized innovative output, ensuring that the region’s development is not just driven by external or top-down actors, but by a vibrant local entrepreneurial engine.

#### Strengthen Inter-Regional Innovation Linkages to Leverage National and Global Knowledge Flows

**Policy Basis and Justification:** Chengdu’s position as a leading city in western China means it stands to gain significantly from stronger connections to broader innovation networks, both within China (notably the Chengdu–Chongqing twin-city economic circle, and linkages to coastal tech hubs) and internationally (given Chengdu’s improving global ties, e.g., consulates and multinational branch offices). The Regional Differentiation Summary hints at Chengdu’s collaborations with Beijing and Hong Kong researchers in publications, and the city’s success in attracting projects from national tech giants suggests deliberate outreach efforts. However, to accelerate its innovation pathway, Chengdu can further institutionalize these linkages. The policy basis is recognizing that **innovation is increasingly an inter-regional game**: no city can develop frontier tech in isolation. Chengdu could amplify its strengths and compensate for weaknesses by tapping into external expertise and markets. For instance, while Chengdu is strong in research, it might lack cutting-edge industrial design or marketing know-how that exists in Shanghai/Shenzhen; conversely, Chengdu offers unique testing environments and talent that could benefit others (like its embodied AI robotics testbed of national significance[[32]](https://en.people.cn/n3/2025/0722/c90000-20343456.html#:~:text=Based%20in%20the%20city%20of,setting%20and%20commercialization)). Enhancing linkages aligns with national strategies too – the Chinese government encourages west-east cooperation (like pairing hospitals, universities between regions). For Chengdu, a more connected approach would support **technology transfer** from advanced regions, help local innovations scale beyond the local market, and integrate Chengdu’s firms into global value chains. Specifically, bridging Chengdu and Chongqing’s innovation systems could create a metropolitan cluster effect, while connecting to Beijing’s AI hub or Shenzhen’s hardware ecosystem can bring in fresh opportunities. Thus, this policy aims to transform Chengdu’s regional innovation system from a relatively self-contained one into an **open and networked RIS**, increasing its absorptive capacity for external knowledge and opportunities for outward commercialization.

**Mechanism-Level Reasoning:** This recommendation leverages **network theory and RIS extension** – by creating structured channels for knowledge exchange, joint ventures, and mobility, Chengdu’s innovation actors can gain access to a wider pool of ideas and resources. Mechanisms include establishing **joint research and development programs** with institutions in other regions, and promoting **mobility of talent** such as visiting professorships or entrepreneurial exchanges. Such mechanisms work on the principle of **interactive learning** beyond local boundaries, which can prevent lock-in to local trajectories and infuse diversity into Chengdu’s innovation approaches. For example, a joint AI project with a Beijing lab on AGI could bring Chengdu researchers up-to-date insights, while they contribute applied strengths. Another mechanism is integrating into **global innovation networks**: encouraging local firms and researchers to participate in international collaborations, conferences, and standard-setting bodies. This can correct any **institutional thinness** by linking Chengdu’s still maturing institutions to more experienced ones elsewhere (for instance, learning best practices of tech transfer from Tsinghua or of startup incubation from Silicon Valley partners). Additionally, strong inter-regional links can help address **market and financial constraints**: a Chengdu startup could access venture capital in Shanghai or customer bases nationwide more easily if formal connections exist (like a Yangtze-River Delta startup network that Chengdu can plug into). The underlying RIS concept is expanding the **regional innovation boundary** – treating external actors as part of an enlarged system (sometimes called an innovation network approach) rather than strictly focusing within geographic boundaries. This will inject new knowledge, reduce duplication (Chengdu can focus on its niches and rely on others for complementary tech), and enhance the **system’s resilience** through diversity of inputs.

**Implementation Path and Policy Instruments:** - **Chengdu–Chongqing Innovation Corridor:** Given the national support for Chengdu-Chongqing integrated development, create a formal **innovation alliance between Chengdu and Chongqing**. This could include sharing research facilities (e.g., allowing startups from one city to use the other’s lab infrastructure), coordinating industrial plans to avoid overlap and instead specialize (Chongqing might focus on smart manufacturing and automotive production, while Chengdu on AI and design, with both exchanging results), and funding joint projects (with both cities’ governments contributing). A concrete step could be a “Twin-City Tech Innovation Zone” with preferential policies for companies that have operations in both cities (encouraging firms to set up dual bases to exploit each city’s strengths). This will effectively create a larger talent and market pool and could elevate the combined region to rival coastal hubs in innovation output. - **Intercity Talent Exchange and Joint Appointments:** Institute programs that allow easier flow of human capital. For example, support **dual appointments** of top researchers between Chengdu universities and say Beijing or Shanghai institutions – a professor could spend part of the year in each place with funding from a special grant. Similarly, a **“Western Lightning Internship”** scheme might send Chengdu’s young entrepreneurs or students to Shenzhen or overseas tech companies for short stints and bring people from those hubs to Chengdu firms, cross-pollinating ideas. The city can cover costs or provide stipends to lower barriers for SMEs to participate. Over time, these individuals form personal networks bridging regions, which informally fosters knowledge transfer. - **Joint Innovation Funds and Competitions:** Collaborate with richer coastal provinces or national ministries to create joint funding vehicles that invest in projects in Chengdu. For instance, a **Beijing–Chengdu AI Collaborative Fund** could be set up (possibly with central government help) to finance research or startups that involve teams from both cities. Also hold national-level innovation contests or “tech stars” competitions in Chengdu, inviting startups and researchers nationwide to solve problems relevant to Sichuan’s economy (agritech, disaster management tech for earthquakes, etc.). This brings talent and attention from outside into Chengdu and could entice some to stay or partner locally. An example instrument: an annual **International AI Expo** in Chengdu paired with a global hackathon on smart city solutions – leveraging Chengdu’s open testbed environment to attract world teams to try ideas here. - **Global Innovation Outposts:** Encourage Chengdu’s top institutions and parks to set up small outposts in global innovation centers (e.g., a Chengdu Hi-Tech Zone rep office in Silicon Valley or Cambridge, or UESTC research collaboration office in Europe). These outposts can scout for technology trends, facilitate international collaborations, and promote Chengdu’s opportunities to global companies. They act as listening posts and bridgeheads for Chengdu’s integration into global R&D networks. Simultaneously, create more foreign partnerships at home: e.g., invite renowned foreign universities to set up joint institutes in Chengdu (similar to how Shenzhen has joint campuses). Chengdu has started on this with, say, the Sichuan University-Pittsburgh Institute; scaling such efforts diversifies knowledge sources. - **Standards and Policy Alignment:** Work to align Chengdu’s innovation-friendly policies with national and international standards, making it easier for local innovations to be exported. For instance, if Chengdu develops new protocols in smart transport, ensure they are interoperable with what’s being used in other leading cities or contribute Chengdu’s insights to national standards committees (Sichuan could champion standards in earthquake early-warning tech or panda-inspired green tech – areas unique to region – giving it a say nationally). This raises the profile of Chengdu’s innovations and invites collaboration rather than isolation. It also ensures that, regulatory-wise, products made in Chengdu can immediately access broader markets.

**Targeted Scope and Conditions:** This policy suite targets **institutional and network levels** of Chengdu’s innovation system. It involves the city and provincial governments acting as facilitators, and participants include universities, research institutes, startups, established companies in Chengdu, plus counterpart entities in partner regions (other Chinese cities, and global centers). The conditions for success include political support for collaboration (Chengdu will need to be proactive in reaching out, sometimes bridging administrative boundaries or competitive mindsets with other cities). Fortunately, there’s impetus at national level for the west to learn from the east, and Chengdu is often showcased as a model for inland innovation, so it has good standing to initiate partnerships. Another condition is effective coordination – the foreign and intercity programs must have clear goals (not just MoUs with no action). Setting up measurable targets (like number of joint projects launched, cross-city patent co-applications, etc.) can keep the collaborations accountable.

**Risks/Limitations:** One risk is that too much focus on external links could under-emphasize solving internal issues (like one might collaborate a lot but still not fix local tech transfer). This is why this recommendation complements the earlier one: first strengthen local enterprise base, then simultaneously broaden connections – the two reinforce each other (local firms will benefit from external links when they exist). Another risk is sharing benefits: Chengdu must ensure it isn’t just providing raw talent to others without capturing value (for example, if all the best ideas still get commercialized elsewhere). That’s mitigated by the enterprise development push and by structuring joint initiatives to ensure some IP or economic gains flow back to Chengdu (e.g., co-owned IP rights, or establishing project offices in Chengdu).

If executed properly, within a few years Chengdu might see increased co-authored high-impact publications with top-tier coastal universities, more venture deals involving both Chengdu and coastal investors, and even co-founded companies bridging regions. In a decade, one could envisage a scenario where Chengdu and Chongqing together attract a major chunk of west-focused tech investment and perhaps jointly host something like a “West Tech” Davos equivalent, cementing their network role. More directly, Chengdu’s own innovations (like an AI system for earthquake warning or a new photonic chip from UESTC) could find easier pathways to national rollout because of these linkages. Such integration ensures Chengdu’s innovation system is **porous and dynamic**, constantly refreshed by ideas and resources from outside while projecting its own strengths outward – a formula for long-term vitality in an increasingly collaborative innovation landscape.

## Tianjin

### Regional Differentiation Summary

Tianjin’s innovation pathway reflects the city’s legacy as a heavy industrial and engineering center striving to transition into a knowledge-driven economy. Among the four focal industries—**AI, fiber-optic communications (FOC), intelligent connected vehicles (ICV), and storage chips (SC)**—Tianjin’s performance is moderate, without a singular high-profile niche, but with pockets of strength in line with its established industrial base and emerging policy priorities. A prominent feature of Tianjin’s innovation system is the dominance of its two flagship universities, Tianjin University and Nankai University, which together account for a large share of the city’s scientific output across domains. For example, in **AI**, Tianjin University produced over 2,000 papers from 2016–2025, far outpacing other local institutions, and Nankai contributed another substantial portion. These universities, along with affiliated institutes, underpin Tianjin’s R&D – Tianjin University excels in fields like control engineering and applied AI (e.g., AI in chemical processes, given its engineering focus), while Nankai, traditionally strong in mathematics and optics, contributes fundamental research (e.g., algorithms, theoretical computer science). Despite this strong academic core, Tianjin lacks a correspondingly vibrant high-tech corporate sector: few leading AI or tech companies are headquartered in Tianjin, and the city’s industrial innovation has historically been tied to **state-owned enterprises (SOEs)** in sectors such as aerospace (launch vehicles), petrochemicals, and automotive (FAW-Toyota JV). This configuration means **innovation actors are somewhat compartmentalized** – universities leading in publications, and SOEs focusing on process and incremental innovation, with fewer agile private startups bridging the two. The effect is visible in the data: Tianjin’s share of frontier keywords in AI is slightly lower than peers (16.4% by 2025), suggesting that while research exists, it may lean more towards established areas or is slower to pivot into new paradigms like LLMs. Indeed, Tianjin’s top AI frontier terms include “attention mechanism” and “brain-computer interface”, similar to other cities, but with lower counts, which could imply either smaller research groups or less emphasis on those cutting-edge topics historically. However, recent developments indicate Tianjin’s intent to catch up: in 2023, Tianjin opened a major **AI computing center in partnership with Huawei, integrating the latest DeepSeek AI models**[[25]](https://www.scmp.com/news/china/politics/article/3298826/chinas-tianjin-city-embraces-deepseek-part-rush-embrace-domestic-ai-industry#:~:text=The%20Tianjin%20AI%20Computing%20Centre%2C,DeepSeek%E2%80%99s%20models%2C%20the%20report%20said)[[26]](https://www.scmp.com/news/china/politics/article/3298826/chinas-tianjin-city-embraces-deepseek-part-rush-embrace-domestic-ai-industry#:~:text=second). This positions Tianjin to leapfrog in AI capabilities by providing high-performance computing resources for local use (important for training large models). The DeepSeek adoption, as reported, also underscores Tianjin’s alignment with national AI strategies (DeepSeek being a Chinese alternative to Western AGI approaches) and its willingness to invest in infrastructure to attract or retain AI talent[[35]](https://www.scmp.com/news/china/politics/article/3298826/chinas-tianjin-city-embraces-deepseek-part-rush-embrace-domestic-ai-industry#:~:text=China%E2%80%99s%20northern%20port%20city%20of,Chinese%20artificial%20intelligence%20system%20DeepSeek)[[25]](https://www.scmp.com/news/china/politics/article/3298826/chinas-tianjin-city-embraces-deepseek-part-rush-embrace-domestic-ai-industry#:~:text=The%20Tianjin%20AI%20Computing%20Centre%2C,DeepSeek%E2%80%99s%20models%2C%20the%20report%20said). The interplay between actor configurations here is that Tianjin, perhaps lacking organic tech giants, leverages partnerships with national champions (like Huawei) to boost its innovation apparatus.

In **FOC**, Tianjin’s profile is that of a competent but not leading player. The city’s main contributors are again Tianjin University and Nankai University, which have respectable optics and communications labs (Tianjin University’s precision instrument and opto-electronics programs, for example, are well-regarded). Their output led Tianjin’s FOC publications to grow to around 215 in 2025, with research on optical fiber sensors, photonics, and network technology. Top local institutions in FOC include also Tianjin University of Technology and Hebei University of Technology (the latter physically in Tianjin), pointing to a technical focus on applied engineering. A distinctive element of Tianjin’s FOC landscape is the presence of national research bodies: for instance, Tianjin hosts branches of CAS and some key labs in marine communications (note the collaboration with Southern Marine Science Lab in Guangdong seen in co-authorship, hinting at Tianjin involvement in undersea fiber or marine optical sensing research). Tianjin’s port and maritime orientation might influence specific FOC interests, such as *fiber-optic sensing for ships or port monitoring*. However, Tianjin does not have large FOC manufacturing firms like YOFC in Wuhan; any industrial FOC work is likely within larger conglomerates (e.g., within an aerospace or military communications institute). Consequently, **technology transfer** from lab to market in FOC might be less straightforward, which could be why Tianjin’s FOC frontier research is somewhat limited to photodetectors and one mention of “fiber delay line”, lacking diversity. The **strength** here is Tianjin’s solid base in optical science (one of China’s earliest lasers was built in Tianjin University decades ago), and its weakness is the absence of a commercialization engine. The **structural logic** is that Tianjin historically fed its innovations into larger national projects (defense, etc.) rather than spinning off new enterprises. That said, Tianjin’s ongoing initiative to support 6G and “third-generation semiconductors” (explicitly mentioned in its Optics Valley counterpart policy[[1]](https://www.yicaiglobal.com/news/wuhans-high-tech-industry-hub-offers-incentives-of-up-to-usd14-million-per-project-to-lure-global-talent#:~:text=The%20fund%20will%20focus%20on,tech%20industry%20hub) though that was Wuhan, similar strategies are seen in Tianjin’s tech plans) suggests a desire to pivot its FOC knowledge into emerging areas like *terahertz communications or compound semiconductor photonic devices*. But actual results of such policies have yet to fully materialize in the data.

Tianjin’s involvement in **intelligent connected vehicles (ICV)** is an extension of its automotive sector heritage, but here the city faces stiff competition from other regions. Historically, Tianjin was an automotive manufacturing hub (home to Tianjin Xiali in the past, and current FAW-Toyota and other assembly plants). In terms of innovation, the city’s academic institutions (like Tianjin University’s mechanical and vehicle colleges and the Civil Aviation University in avionics/automation) contribute research on control systems, new energy vehicles, etc. Publication counts for Tianjin-ICV are the lowest among the four cities, reaching 105 in 2025, reflecting that until recently, ICV R&D was not a major focus. However, Tianjin has sought to change that by hosting events like the World Intelligence Congress and promoting itself in AI and smart vehicles. A noteworthy move was establishing a National Intelligent Connected Vehicle Pilot Zone (the Northern ICZ) in Tianjin’s Sino-Singapore Eco-City around 2019. This gave companies like Baidu and Pony.ai a site in Tianjin to test autonomous cars. Also, Tianjin has a strong base in **aerospace navigation and guidance** (via local research institutes), which could be leveraged for vehicle autonomy algorithms. The data shows Tianjin’s ICV research touches frontier elements like LiDAR, transformers for driving AI, and ADAS, likely through collaborations with national efforts or adoption of ideas trialed elsewhere. The city’s ICV differentiation is not pronounced, but if anything, Tianjin’s advantage is its proximity to Beijing’s auto tech ecosystem (Baidu Apollo’s main operations are in Beijing, only ~120 km away, which facilitated easy extension to Tianjin’s pilot zone). The local government’s emphasis on “smart mobility” is tied to a broader push to modernize its manufacturing. Perhaps where Tianjin stands out is the integration of ICV with **smart city infrastructure**; Tianjin’s newer districts were built with good infrastructure that can support V2X communication. However, due to the late start, Tianjin’s pathway appears to be one of *adoption and adaptation* of external ICV innovations rather than indigenous innovation. For instance, the largest robotaxi fleet in Tianjin is also run by Baidu or other external firms, unlike Wuhan which also involved a local SOE (Dongfeng). The underlying mechanism is that **Tianjin’s innovation actors in automotive (like FAW-Toyota or Tianjin Bus Corp.) are more implementers than innovators**, so the city encourages partnerships (like with DeepSeek/Huawei for the computing center, which could benefit autonomous driving R&D with its 300-PFlops support[[25]](https://www.scmp.com/news/china/politics/article/3298826/chinas-tianjin-city-embraces-deepseek-part-rush-embrace-domestic-ai-industry#:~:text=The%20Tianjin%20AI%20Computing%20Centre%2C,DeepSeek%E2%80%99s%20models%2C%20the%20report%20said)).

In **storage chips (SC)**, Tianjin has relatively little to show in academic output (virtually no SC keywords recorded, aligning with negligible publication counts). However, Tianjin’s industrial history in semiconductors is notable: it hosted one of China’s earliest semiconductor fabs under Motorola (which became NXP) and is home to *Tianjin Zhonghuan Electronics*, a major silicon wafer and solar PV company. While these are not directly storage chips, they indicate a foundation in materials and manufacturing. In recent years, there were ambitious plans (since stalled) for a large fab (e.g., a few years ago, a company called **Global Foundries** was rumored to partner on a Tianjin fab but that didn’t materialize[[36]](https://www.exploreintel.com/chengdu#:~:text=as%20the%20Chengdu%20Chipset%20Operations,year%20later%2C%20Intel%20Chengdu)). Tianjin’s approach in semiconductors has been cautious; lacking its own YMTC, it instead invested in related areas: for instance, power electronics (with links to its automotive push) and **third-generation semiconductors** like gallium nitride, partly evidenced by initiatives mentioned in city plans. Also, Tianjin established a “National Advanced Microelectronic Innovation Center” which is more on packaging and testing technology. The city thus has potential strength in the *later stages of chip value chain* (materials, packaging) rather than chip design or memory innovation. That might not reflect in publications but is significant for industry. The city’s share of frontier tech in SC is basically zero, highlighting that **Tianjin isn’t at the forefront of memory or logic chip R&D** currently. This is a strategic weakness recognized by policymakers, which is why Tianjin now offers incentives to attract semiconductor projects (in 2022 it announced funds and parks for IC). The structural challenge is heavy competition from established chip hubs (Shanghai, Shenzhen, Wuhan); Tianjin’s differentiation might have to come from integrating chips into its strong industries like aerospace (e.g., radiation-hardened chips for satellites could be a niche) or leveraging its ports to develop semiconductor logistics and trading. At present, though, Tianjin’s innovation pathway in SC is nascent and primarily policy-driven, lacking a champion enterprise or research breakthrough to point to.

From an institutional perspective, Tianjin’s innovation governance is shaped by its role as a municipality with significant state presence. It has numerous national research institutes (often defense or heavy industry-oriented) which produce innovation but perhaps not in a market-facing way. For example, Tianjin is where the **National Supercomputer Center** (that produced the Tianhe-1A supercomputer) is located[[37]](https://en.wikipedia.org/wiki/Tianhe-1#:~:text=Located%20at%20the%20National%20Supercomputing,October%202010%20to%20June%202011). That was a major scientific feat in 2010 and demonstrates Tianjin’s capacity in big science projects. The Tianhe series and related big-data computing achievements (Tianhe’s top ranking in energy-efficient big data processing[[38]](https://www.chinadaily.com.cn/a/202411/22/WS673fd312a310f1265a1cef72.html#:~:text=Tianhe%20supercomputer%20tops%20big%20data,The%20last%20time%20Tianhe)) are emblematic outputs – they show high-end innovation but tied to government labs. This hints at an innovation system still **somewhat top-down and defense-oriented**. The city is now consciously trying to pivot to a more open, enterprise-led model, learning from places like Shenzhen. Policies such as setting up free trade zones, encouraging fintech in the Yujiapu district (Tianjin attempted to build a financial center), and hosting global forums (like the Summer Davos in past years) indicate Tianjin’s eagerness to be seen as a modern innovation-friendly city. Yet, latent mechanisms hindering it include bureaucratic rigidities and an entrenched SOE culture, which can stifle entrepreneurship. Comparatively, Tianjin has not produced homegrown tech giants (its biggest companies are often branches of state enterprises), so the **actor mix lacks dynamic private sector leaders**. However, Tianjin’s proximity to Beijing is an advantage: it can serve as an application and manufacturing base for innovations from the capital (for instance, many aerospace manufacturing tasks are in Tianjin for Beijing-designed rockets). One can foresee that as Beijing saturates, Tianjin could absorb spillover high-tech activities.

In summary, Tianjin’s innovation pathway is best characterized as **transitional and supportive** – the city supports national innovation efforts with its engineering capacity, and is transitioning from a secondary, manufacturing-heavy role towards a more self-sustained innovator. Its strengths lie in broad engineering expertise, established industrial infrastructure, and emerging big projects like the Huawei AI center. Its weaknesses include relatively few breakthrough innovations originating locally and a need to cultivate a more vibrant startup and private sector environment. The **regional differentiation** for Tianjin, vis-à-vis Wuhan, Hangzhou, Chengdu, is that Tianjin is the only one among them deeply embedded in the Beijing-Tianjin-Hebei (Jing-Jin-Ji) mega-region. This gives it unique opportunities (close ties to Beijing’s tech but cheaper space) and challenges (often overshadowed by Beijing). The interplay of actors in Tianjin – strong universities, numerous but inward-focused SOEs, and recently involved external tech firms – has created an innovation system that is substantial in capacity but somewhat under-exploited in terms of output. The policy and institutional environment is now focused on remedying that by attracting frontier projects and loosening old structures, aiming to transform Tianjin into a northern coastal innovation hub that complements Beijing’s R&D might with its own strengths in manufacturing and applied tech. The next steps for Tianjin’s trajectory will likely determine whether it can truly differentiate itself with specialties (perhaps in intelligent manufacturing and aerospace tech) or continue to play a supporting role in China’s innovation landscape.

### Policy Recommendations

#### Accelerate Industrial Modernization through AI and Smart Technologies in Tianjin’s Manufacturing Heartland

**Policy Basis and Justification:** Tianjin’s economic backbone has long been its heavy industries and manufacturing enterprises, including petrochemicals, automotive assembly, machinery, and aerospace. The Regional Differentiation Summary highlights that Tianjin’s innovation outputs have not kept pace with those of more tech-centric cities, due in part to a **lag in infusing cutting-edge technologies** into its traditional sectors. To address this, a policy focus on **industrial modernization via AI and smart tech** is warranted. The basis for this recommendation lies in observed gaps: Tianjin’s AI research, while present in academia, has not deeply penetrated its manufacturing firms, many of which remain reliant on older processes. Meanwhile, Tianjin’s peers (e.g., Shenzhen, Shanghai) have aggressively adopted AI, IoT, and advanced automation in factories, leading to productivity gains and new product lines. Tianjin risks industrial stagnation if its factories and SOEs do not adopt **Industry 4.0** practices, especially since national policy (e.g., the Made in China 2025 initiative) calls for intelligent manufacturing transformations. Moreover, Tianjin’s modest showing in emerging domains like ICV and SC suggests that by modernizing its manufacturing base, it could also create a foundation for new high-tech industries (for example, smart factories making components for EVs or advanced chips). Thus, the policy’s justification is twofold: revitalizing Tianjin’s traditional industries and creating conditions for indigenous innovation by making firms more tech-savvy and open to adopting research outputs from local universities. Essentially, it’s about unleashing *latent demand* for innovation within Tianjin’s industrial heartland. This aligns with RIS thinking that strong **user-producer interactions** can stimulate innovation—Tianjin has producers (factories) that could be better users of AI and digital tech, thereby feeding back to local tech developers.

**Mechanism-Level Reasoning:** This policy will operate through mechanisms that build **capability and connectivity** within Tianjin’s RIS. One mechanism is by implementing demonstration projects of **AI integration in manufacturing**, which serve as learning laboratories that can be replicated across sectors. By showing successful use-cases (e.g., an AI-optimized assembly line at a Tianjin car plant), other firms in the region learn and gain confidence to follow suit – a process of **collective learning** and diminishing perceived risk. Another mechanism is improving **knowledge flow between tech experts and factory floor practitioners**. Historically in Tianjin, such flows might be weak: engineers in plants may not be aware of what local AI researchers can offer, and vice versa. Creating formal liaison programs (embedding data scientists into factories, etc.) fosters **interactive innovation** where problems meet solutions iteratively. In innovation system terms, this addresses an **institutional failure**: the siloing of research and industry. Additionally, by modernizing production, firms can generate new data and technical challenges that spur further R&D in Tianjin’s universities (e.g., complex scheduling algorithms or predictive maintenance models specifically for petrochemical processes). Thus, the policy will initiate a virtuous cycle of **demand-driven innovation**. It also leverages **path dependency** positively: Tianjin’s path is heavy industry; rather than trying to create a wholly new path ex nihilo, we take that existing strength and upgrade it with AI and digital tech, essentially moving the city onto a higher tech trajectory but rooted in what it already does well. This is a path-extension mechanism that often works because it doesn’t require inventing new industries from scratch but significantly elevates productivity and innovation potential in current ones. Moreover, success here can gradually change the **culture and absorptive capacity** of Tianjin’s firms, making them more proactive in seeking out new technologies (and perhaps more attractive for tech talent to join), which is crucial for sustaining innovation.

**Implementation Path and Policy Instruments:** - **Smart Factory Pilot Program:** Launch a comprehensive program to convert a selection of Tianjin’s key factories into model “smart factories”. Choose representative sectors – for instance, one auto assembly plant, one refinery/petrochemical facility, one aerospace component plant, and a large equipment manufacturer – and invest in retrofitting them with IoT sensors, industrial robots, digital twin systems, and AI analytics. The government can subsidize part of the costs and partner these factories with technology providers (e.g., Huawei or CAS institutes specialized in automation). Each pilot should have clear metrics (increase in productivity, reduction in downtime, quality improvement, etc.). Encourage local solution providers (perhaps spin-offs from Tianjin University’s automation school) to contribute, thereby also stimulating local tech businesses. As results materialize, hold open days and publish case studies to disseminate the lessons to other Tianjin firms. - **Industrial AI Task Force and Advisory Service:** Create an expert task force drawn from Tianjin’s universities (AI experts from Tianjin Univ. and Nankai), institutes like the National Supercomputing Center, and experienced engineers from leading firms. This task force acts as roving consultants that can visit factories, diagnose potential areas where AI/automation can help, and connect them with the right tools or providers. Essentially a publicly funded “extension service” for industrial innovation, akin to agricultural extension but for factories. They could run an **Industrial AI Clinic** that companies can approach with specific problems (predictive maintenance, supply chain optimization, etc.), and the task force then proposes solutions and helps implement pilot projects. This builds a bridge for knowledge transfer into industry and builds trust as companies see tailored advice rather than generic slogans. - **Training and Upskilling Programs:** To ensure workforce readiness, partner with vocational institutions and corporate training centers in Tianjin to upskill existing workers and managers in digital competencies. Introduce curricula on data analytics for operations, robot maintenance, and AI basics for engineers. Offer incentives for employees to undertake these trainings (e.g., certifications, career advancement opportunities). At the managerial level, run seminars (perhaps in collaboration with the Tianjin Economic-Technological Development Area, TEDA) showcasing how AI can drive ROI and competitiveness, aiming to shift mindsets of factory directors and SOE managers towards embracing innovation. A well-trained workforce and receptive management are needed to effectively adopt the new technologies from the pilot program. - **Policy Incentives for Adoption:** Use financial and regulatory levers to encourage widespread adoption of successful innovations. For example, offer tax credits or low-interest loans for capital expenditure on automation equipment and AI systems, conditional on firms also investing in worker retraining (to mitigate layoffs fear). Another instrument: **innovation procurement** – Tianjin’s municipal agencies (or SOEs under city influence) can preferentially purchase products or components made in smart factories or require that vendors utilize certain quality AI-driven processes (this way, the supply chain pressures laggards to modernize to keep contracts). Additionally, if pilots prove successful, consider requiring large SOEs to allocate a portion of their annual budget to technology upgrades, framing it as a modernization mandate akin to safety or environmental mandates. - **Monitoring and Feedback:** Establish a monitoring committee to track progress of modernization and address any systemic issues. For example, if integration of certain software with old machinery emerges as a barrier, the city might coordinate with providers to develop retrofitting kits. Or if workers fear job loss causing resistance, involve the union early with reassurance via re-skilling programs or even profit-sharing from efficiency gains. The committee should include government, enterprise, and employee representatives to ensure the modernization push is inclusive and avoids backlash.

**Targeted Scope and Conditions:** This recommendation primarily targets **Tianjin’s large and mid-size manufacturing enterprises and SOEs**, which are the backbone of its economy, as well as the local tech solution providers and research institutes that can serve them. It also indirectly benefits startups (creating local market demand for industrial tech solutions) and academia (by providing real-world problems to solve). Critical conditions for success include buy-in from enterprise leadership – many Tianjin firms are state-controlled, so aligning with SASAC (the body overseeing SOEs) and sectoral ministries to endorse this modernization is important. It may help to incorporate these efforts into the performance evaluation of SOE managers (e.g., adding innovation metrics). Another condition is the safeguarding of labor interests: modernization often triggers automation anxiety. The policy should explicitly aim for **augmentation over replacement**, positioning AI as assisting workers and creating higher-value jobs (like data maintenance, robot supervision) rather than just cutting headcount. Evidence from pilots should be used to show, for instance, that output increased and workers moved to more skilled roles rather than mass layoffs, to maintain social stability and workforce cooperation.

Potential risks: If not managed, firms might be reluctant to invest due to cost or disruption concerns. Government co-investment and demonstration of clear ROI in pilots will mitigate this. Another risk is focusing solely on technology and neglecting process or organizational change – meaning factories install fancy tech but don’t adapt workflows to use it effectively. That is why training and management engagement are emphasized.

If effectively implemented, within 5 years Tianjin should see measurable improvements: productivity in key industries rising, defect rates falling, and cycle times shortening, putting its factories on par with advanced coastal ones. Also, local IT firms specializing in industrial software or AI could grow rapidly, supplying not only Tianjin but other northern regions (thus building a new sector of activity in Tianjin’s economy). In the longer term, this could lead to Tianjin developing a reputation as a leader in smart manufacturing (somewhat akin to how Germany leverages Industry 4.0). Such an outcome reinforces Tianjin’s innovation pathway by *melding its traditional industrial strength with modern technological capability*, ensuring it remains a vital, competitive player in China’s tech-driven growth era.

#### Foster a Collaborative Beijing-Tianjin-Hebei Innovation Cluster to Leverage Complementary Strengths

**Policy Basis and Justification:** Tianjin is uniquely positioned within the **Beijing-Tianjin-Hebei (Jing-Jin-Ji)** region, a national mega-cluster initiative aimed at synergizing the capital’s technological and talent resources with Tianjin’s industrial capacities and Hebei’s space and materials. The Regional Differentiation Summary noted that Tianjin, despite its assets, sometimes plays a secondary role to Beijing, often serving as an application and manufacturing site for innovations originating in the capital. Rather than viewing this as a handicap, a collaborative cluster approach can turn it into an advantage: *joint development, shared resources, and coordinated roles* can propel innovation across the region, with Tianjin carving a clear role that complements Beijing and Hebei. The policy basis is that many challenges and opportunities (air quality improvement tech, regional transportation networks, AI governance, etc.) are regional in nature and best addressed collectively. Moreover, Beijing’s congestion and high costs mean it will inevitably offload some R&D and pilot testing to Tianjin (and Shijiazhuang, etc.). By proactively fostering collaboration, Tianjin can attract and anchor some of that activity, rather than letting it scatter globally or to the Yangtze Delta. Additionally, Tianjin’s moderate showing in frontier innovation could be boosted by formal linkages to Beijing’s world-class research institutions and Zhongguancun tech companies. Conversely, Beijing’s R&D can benefit from Tianjin’s real-world testbeds (e.g., Tianjin’s port for logistics AI, its petrochemical plants for energy tech, etc.). The justification rests on the idea that **the sum is greater than the parts**: a tightly integrated Jing-Jin-Ji innovation cluster can rival the Pearl River or Yangtze River Deltas, and Tianjin stands to benefit immensely by not going it alone. The policy thus seeks to institutionalize collaboration, ensuring Tianjin is not isolated but is an equal partner in a regional innovation system.

**Mechanism-Level Reasoning:** In RIS theory, moving towards a **mega-regional innovation system** involves aligning institutions, harmonizing policies, and facilitating flows of knowledge, people, and capital across administrative boundaries. Mechanisms to do this include **co-investment in major projects**, setting up **cross-regional collaborative platforms**, and removing frictions (bureaucratic, infrastructural) to integration. One mechanism is **specialization with integration**: define complementary roles – for example, Beijing focuses on basic research and high-level design (given its concentration of universities and labs), Tianjin on applied R&D, prototyping, and pilot production, and Hebei on large-scale manufacturing and resource-based innovation (like materials). This avoids duplication and encourages each to innovate in their niche while sharing outputs. Another mechanism is establishing **joint innovation infrastructures** (like labs and data centers accessible to all region researchers, not just one city). The policy would also foster **human capital mobility**: e.g., allowing Beijing researchers to easily take sabbaticals or joint appointments in Tianjin labs and vice versa, smoothing the exchange of ideas. Additionally, a coordinated cluster can implement **unified regulations or standards** that make it easier for firms to operate across the region (for instance, unified rules for autonomous vehicle testing in both Beijing and Tianjin so companies can seamlessly expand pilot programs along the Beijing-Tianjin corridor). This addresses **systemic problems** such as siloed policy and market fragmentation that can hinder innovation diffusion. By viewing Beijing, Tianjin, Hebei as one innovation space (with multiple nodes), it strengthens **network density** (ties among actors across cities) and yields scale effects (bigger talent pool, variety of test environments). For Tianjin, specifically, it can tap into Beijing’s high caliber human resources and financing (Beijing VCs might invest more in Tianjin startups if the cluster is integrated), while Beijing can utilize Tianjin’s infrastructure and less congested environment for expansion. Such synergy corresponds to the idea of **borrowed size** in urban theory: Tianjin can “borrow” some of Beijing’s innovation stature through integration and vice versa Beijing can “borrow” Tianjin’s capacity and space.

**Implementation Path and Policy Instruments:** - **Jing-Jin-Ji Innovation Alliance:** Form a formal regional council or alliance dedicated to innovation coordination, comprising officials from Beijing, Tianjin, and Hebei, as well as representatives of major universities (like PKU, Tsinghua, Tianjin U, Nankai) and enterprises (e.g., Baidu, Huawei’s Beijing branch, Tianjin’s SOEs). This alliance would meet regularly to plan joint initiatives, share progress on each city’s major projects, and identify opportunities for collaboration. One concrete output could be a **Jing-Jin-Ji Innovation Master Plan 2030** that lays out fields for joint focus (AI, biomedicine, environmental tech, etc.) and how resources will be allocated to avoid redundancy and support each other. - **Joint Mega-Projects and Labs:** Identify and fund a few flagship projects that symbolically and substantively embody regional collaboration. For example, establish a **Beijing-Tianjin AI Lab for Urban Solutions** located halfway between the cities (perhaps in Wuqing District or Yanjiao) where researchers from both cities work on smart city algorithms, simulation using Tianjin’s supercomputer data, etc. Or a **Regional Green Energy Tech Center** leveraging Hebei’s renewable sites, with design in Beijing and prototyping in Tianjin. Funding for such labs should come from central government (to incentivize cooperation) and matching local funds, with clear shared governance. Another idea: a **Jing-Jin-Ji Autonomous Mobility Corridor** – upgrading the highway/train corridor between Beijing and Tianjin as a testbed for V2X communication and autonomous driving, allowing companies and research institutes to test technologies on a regional scale (like running robotrucks from Tianjin port to Beijing logistics hubs). This requires coordinated policy (e.g., traffic laws adjustments by both cities) and yields a living lab at mega-region scale, which could attract companies to base related projects in Tianjin or nearby. - **Unified Talent and Capital Policies:** Create a regional “innovation passport” system where entrepreneurs, investors, and researchers registered in one city get equivalent access to incubators, grants, or procurement in the others. For instance, if a startup is based in Tianjin, it should be eligible for Beijing’s innovation subsidies or accelerators without bureaucratic barriers, and vice versa. Similarly, venture funds set up under one city’s scheme could invest across the region seamlessly. The alliance could push for tax-sharing agreements so that if a company’s operations span Beijing and Tianjin, they aren’t double-taxed nor deterred by administrative burden. Essentially treat the region as one for the innovators. This also entails logistics improvements – e.g., continue improving high-speed rail frequency (already strong) and possibly create a *tech bus network* linking Zhongguancun in Beijing with Tianjin’s TEDA and Binhai New Area to ferry personnel daily, thus expanding the effective commuting radius for specialized staff (making it viable for someone to live in one city and work in the other’s tech zone). - **Cross-regional Incubation and Tech Transfer:** Encourage Beijing’s top universities and institutes to establish branches or tech transfer offices in Tianjin. Many Beijing institutions have surplus of startups/projects that they cannot incubate on limited campus space – channel some of these to Tianjin’s tech parks which have more space and perhaps financial perks. Tianjin can offer sweeteners, like free office space or prototyping facilities for startups spun off from Beijing research if they set up in Tianjin. Conversely, let Tianjin University and others have representation in Beijing’s Zhongguancun forums, so their technologies get visibility to the capital’s investor network. Host an annual **Jing-Jin-Ji Innovation Expo** rotating among the cities to showcase startups, patents, and projects region-wide, fostering network ties. The expo could be paired with investment matching, competitions, etc., raising the profile of Tianjin’s innovations as part of a larger narrative rather than judged in isolation. - **Policy Harmonization and Special Zones:** Work towards aligning regulations that impact innovation – for instance, intellectual property enforcement standards, data sharing rules, environmental standards for experiments – so that an innovation-friendly environment is consistent. As an interim step, perhaps create a **Tri-City Special Innovation Zone** (virtually linking key science parks in each city) where agreed-upon liberalized policies apply (e.g., easier cross-border data transfer for research, relaxed rules on testing drones or AVs). This zone would metaphorically cover, say, Beijing’s Haidian, Tianjin’s Binhai, and Shijiazhuang’s High-Tech zone, marking them as collectively open for experimental regulatory approaches (with central government approval likely needed). This could attract companies who know they can pilot something in Tianjin and then smoothly scale it to Beijing within the same regulatory sandbox.

**Targeted Scope and Conditions:** The policy targets **inter-city collaboration** at a broad scale, involving government bodies of the three regions as the main implementers/facilitators. The beneficiaries are ultimately the innovation actors (firms, universities, startups, investors) who get a larger integrated ecosystem to operate in. A crucial condition is political will and trust among the regions – historically, regions can be competitive, but the trend has been towards cooperation under central guidance (the central government’s push for Jing-Jin-Ji integration is an enabling factor). We must ensure Beijing doesn’t overshadow or absorb Tianjin’s identity; the collaboration should highlight Tianjin’s strengths (like manufacturing, port, engineering) in complement to Beijing, not subordinate to it. Clear communication and equitable governance in joint endeavors will be needed. Another condition is securing central government incentives or endorsements – having National-level model projects in the cluster will give it weight and funding.

Risks involve bureaucratic inertia and potential regional protectionism (for example, Beijing might naturally favor its local companies; the policy needs to break that by showing mutual benefits). To mitigate, start with small-scale successes (like a co-run lab) to build trust, and perhaps have central agencies mediate initial projects.

If successful, we would expect within a few years improved metrics such as an increase in collaborative patents between Tianjin and Beijing entities, more investment flow from Beijing VC into Tianjin startups, and a rise in projects that explicitly list both Beijing and Tianjin locations. Over a longer horizon, Tianjin could host branch campuses of Beijing universities or R&D centers of Beijing firms not as a distant satellite, but as part of an integrated expansion (like “one lab, two sites” model). The region might start to be spoken of in one breath in innovation contexts (“the Jing-Jin-Ji AI cluster” analogous to “Silicon Valley”), elevating Tianjin’s stature through association and genuine contribution. In essence, by knitting itself into a regional tapestry, Tianjin can leverage strengths it alone may lack (like top-tier basic research, abundant venture capital) while offering its strengths (space, industrial know-how, pilot environments) to the region, creating a **win-win innovation synergy**. This ensures that Tianjin’s innovation pathway, rather than treading an isolated uphill course, rides a collective momentum where it can both contribute to and benefit from the region’s overall innovation dynamism.

[[1]](https://www.yicaiglobal.com/news/wuhans-high-tech-industry-hub-offers-incentives-of-up-to-usd14-million-per-project-to-lure-global-talent#:~:text=The%20fund%20will%20focus%20on,tech%20industry%20hub) [[2]](https://www.yicaiglobal.com/news/wuhans-high-tech-industry-hub-offers-incentives-of-up-to-usd14-million-per-project-to-lure-global-talent#:~:text=The%20EDZ%20is%20a%20high,percent%20from%20a%20year%20earlier) [[11]](https://www.yicaiglobal.com/news/wuhans-high-tech-industry-hub-offers-incentives-of-up-to-usd14-million-per-project-to-lure-global-talent#:~:text=the%20%27Optics%20Valley%20of%20China%2C%27,for%20a%20single%20project) [[12]](https://www.yicaiglobal.com/news/wuhans-high-tech-industry-hub-offers-incentives-of-up-to-usd14-million-per-project-to-lure-global-talent#:~:text=According%20to%20the%20document%2C%20top,recognized%20as%20future%20tech%20leaders) Wuhan's High-Tech Industry Hub Offers Incentives of Up to USD14 Million Per Project to Lure Global Talent

<https://www.yicaiglobal.com/news/wuhans-high-tech-industry-hub-offers-incentives-of-up-to-usd14-million-per-project-to-lure-global-talent>

[[3]](https://thenetworkinstallers.com/blog/best-fiber-optic-manufacturers/#:~:text=Prysmian%20Group%20%289,of%20the%20world%E2%80%99s%20largest%20fiber) 10 Best Fiber Optic Manufacturers for 2026

<https://thenetworkinstallers.com/blog/best-fiber-optic-manufacturers/>

[[4]](https://www.scmp.com/business/article/3239310/chinas-ymtc-makes-worlds-most-advanced-memory-chip-surprise-technology-leap-techinsights-report#:~:text=Yangtze%20Memory%20Technologies%20Co%20,to%20a%20report%20by%20TechInights) [[5]](https://www.scmp.com/business/article/3239310/chinas-ymtc-makes-worlds-most-advanced-memory-chip-surprise-technology-leap-techinsights-report#:~:text=3D%20NAND%20memory%20is%20at,artificial%20intelligence%20and%20machine%20learning) China’s YMTC makes world’s most advanced memory chip in ‘surprise technology leap’: TechInsights report | South China Morning Post

<https://www.scmp.com/business/article/3239310/chinas-ymtc-makes-worlds-most-advanced-memory-chip-surprise-technology-leap-techinsights-report>

[[6]](https://www.scmp.com/tech/tech-trends/article/3271143/wuhan-driverless-taxis-offer-peek-future-intracity-transport-china#:~:text=While%20cities%20such%20as%20Beijing,a%20pioneer%20of%20the%20technology) [[7]](https://www.scmp.com/tech/tech-trends/article/3271143/wuhan-driverless-taxis-offer-peek-future-intracity-transport-china#:~:text=Operating%20under%20the%20brand%20name,and%20shuttles%20in%20the%20city) In Wuhan, driverless taxis offer a peek into the future of intracity transport in China | South China Morning Post

<https://www.scmp.com/tech/tech-trends/article/3271143/wuhan-driverless-taxis-offer-peek-future-intracity-transport-china>

[[8]](https://english.wuhan.gov.cn/H_1/NWP/202003/t20200316_954175.shtml#:~:text=China%27s%20first%20test%20zone%20for,Demonstration%20City%20in%20Wuhan%2C) China's first test zone for self-driving cars opens in Wuhan

<https://english.wuhan.gov.cn/H_1/NWP/202003/t20200316_954175.shtml>

[[9]](https://cset.georgetown.edu/publication/wuhans-ai-development/#:~:text=approaches%20to%20AGI%20that%20involve,surroundings%2C%20learning%20as%20it%20proceeds) [[10]](https://cset.georgetown.edu/publication/wuhans-ai-development/#:~:text=The%20test%20bed%20for%20this,all%20aspects%20of%20daily%20life) Wuhan’s AI Development | Center for Security and Emerging Technology

<https://cset.georgetown.edu/publication/wuhans-ai-development/>

[[13]](https://en.wikipedia.org/wiki/City_Brain#:~:text=The%20first%20City%20Brain%20system,In%20the%20following) [[14]](https://en.wikipedia.org/wiki/City_Brain#:~:text=years%2C%20many%20other%20local%20governments,1) City Brain - Wikipedia

<https://en.wikipedia.org/wiki/City_Brain>

[[15]](https://autonews.gasgoo.com/articles/news/another-automaker-obtains-an-l3-autonomous-driving-road-test-license-2009306621597822977#:~:text=On%20January%207%2C%20Hangzhou%27s%20Bureau,test%20license) [[16]](https://autonews.gasgoo.com/articles/news/another-automaker-obtains-an-l3-autonomous-driving-road-test-license-2009306621597822977#:~:text=The%20approved%20scope%20covers%20all,validation%20of%20autonomous%20driving%20scenarios) [[17]](https://autonews.gasgoo.com/articles/news/another-automaker-obtains-an-l3-autonomous-driving-road-test-license-2009306621597822977#:~:text=The%20approved%20scope%20covers%20all,validation%20of%20autonomous%20driving%20scenarios) Another automaker obtains an L3 autonomous driving road test license! | Gasgoo

<https://autonews.gasgoo.com/articles/news/another-automaker-obtains-an-l3-autonomous-driving-road-test-license-2009306621597822977>

[[18]](https://hic.zju.edu.cn/hicenglish/82817/list.htm#:~:text=Zhejiang%20Integrated%20Circuits%20Innovation%20Platform,elite%20industrial%20talent%2C%20it) Zhejiang Integrated Circuits Innovation Platform

<https://hic.zju.edu.cn/hicenglish/82817/list.htm>

[[19]](https://www.theguardian.com/technology/2023/nov/11/west-bank-palestinians-surveillance-cameras-hikvision#:~:text=Among%20the%20vendors%20behind%20these,of%20the%20Uyghur%20ethnic%20minority) How Chinese firm linked to repression of Uyghurs aids Israeli surveillance in West Bank | Facial recognition | The Guardian

<https://www.theguardian.com/technology/2023/nov/11/west-bank-palestinians-surveillance-cameras-hikvision>

[[20]](https://www.prnewswire.com/news-releases/chengdu-making-strong-push-to-foster-next-gen-ai-technology-301181080.html#:~:text=In%20September%2C%20Chinese%20communication%20technology,9%20billion%20yuan) [[21]](https://www.prnewswire.com/news-releases/chengdu-making-strong-push-to-foster-next-gen-ai-technology-301181080.html#:~:text=The%20center%20will%20bring%20Huawei%27s,construction%2C%20the%20telecoms%20giant%20said) [[22]](https://www.prnewswire.com/news-releases/chengdu-making-strong-push-to-foster-next-gen-ai-technology-301181080.html#:~:text=construction%2C%20the%20telecoms%20giant%20said) [[23]](https://www.prnewswire.com/news-releases/chengdu-making-strong-push-to-foster-next-gen-ai-technology-301181080.html#:~:text=Image%3A%20Chengdu%20Supercomputing%20Center%2C%20the,started%20trial%20operations%20in%20September) [[24]](https://www.prnewswire.com/news-releases/chengdu-making-strong-push-to-foster-next-gen-ai-technology-301181080.html#:~:text=Chengdu%20Supercomputing%20Center%2C%20the%20first,industry%20growth%20in%20the%20region) [[27]](https://www.prnewswire.com/news-releases/chengdu-making-strong-push-to-foster-next-gen-ai-technology-301181080.html#:~:text=CHENGDU%2C%20China%2C%20Nov,generation%20artificial%20intelligence) [[28]](https://www.prnewswire.com/news-releases/chengdu-making-strong-push-to-foster-next-gen-ai-technology-301181080.html#:~:text=According%20to%20the%20plan%2C%20Chengdu,hub%20and%20several%20industrial%20parks) Chengdu making strong push to foster next-gen AI technology

<https://www.prnewswire.com/news-releases/chengdu-making-strong-push-to-foster-next-gen-ai-technology-301181080.html>

[[25]](https://www.scmp.com/news/china/politics/article/3298826/chinas-tianjin-city-embraces-deepseek-part-rush-embrace-domestic-ai-industry#:~:text=The%20Tianjin%20AI%20Computing%20Centre%2C,DeepSeek%E2%80%99s%20models%2C%20the%20report%20said) [[26]](https://www.scmp.com/news/china/politics/article/3298826/chinas-tianjin-city-embraces-deepseek-part-rush-embrace-domestic-ai-industry#:~:text=second) [[35]](https://www.scmp.com/news/china/politics/article/3298826/chinas-tianjin-city-embraces-deepseek-part-rush-embrace-domestic-ai-industry#:~:text=China%E2%80%99s%20northern%20port%20city%20of,Chinese%20artificial%20intelligence%20system%20DeepSeek) China’s Tianjin city adopts DeepSeek as part of rush to embrace domestic AI industry | South China Morning Post

<https://www.scmp.com/news/china/politics/article/3298826/chinas-tianjin-city-embraces-deepseek-part-rush-embrace-domestic-ai-industry>

[[29]](https://finance.yahoo.com/news/intel-invests-us-300-million-093000185.html#:~:text=Intel%20invests%20US%24300%20million%20in,commitment%20to%20the%20mainland%20market) Intel invests US$300 million in China chip packaging and testing plant

<https://finance.yahoo.com/news/intel-invests-us-300-million-093000185.html>

[[30]](https://en.lrc.cn/about/group.html#:~:text=Chengdu%20Advanced%20Power%20Semiconductor%20Co,production%20and%20sales%20of) Chengdu Advanced Power Semiconductor Co., Ltd

<https://en.lrc.cn/about/group.html>

[[31]](http://gcsemi.cn/en/#:~:text=Products%20,power%20semiconductor%20applications%20in%20China) Products - GCSEMI – Giant Chip Semiconductor

<http://gcsemi.cn/en/>

[[32]](https://en.people.cn/n3/2025/0722/c90000-20343456.html#:~:text=Based%20in%20the%20city%20of,setting%20and%20commercialization) [[33]](https://en.people.cn/n3/2025/0722/c90000-20343456.html#:~:text=The%20project%2C%20officially%20launched%20on,department%20told%20Xinhua%20on%20Tuesday) New AI robot training ground launched in China - People's Daily Online

<https://en.people.cn/n3/2025/0722/c90000-20343456.html>

[[34]](https://www.amchamchina.org/ai-innovation-zones-in-china-opportunities-for-foreign-investors-2/#:~:text=AI%20Innovation%20Zones%20in%20China%3A,Industries%20in%20the%20Western) AI Innovation Zones in China: Opportunities for Foreign Investors

<https://www.amchamchina.org/ai-innovation-zones-in-china-opportunities-for-foreign-investors-2/>

[[36]](https://www.exploreintel.com/chengdu#:~:text=as%20the%20Chengdu%20Chipset%20Operations,year%20later%2C%20Intel%20Chengdu) Chengdu Environmental Performance - Explore Intel

<https://www.exploreintel.com/chengdu>

[[37]](https://en.wikipedia.org/wiki/Tianhe-1#:~:text=Located%20at%20the%20National%20Supercomputing,October%202010%20to%20June%202011) Tianhe-1 - Wikipedia

<https://en.wikipedia.org/wiki/Tianhe-1>

[[38]](https://www.chinadaily.com.cn/a/202411/22/WS673fd312a310f1265a1cef72.html#:~:text=Tianhe%20supercomputer%20tops%20big%20data,The%20last%20time%20Tianhe) Tianhe supercomputer tops big data ranking again - China Daily

<https://www.chinadaily.com.cn/a/202411/22/WS673fd312a310f1265a1cef72.html>
